# 1,2,4-Triazole-Tethered Indolinones as New Cancer-Fighting Small Molecules Targeting VEGFR-2: Synthesis, Biological Evaluations and Molecular Docking

**DOI:** 10.3390/ph17010081

**Published:** 2024-01-08

**Authors:** Ahmed E. Elsawi, Mai I. Shahin, Hager A. Elbendary, Tarfah Al-Warhi, Fatma E. Hassan, Wagdy M. Eldehna

**Affiliations:** 1 Department of Pharmaceutical Chemistry, Faculty of Pharmacy, Kafrelsheikh University, Kafrelsheikh 33516, Egypt; phd.ahmed.elsawi@gmail.com; 2 Department of Pharmaceutical Chemistry, Faculty of Pharmacy, Ain Shams University, Abassia, Cairo 11566, Egypt; maishahin@pharma.asu.edu.eg; 3 Scientific Research and Innovation Support Unit, Faculty of Pharmacy, Kafrelsheikh University, Kafrelsheikh 33516, Egypt; 4 Department of Chemistry, College of Science, Princess Nourah bint Abdulrahman University, P.O. Box 84428, Riyadh 11671, Saudi Arabia; 5 Department of Physiology, General Medicine Practice Program, Batterjee Medical College, Jeddah 21442, Saudi Arabia; fatma.hassan@bmc.edu.sa; 6 Medical Physiology Department, Kasr Alainy, Faculty of Medicine, Cairo University, Giza 11562, Egypt

**Keywords:** angiogenesis, anti-cancer agents, synthesis, molecular modeling, tail approach

## Abstract

Targeting the VEGFR-2 signaling pathway is an inveterate approach toward combating pancreatic and hepatocellular cancers. Based on Sunitinib, the FDA-approved VEGFR-2 inhibitor, novel indolin-2-one-triazole hybrids were designed and synthesized as anti-hepatocellular and anti-pancreatic cancer agents with VEGFR-2 inhibitory activity. All the targeted compounds were assessed for their anti-cancer activity, revealing IC_50_ values extending from 0.17 to 4.29 µM for PANC1 and 0.58 to 4.49 µM for HepG2 cell lines. An extensive SAR study was conducted to explore the effect of different substituents along with *N*-alkylation. The potent anti-cancer analogs **11d**, **11e**, **11g**, **11k** and **14c** were evaluated for their VEGFR-2 inhibitory actions, where their IC_50_ values ranged from 16.3 to 119.6 nM compared to Sorafenib, which revealed an IC_50_ of 29.7 nM, having compound 11d as the most active analog. An in silico ADME study was performed to confirm the drug-likeness of the synthesized compounds. Finally, molecular docking simulation was conducted for the most potent VEGFR-2 inhibitor (**11d**), demonstrating the strong binding with the vital amino acid residues of the VEGFR-2 ATP binding site.

## 1. Introduction

Angiogenesis is the process of recruiting new capillary blood vessels from existing ones. The viability of mammalian cells relies on oxygen and nutrients, prompting them to position themselves at distances of 100 nm to not more than 200 mm from blood vessels, which signifies the oxygen diffusion limit. In order to support the growth of multicellular organisms beyond this threshold, angiogenesis plays a crucial role in recruiting new blood vessels [1]. This intricate process is regulated by a subtle equilibrium between pro- and anti-angiogenic molecules and can be disrupted in numerous diseases, notably cancer [2]. Angiogenesis is switched on when pro-angiogenic factors surmount anti-angiogenic ones. Designated as one of the crucial hallmarks of cancer growth, metastasis and survival, angiogenesis blocking is entrenched as one of the foremost approaches to treating different life-threatening cancer diseases [3,4]. The vascular endothelial growth factor family (VEGF) is one of the most prominent angiogenic activators, is highly expressed in various cancerous cells and plays a pivotal role in neovascularization [5]. The VEGF family is responsible for activating the VEGF receptors (VEGFR-1, VEGFR-2 and VEGFR-3), which, in turn, enhances neoplastic vascularization [5]. VEGFR-1 and VEGFR-2 are closely associated receptor tyrosine kinases and play pivotal roles in physiological and pathological angiogenesis, notably in the context of tumor angiogenesis. Despite being a kinase-impaired receptor tyrosine kinase, VEGFR-1 is actively involved. On the other hand, VEGFR-2, characterized by high activity, emerges as the principal VEGF receptor present on the surface of vascular endothelial cells [3].

The VEGF/VEGFR-2 pathway has gained great attention as a potent therapeutic target in multiple cancers [6]. Since the up-regulation of VEGFR-2 is pronounced in malignant tumors, VEGFR-2 inhibitors have proved their profound anti-proliferative activities in many tumors, including pancreatic [7,8] and hepatocellular cancers [9,10]. Siegel et al. reported that pancreatic cancer is the third foremost cause of death from cancer in both genders [11]. In addition, liver cancer has had the speediest increase in mortality for decades. The current survival rate is lowest for both pancreatic (12%) and liver (21%) cancers. Obviously, 90% of liver cancer cases are stated to be hepatocellular carcinoma (HCC) [12]. Advanced HCC is resistant to both chemotherapy and radiotherapy [13]. Similarly, pancreatic cancer also develops chemoresistance, which hinders the effectiveness of the treatment, imparting poor response and quick relapse [14]. These findings urge the need to develop novel chemotherapeutic agents of greater potency and selectivity with no resistance from the targeted cancerous cells. 

**Figure 1:**
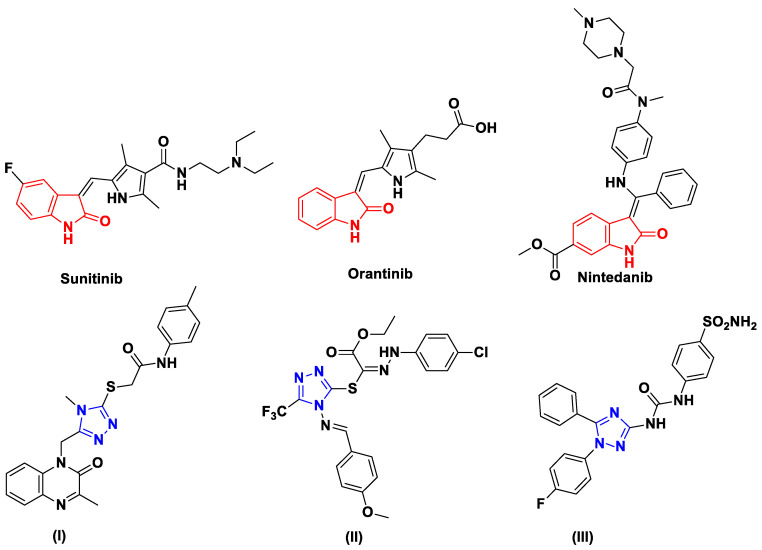
Chemical structures of potent chemotherapeutic agents with significant VEGFR-2 inhibitory activity encompassing indolin-2-one (in red) or 1,2,4 triazole heterocycles (in blue).

Myriad indolin-2-one-based small molecules were developed and evaluated for their anti-proliferative activities, along with VEGFR-2 inhibition [15]. The choice of indolin-2-one scaffold is based on its superior pharmacokinetic properties, ease of synthesis and previously reported anti-tumor activity. This is clearly evinced by having a close look at Sunitinib’s chemical structure. Sunitinib is a marketed VEGFR inhibitor with excellent anti-tumor activities against gastrointestinal stromal tumors, neuroendocrine tumors, renal cell carcinoma and pancreatic and breast cancers, while it is primarily an indolin-2-one derivative [16]. Ornatinib is another chemotherapeutic agent indicated for hepatocellular carcinoma in phase 3 clinical trials with significant VEGFR-2 inhibition, which features an indolin-2-one ring [17]. Moreover, Nintedanib is an indolin-2-one analog that acts as a tyrosine kinase inhibitor suppressing angiogenesis and completed Phase 3 clinical trials combined with Docetaxel to be used in non-small-cell lung cancer [18]. On the other hand, the 1,2,4-triazole ring was explored for its anti-VEGFR-2 inhibition, where compound (I) showed VEGFR-2 inhibition with an IC_50_ of 0.06 μM [19]. In the same context, compound (II) exhibited noticeable cytotoxic activity against hepatocellular cell lines HepG2 (IC_50_ = 10.02 μM) and manifested marked suppression of VEGFR-2 with IC_50_ of 0.074 μM [20]. A novel series of 1,5-diaryl-1,2,4-triazole urea signified its powerful anti-tumor activity through dual inhibitory action against both carbonic anhydrase and VEGFR-2, where compound (III) showed compelling VEGFR-2 inhibition with IC_50_ 26.3 nM [21] (Figure 1). 

The previously reported investigations of the binding mode of Sunitinib in VEGFR-2 revealed that the indolin-2-one ring accommodated the ATP binding site through the establishment of essential hydrogen bonds with Glu917 and Cys919 in the hinge region. Additionally, both the pyrrole ring and the fluoro group performed hydrophobic interactions that enhanced binding affinity. Finally, the diethylaminoethyl represents a hydrophilic tail toward the solvent-accessible region [22].

The strategy of combining biologically active molecules through molecular hybridization is a powerful technique in drug discovery. Its applications extend to various diseases, including AIDS, malaria, leishmaniasis and, of course, cancer. Interestingly, the molecular hybridization of biologically captivating pharmacophores, indolin-2-one included, is an ingrained strategy toward developing novel, potent chemotherapeutic agents that can effectively inhibit VEGFR-2 [23]. The philosophy behind the hybridization is the reported supremacy of the hybridized pharmacophoric entities over the corresponding single ones. Predominantly, the amalgamated compounds possess superior activity, fewer side effects, better pharmacokinetic properties and can evade drug resistance [24]. 

**Figure 2:**
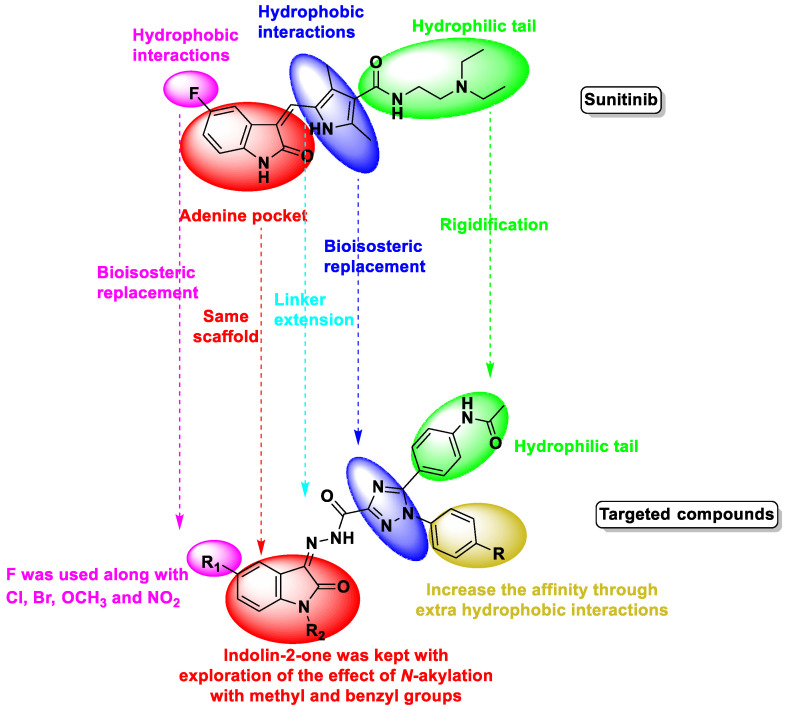
Rational design of the targeted molecules based on Sunitinib interactions with VEGFR-2 receptor.

Inspired by the aforementioned findings and grounded on the potency shown by both indolin-2-one and 1,2,4-triazole counterparts and in a trial to raise potent anti-tumor agents with dominant anti-angiogenic activity, two series of indolin-2-one-1,2,4-triazole hybrids (**11a**–**l** and **14a**–**d**) were designed grounded on the structure–activity relationship (SAR) of Sunitinib. The indolin-2-one scaffold was maintained in the designed compounds due to its importance in adopting the adenine binding pocket and forming the essential interactions with Glu917 and Cys919. To maintain the hydrophobic interactions of the fluorine atom in Sunitinib, it was kept in our designed compounds **11a**–**l** with further use of other substituents (Cl, Br, OCH_3_ and NO_2_) to have deeper insight into their impacts on activity. Additionally, the pyrrole ring was bioisostered with a 1,2,4-triazole ring, where the triazole ring corroborated its potency in many VEGFR-2 inhibitors, as mentioned above. The linker was extended between the indolin-2-one and 1,2,4-triazole rings to be a hydrazide linker. Also, the *N*-substituted triazole ring with a *p*-F/Cl phenyl moiety was adopted in a trial hoping to increase the hydrophobic interactions and, consequently, the affinity of the targeted compounds to the ATP binding site. Besides, acetamide was used as the solvent-accessible tail instead of the diethylamino of Sunitinib (Figure 2). Furthermore, the impact of *N*-alkylation of the indolin-2-one with methyl and benzyl groups on biological activity was considered for exploration in compounds **14a**–**d**. Eventually, the two series of hybrids (**11a**–**l** and **14a**–**d**) were synthesized and biologically assessed for their growth inhibitory activities versus PANC1 and HepG2 cells. In addition, their VEGFR-2 inhibitory action was evaluated and expressed in terms of IC_50_. The ADME properties of the targeted molecules were calculated and evaluated to confirm their pharmacokinetic appropriateness. The final step involved a molecular docking study using the potent VEGFR-2 inhibitor among the test derivatives to figure out the binding modes that led to the inhibitory action.

## 2. Results and Discussion

### 2.1. Chemistry

**Scheme 1:**
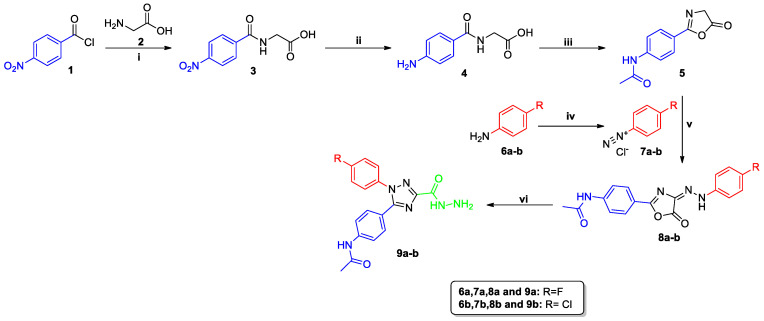
Synthesis of key hydrazide intermediates **9a–b**. Reagents and conditions: (**i**) aq. NaOH, stirring, room temperature, 1 h. (**ii**) Methanol, Pd/C, room temperature, 3 h. (**iii**) Acetic anhydride, heating 75 °C, 40 min. (**iv**) HCl, NaNO_2_, stirring, 0–5 °C, 20 min. (**v**) Sodium acetate, stirring, 0–10 °C, 3 h. (**vi**) Ethanol, NH_2_NH_2_·H_2_O 90%, reflux, 1 h.

**Scheme 2:**
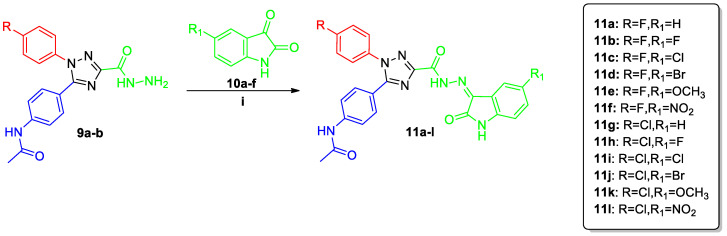
Synthesis of target 1,5-diaryl substituted triazole-indolin-2-one hybrids **11a–l**. Reagents and conditions: (**i**) ethanol, acetic acid, reflux, 2 h.

**Scheme 3:**
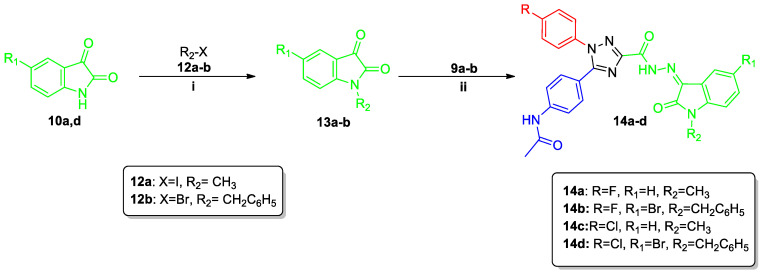
Synthesis of target 1,5-diaryl substituted triazole-indolin-2-one hybrids **14a–d**. Reagents and conditions: (**i**) acetonitrile, potassium carbonate, stirring, room temperature, 18 h. (**ii**) Ethanol, acetic acid, reflux, 2 h.

The synthesis of the targeted 1,5-diaryl substituted triazole-indolin-2-one hybrids **11a**–**l** and **14a**–**d** is illustrated in Scheme 1, Scheme 2 and Scheme 3. 4-Aminohippuric acid **4** was prepared by acylating the amino group of glycine amino acid **2** using *p*-nitrobenzoyl chloride **1** in aqueous sodium hydroxide solution to yield 4-nitrohippuric acid **3** and, subsequently, the nitro group was reduced to the required amino group using Pd/C. To obtain compound **5**, 4-aminohippuric acid **4** was heated with acetic anhydride, resulting in the acylation of two distinct functional groups. The amino group was converted to the corresponding acetamido group, while carboxylic acid was simultaneously activated to form an unstable mixed anhydride, leading to the formation of the azalactone ring containing an active methylene group. The active methylene was then coupled through the Kuskov-like reaction with freshly prepared diazonium salt **7a**–**b** derived from 4-fluoro or 4-chloroanilines **6a**–**b** using sodium acetate salt to provoke hydrazone linker-tethered compounds **8a**–**b**. Subsequently, the azalactone ring was opened and underwent Sawdey rearrangement [25] via refluxing in ethanol with hydrazine hydrate, ultimately resulting in the formation of key intermediates, hydrazides **9a**–**b**, Scheme 1.

Hydrazides **9a**–**b** underwent condensation with the 3-carbonyl group of diverse phenyl-substituted and *N*-alkylated indolin-2-one derivatives **10a**–**f** and **13a**–**b**, respectively, under reflux conditions in absolute ethanol, facilitated by the addition of a minimal catalytic quantity of glacial acetic acid to furnish hybrids **11a**–**l** and **14a**–**d**, Scheme 2 and Scheme 3.

The structural attributes of the newly synthesized hybrids **11a**–**l** and **14a**–**d** were observed to align precisely with the outcomes derived from both spectral and elemental analyses. The ^1^H NMR spectra revealed the presence of distinctive NH protons. The signal associated with NH of the acetamido moiety appeared in the range of *δ* 10.20–10.26 ppm, while the NH signal of the acylhydrazone linker was observed between *δ* 11.70 and 14.32 ppm. Additionally, in derivatives lacking an *N*-alkyl group, the NH signal specific to the indolin-2-one moiety was detected between *δ* 10.69 and 11.91 ppm. Furthermore, a discernible aliphatic signal for the CH_3_ group of the acetamido motif was observed within the range of *δ* 2.05–2.10 ppm.

In the ^13^C NMR spectra, the three carbonyl groups present in the newly synthesized hybrids were distinctly evident. The acylhydrazone C=O signal appeared between *δ* 154.73 and 155.89 ppm, while the C=O signal associated with the indolin-2-one scaffold was observed between *δ* 161.00 and 164.94 ppm. Additionally, the acetamido C=O signal appeared at the highest chemical shift range (*δ* 168.84–169.46 ppm). The aliphatic methyl group of the acetamido group was identified in the range of *δ* 24.11–24.56 ppm. Finally, high-resolution electrospray ionization mass spectrometry (ESI-HRMS) spectra exhibited peaks corresponding to quasimolecular ions [M+H]^+^ and [M+Na]^+^, confirming the successful synthesis of the target compounds.

### 2.2. Biological Evaluation

#### 2.2.1. In Vitro Anti-Proliferative Activity against PANC1 and HepG2 Cell Lines

The anti-tumor activity of the targeted compounds was assessed against PANC1 and HepG2 cell lines. The examined human cancer HepG2 and PANC1 cell lines were obtained from the American Type Culture Collection (ATCC). The resulting IC_50_ values revealed the remarkable inhibition of cell growth in both contexts, with values for PANC1 cell lines ranging from 0.17 to 4.29 µM and for HepG2 cell lines spanning from 0.58 to 4.49 µM. First, for series **11a**–**l**, compounds **11c**, **11f**, **11g**, **11h** and **11l** manifested exceptional anti-proliferative activity against the PANC1 cell line with IC_50_ values at the submicromolar level (0.98, 0.23, 0.77, 0.22 and 0.17 µM, respectively). Moderate activity was evidenced for compounds **11b**, **11d**, **11e**, **11j** and **11k** with IC_50_ values of 1.74, 1.16, 2.22, 1.68 and 1.78 µM, respectively, for the same cell line. The least anti-neoplastic activity against PANC1 was observed for compounds **11a** and **11i**, as their IC_50_ were 4.29 and 3.75 µM, respectively.

On the other hand, potent anti-tumor activity was exhibited versus the HepG2 cell line by compounds **11d**, **11e**, **11g** and **11k**, displaying IC_50_ values in the submicromolar range (0.73, 0.76, 0.71 and 0.73 µM, respectively). All other compounds in the series conveyed appreciable inhibition of HepG2 cell growth with IC_50_ values ranging from 1.01 to 2.41 µM, with the exception of compound **11i**, which showed an IC_50_ of 4.49 µM.

To elucidate SAR, this series can be categorized into those containing *N*-(4-fluorophenyl) (**11a**–**f**) and *N*-(4-chlorophenyl) (**11g**–**l**). The most potent compound inhibiting PANC1 within the fluorinated phenyl group was **11f** (IC_50_ = 0.23 µM), while in the chlorinated phenyl group, it was **11l** (IC_50_ = 0.17 µM). Both compounds notably featured a 5-nitro group in the indolin-2-one ring. It is worth mentioning that **11l** was the most potent inhibitor of cancerous cell growth in this study. For the HepG2 cell line, **11d** (IC_50_ = 0.73 µM) and **11g** (IC_50_ = 0.71 µM) emerged as the most effective in the fluorinated and chlorinated phenyl groups, respectively. Despite the disparity in the substitution pattern on the indolin-2-one ring between the optimal compounds in both subseries—where **11d** features 5-bromo while **11g** is unsubstituted—the second-ranked compounds in inhibitory efficacy against HepG2 from the two subseries are those incorporating 5-methoxy (**11e** and **11k**). Their IC_50_ values (0.76 µM and 0.73 µM, respectively) were very proximal to those of **11d** and **11g**.

For a more nuanced comparative analysis between the two subseries, a clear dominance was observed in the inhibitory activity of compounds containing chlorinated phenyl against both cell lines when the indolin-2-one was either unsubstituted (**11g**) or bears a methoxy (**11k**) or nitro (**11l**) group at position 5. When it featured a fluoro group (**11h**), the efficacy showed an 8-fold improvement against PANC1 compared to its counterpart with fluorinated phenyl (**11b**). However, a slight decrease is noted towards HeG2. On the contrary, the superiority of compounds containing fluorinated phenyl becomes conspicuously evident when position 5 in the indolin-2-one incorporates chloro (**11c**) and bromo (**11d**) groups.

Interestingly, *N*-methyl/benzyl derivatives (**14a**–**d**) unveiled outstanding anti-proliferative activity patterns against both PANC1 and HepG2 cell lines relative to the unsubstituted peers, with IC_50_ values ranging from 0.2 to 4.05 µM for PANC1 and 0.58 to 1.63 µM for HepG2. The compelling analog **14c** expressed distinctive potency against both PANC1 and HepG2 cell lines with IC_50_ values of 0.2 and 0.58 µM, respectively, establishing it as the most effectual growth inhibitor against HepG2 in this study. Furthermore, compounds **14b** and **14d** divulged substantial growth inhibition of the PANC1 cell line with IC_50_ values of 0.76 to 0.42, respectively. Concerning their activity versus HepG2 cell lines, both compounds displayed IC_50_ values equal to 0.98 and 0.92 µM, respectively. However, compound **14a** showed a slight increase in the IC_50_ to be 4.05 for PANC1 and 1.63 for HepG2.

Judging from the displayed IC_50_ values, the compounds featuring chlorinated phenyl (**14c** and **14d**) demonstrate a clear superiority in inhibiting the tested cancer cell lines compared to those containing fluorinated phenyl (**14a** and **14b**). This indicates the importance of having a larger group than fluorine with less of an electron-withdrawing effect to improve the anti-proliferative potency. By scrutinizing the IC_50_ values presented in Table 1 to evaluate the impact of *N*-substitution on the indolin-2-one ring, the positive influence of this strategic approach on inhibiting the growth of both cancer cell lines is unmistakably evident. The IC_50_ values against the two tested cell lines for series **14a**–**d** were consistently lower than their counterparts from the first series (**11a**, **11d**, **11g** and **11j**). The only exception was for HepG2 with compound **14b**. Although its IC_50_ value resided in the submicromolar level (0.98 µM), it marginally surpassed its counterpart **11d** (IC_50_ = 0.73 µM).

When assessing the impact of *N*-substitution with a methyl or benzyl group, it becomes apparent that their effects on enhancing efficacy are closely comparable. For example, introducing *N*-methyl to compound **11g** (IC_50_ = 0.77 µM) yielded compound **14c** (IC_50_ = 0.2 µM), demonstrating nearly a 4-fold increase in inhibitory activity against PANC1. Similarly, incorporating *N*-benzyl into **11j** (IC_50_ = 1.68 µM) generated **14d** (IC_50_ = 0.42 µM), displaying the same magnitude of efficacy increase against PANC1.

In addition, we evaluated the lethal effects of the new indolin-2-one-triazoles **11e**, **11d**, **11g**, **11k** and **14c** on the non-tumorigenic Vero cell line to determine their specificity toward the tested cancer cells. Remarkably, the indolin-2-one-triazoles that were studied showed low toxicity toward the normal Vero cells, with IC_50_ values of 8.35 ± 0.62, 11.74 ± 0.93, 10.65 ± 0.71, 13.22 ± 1.09 and 7.92 ± 0.48, respectively. This indicates that they have good selectivity indexes and a safe profile (Table 2).

#### 2.2.2. VEGFR-2 Inhibitory Activities

For further exploration of the synthesized compounds’ mechanism of action, a VEGFR-2 inhibition assay was conducted for compounds **11e**, **11d**, **11g**, **11k** and **14c** using Sorafenib as the reference compound. Appealingly, compound **11d**, bearing the electron-withdrawing group 5-Br, evinced greater VEGFR-2 inhibition over Sorafenib, where it expressed IC_50_ of 16.3 nM, while Sorafenib’s IC_50_ was 29.7 nM. Moreover, compounds **11e**, **11k** and **14c** demonstrated anti-VEGFR-2 activity at a level with Sorafenib where their IC_50_ ranged from 41.3 to 53.8 nM. Noteworthy, the replacement of the electron-withdrawing group 5-Br in compound **11d** with the electron-donating group 5-OCH_3_ in compounds **11e** and **11k** resulted in a decrease in the anti-VEGFR-2 activity. Eventually, compound **11g** showed fair VEGFR-2 inhibition with an IC_50_ of 119.6 nM, Table 3. Impressively, compound **11d** manifests superior activity over compound **III**, which is considered a structurally similar compound to ours (VEGFR-2 IC_50_ = 26.3 nM). Since compound **III** was previously reported to have better VEGFR-2 inhibitory activity over Sunitinib (IC_50_ = 39.7 nM), this strongly imparts great confidence toward compound **11d** to effectively halt VEGFR-2 activity. In conclusion, the tested compounds are considered potent VEGFR-2 inhibitors; however, other kinases can be targeted through these compounds. Kinase activity profiling can be attempted in our future work to confirm the compounds’ selectivity versus VEGFR-2.

Attempting to deduce SAR from the data available in Table 3, it can be stated that the presence of Br at position 5 in the indolin-2-one moiety (**11d**) enhanced the efficacy against VEGFR-2 by approximately 3-fold compared to the presence of OCH_3_ at the same position (**11e**). Clear observations from the evaluated chlorinated analogs revealed that the presence of substitution at position 5 (**11k**) or on *N*1 (**14c**) of the indolin-2-one motif increased activity by approximately 3 and 2.25 times, respectively, compared to when the indolin-2-one ring was unsubstituted (**11g**).

### 2.3. In Silico ADME Study

**Figure 3:**
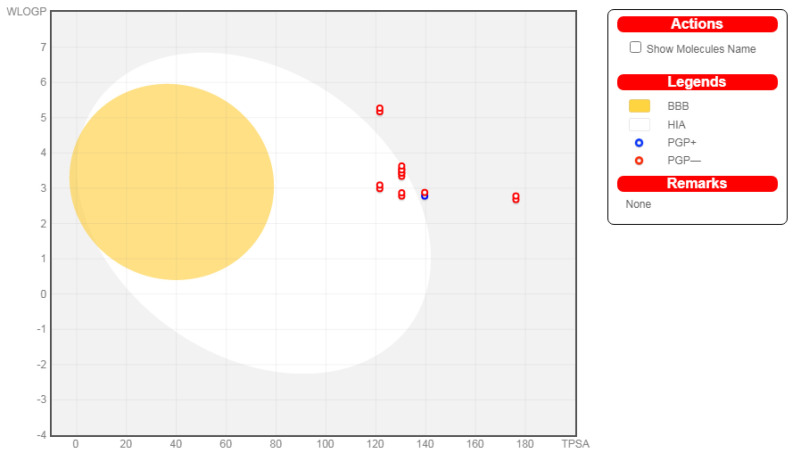
BOILED-Egg plot of the synthesized compounds.

In silico investigation of the pharmacokinetic properties together with the drug-likeness of the synthesized compounds was performed using SwissADME [26]. Fortunately, the achieved results demonstrate the drug-likeness of the active compounds where all the compounds were established to obey Veber rules with the exception of compounds **11f** and **11l** [27]. However, a maximum of two violations of the Lipinski rule was observed with some compounds. Being an important concern towards drug–drug interactions, which results in undesirable adverse reactions, prediction of the inhibition of different cytochrome P isoforms was demonstrated in Table 4. Moreover, the ADME properties, including blood–brain barrier (BBB) permeation, human gastrointestinal absorption (HIA), as well as being P-glycoprotein substrate or not, are presented in the BOILED-Egg model (Figure 3). Compounds **11a**–**11d**, **11g**–**11i**, **14a** and **14c** revealed high GI absorption. Meanwhile, all the compounds are suggested to not be permeable to the BBB, indicating no CNS side effects. Also, they are not expected to be substrates for P-glycoprotein (with the exception of compound **11e**). 

### 2.4. Molecular Modelling 

#### 2.4.1. Molecular Docking

**Figure 4:**
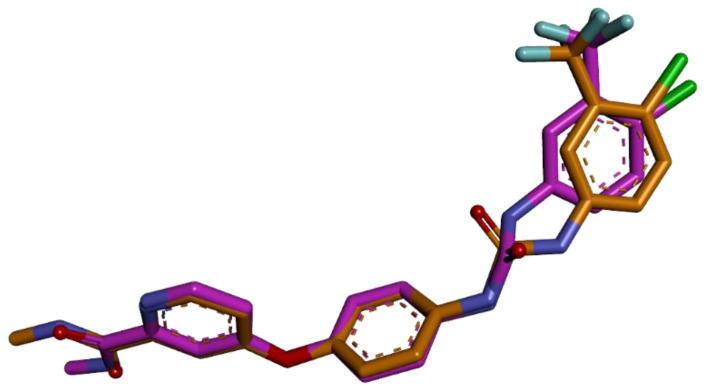
Superimposition of the co-crystallized ligand. Sorafenib (orange) and the docked pose (violet) of VEGFR-2.

**Figure 5:**
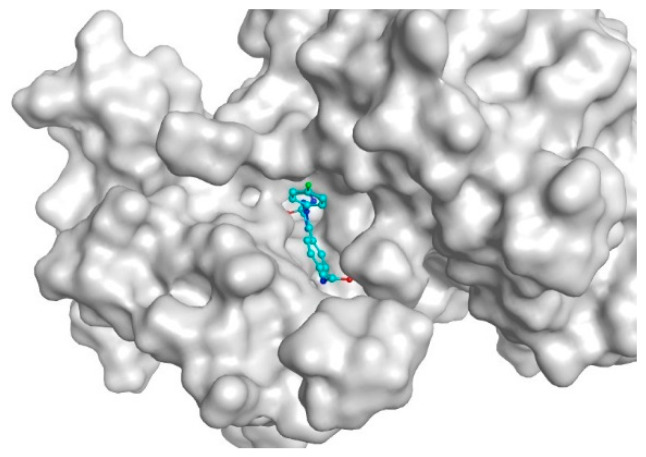
Binding of **11d** with VEGFR-2, showing the whole protein and **11d** located in the active site.

To evaluate the binding modes of the synthesized indolin-2-ones and establish correlations between their structural attributes and inhibitory efficacy, a molecular docking analysis was conducted on VEGFR-2, with the PDB ID 4ASD as the reference [28]. The validation of the molecular docking protocol commenced with the re-docking of Sorafenib, the co-crystallized ligand, at the ATP active site. The minimal root mean square deviation (RMSD) values of 1.15 Å between the co-crystallized ligand and the docked pose signify a nearly identical superimposition, affirming the practicality of the employed configuration for the proposed docking experiment, Figure 4. Also, Figure 5 shows **11d** located in the VEGFR-2 active site.

Strikingly, derivative **11d** was recognized as the most captivating VEGFR-2 inhibitor in this study. Hence, it was selected for docking into the VEGFR-2 active site. It is noteworthy to mention that compound **11d** engaged in interactions characterized by the establishment of four hydrogen bonds. Specifically, three of these bonds originated from the triazole ring’s N4, establishing connections with crucial residues (Lys868, Glu885 and Asp1046), while the fourth hydrogen bond formed between the terminal acetamide moiety’s C=O and Arg1027. Additionally, the bromine atom exhibited participation in a halogen bonding interaction with Leu840. 

**Figure 6:**
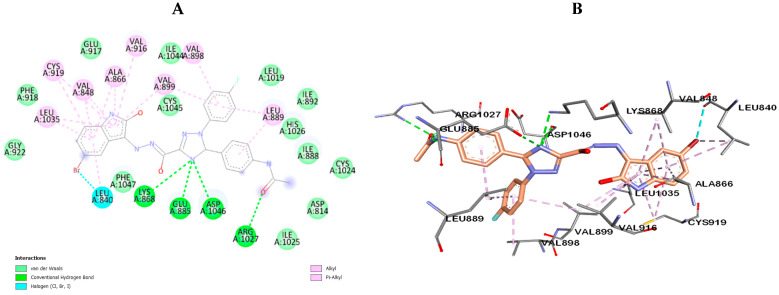
Docking representation of **11d** within the active site of VEGFR-2 (**A**) 2D schematic presentation and (**B**) active site display.

Regarding hydrophobic interactions, an observable pattern emerged wherein all aromatic components of **11d**, including the indolin-2-one motif and the two phenyl rings, interacted through π—alkyl interactions with multiple residues within the active site, namely Leu840, Val848, Ala866, Leu889, Val898, Val899, Val916, Cys919 and Leu1035, Figure 6. The strong interactions observed within the ATP binding site explain the superior activity observed for compound **11d** over Sorafenib. 

On the other hand, Table 5 presents the docking score of compound **11d**, along with other compounds (**11e**, **11g**, **11k** and **14c**) that were tested for their VEGFR-2 inhibitory action. Compound **11d** exhibited a score (S = −9.6 kcal/mol) that was comparable to Sorafenib (S = −9.5 kcal/mol) and higher scores than **11e**, **11g**, **11k** and **14c**, which had scores of −8.3, −7.1, −8.5 and 18.0 kcal/mol, respectively (Table 5). These results could justify **11d**’s superior inhibitory activity.

#### 2.4.2. Molecular Dynamics

The outcomes obtained from both biological and docking investigations established compound **11d** as a promising anti-cancer agent due to its potent inhibition of VEGFR-2. This prompted us to delve deeper into its properties through molecular dynamic simulations (MDSs) for more in silico insights. MDSs are preferred for their precision in assessing the stability of a protein–ligand complex compared to other computational methods. Taking advantage of this, we simulated the binding poses of **11d** with VEGFR-2, retrieved from the docking stage, for 100 ns. To facilitate and validate comparison, the Apo form of the target and the crystal coordinates of VEGFR-2 bound to Sorafenib were included in the simulation.

**Figure 7:**
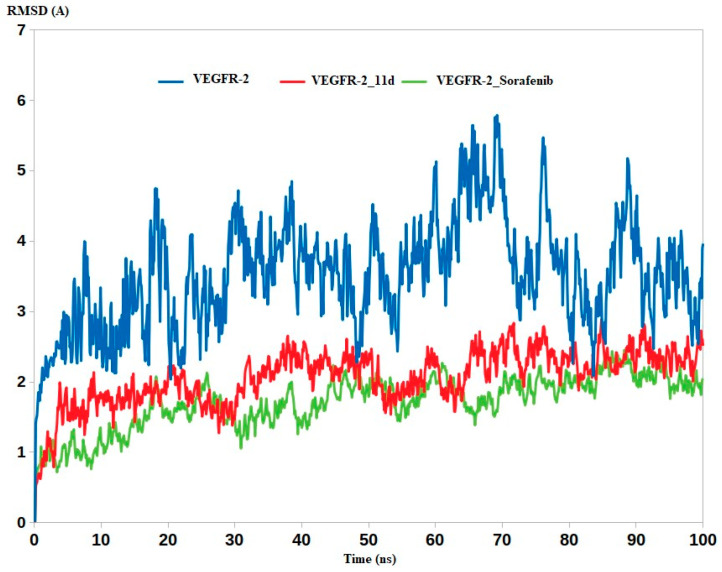
RMSD analysis for the MDSs against VEGFR-2 enzyme for 100 ns.

**Figure 8:**
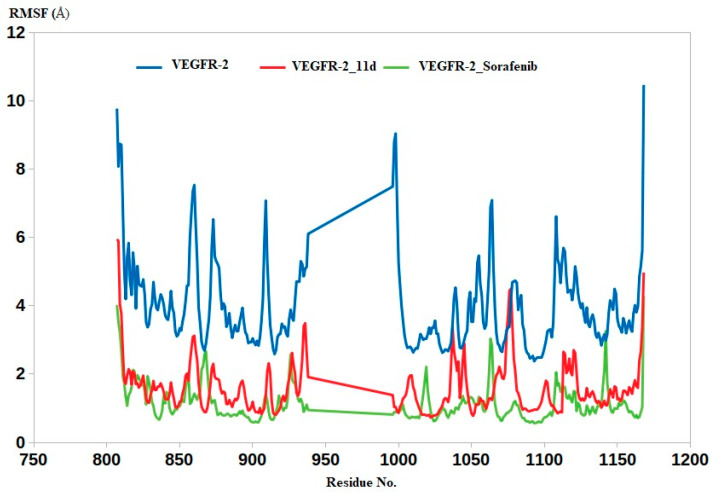
RMSF analysis for the MDSs against VEGFR-2 enzyme for 100 ns.

Figure 7 illustrates the considerable dynamicity of the free VEGFR-2 protein, indicative of its role as a primary oncogenic protein. This was evident in the RMSD calculations, where unbound VEGFR-2 displayed RMSD values of 4.2 Å. Notably, compound **11d** exhibited a remarkable capability to restrict the dynamic behavior of VEGFR-2, as evidenced by lower RMSD values of approximately 1.8 Å compared to Sorafenib’s 1.6 Å. The RMSF values mirrored the RMSD findings, with Apo proteins’ residues experiencing average fluctuations of 4.1 Å for VEGFR-2 (Figure 8). The binding of **11d** and Sorafenib induced significant stability in VEGFR-2 residues, reducing their fluctuation to average RMSFs of 1.5 and 1.2 Å, respectively. In summary, both RMSD and RMSF calculations converge on the conclusion that compound **11d** effectively inhibits VEGFR-2 by forming robust and stable interactions with the enzyme’s active sites.

## 3. Materials and Methods

### 3.1. Chemistry 

Two devices used for acquiring NMR spectra belong to Bruker (Billerica, MA, USA), namely the Avance III Smart Probe Spectrometers. One operates at 400 MHz (400 MHz ^1^H and 101 MHz ^13^C NMR) and the other at 700 MHz (700 MHz ^1^H and 176 MHz ^13^C NMR). Additionally, a JOEL instrument (JEOL, Tokyo, Japan), specifically the ECA-500 II spectrometer (500 MHz ^1^H and 126 MHz ^13^C NMR), was employed for some compounds. The chemical shift was recorded in parts per million (*δ*). In all samples, deuterated dimethylsulfoxide (DMSO-*d6*) was utilized as a solvent. The coupling constants (*J*) were measured in Hertz (Hz). The Bruker MicroTOF spectrometer was used to obtain HRMS. A Stuart melting point device was used to determine uncorrected melting points. All reaction chemicals and solvents were consumed without further purification after being purchased from commercial vendors.

The *N*-alkylation of indolin-2-one derivatives **10a** and **10d** to give *N*-methyl and *N*-benzyl indolin-2-one derivatives **13a** and **13b**, respectively, was performed according to a previously described methodology [29,30].

#### 3.1.1. Synthesis of 4-Nitrohippuric Acid (**3**)

A solution of NaOH (2.7 N) in water (15 mL) was prepared, and then glycine (1.52 g, 20.31 mmol) was added. At room temperature, 4-nitrobenzoyl chloride powder (3.76 g, 20.31 mmol) was added portion by portion before the reaction mixture was left stirring for only 1 h. After the designated duration, HCl was added dropwise to acidify the medium in addition to ice cubes. A white precipitate was collected through filtration, subjected to washing with water (3 × 5 mL) and petroleum ether (2 × 2 mL), and encouraged to dry using reduced pressure to yield 4-nitrohippuric acid **3** [31]. Yield 63%; melt. pt. 134–135 °C (reported melt. pt. 131–132 °C [32]). 

#### 3.1.2. Synthesis of 4-Aminohippuric Acid (**4**)

A solution of 4-nitrohippuric acid **3** (2.87 g, 12.8 mmol) in anhydrous methanol (40 mL) was prepared. Under N_2_ atmosphere, Pd/C (10 wt% on activated carbon, 270 mg) was added. Reduction happened when temperature and H_2_ pressure were adjusted at 25 °C and 1 bar, respectively. Subsequently, the reaction mixture was filtered off using a pad of celite, followed by the removal of methanol utilizing rotary evaporation to give 4-aminohippuric acid **4** as a white solid [33]. Yield 74%; melt. pt. 197–198 °C (reported melt. pt. 199 °C [34]). 

#### 3.1.3. Synthesis of *N*-{4-[(4-[2-Arylhydrazin-1-ylidene]-5-oxo-4,5-dihydro-1,3-oxazol-2-yl]phenyl}acetamides (**8a**–**b**)

4-Aminohippuric acid **4** (0.92 g, 4.73 mmol) was added to acetic anhydride (5 mL), then the temperature was elevated to reach 75 °C. The suspension was left for 40 min until it transformed into a yellowish-orange solution (solution A), which indicates the production of **5**. Thereafter, the solution was allowed to cool to ambient temperature. In an ice bath 0–5 °C, 2 mL of 5N HCl was added to an appropriate aniline derivative **6a**–**b** (3.67 mmol), then the mixture was left stirring for 20 min before 1 mL of an aqueous solution of NaNO_2_ (0.33 g, 4.73 mmol) was added gradually to form the diazonium salts of aniline derivatives **7a**–**b**. Fifteen minutes later, anhydrous sodium acetate (0.54 g, 6.59 mmol) was introduced to the reaction vessel (solution B). Thereafter, solution A was incrementally introduced to solution B and left to stir for 2 h at a temperature maintained between 0–5 °C. Flake ice was added to the mixture and the resulting product filtered and underwent thorough washing with distilled water (4 × 5 mL), methanol (2 × 3 mL) and petroleum ether (3 × 3 mL), followed by drying at 80 °C to afford acetamido-tethered hydrazones **8a**–**b** (yield 73–80.3%) [21]. 

##### *N*-{4-[(4-[2-(4-Fluorophenyl)hydrazin-1-ylidene]-5-oxo-4,5-dihydro-1,3-oxazol-2-yl]phenyl}acetamide (**8a**)

Orange powder (yield 80.3%); melt. pt. 269–271 °C; ^1^H NMR (500 MHz, DMSO-d_6_) δ ppm: 2.10 (s, 3H, Methyl), 7.20 (t, 2H, Ar. Proton, J = 8.8 Hz), 7.48 (dd, 2H, Ar. Proton, J = 9.2, 4.4 Hz), 7.82 (d, 2H, Ar. Proton, J = 8.4 Hz), 8.03 (d, 2H, Ar. Proton, J = 8.8 Hz), 10.42 (s, 1H, Acetamide NH), 11.68 (s, 1H, =N-NH); ^13^C NMR (126 MHz, DMSO-d_6_) δ ppm: 24.24 (Methyl), 115.99 (d, ^2^J_CF_ = 22.8 Hz), 116.29 (d, ^3^J_CF_ = 7.9 Hz), 118.88, 119.11, 129.06, 131.96, 139.46, 144.11, 158.29 (d, ^1^J_CF_ = 239.2 Hz), 161.08, 161.32 (Azalactone Carbonyl), 169.13 (Acetamide Carbonyl); anal. calcd. for C_17_H_13_FN_4_O_3_ (340.31 g/mol): C, 60.00; H, 3.85; N, 16.46; practical C, 59.93; H, 3.86; N, 16.48.

##### *N*-{4-[(4-[2-(4-Chlorophenyl)hydrazin-1-ylidene]-5-oxo-4,5-dihydro-1,3-oxazol-2-yl]phenyl}acetamide (**8b**)

Orange powder (yield 73%); melt. pt. > 300 °C; ^1^H NMR (500 MHz, DMSO-d_6_) δ ppm: 2.10 (s, 3H, Methyl), 7.39 (d, 2H, Ar. Proton, J = 8.8 Hz), 7.47 (d, 2H, Ar. Proton, J = 9.6 Hz), 7.82 (d, 2H, Ar. Proton, J = 8.8 Hz), 8.03 (d, 2H, Ar. Proton, J = 8.8 Hz), 10.42 (s, 1H, Acetamide NH), 11.70 (s, 1H, =N-NH); ^13^C NMR (126 MHz, DMSO-d_6_) δ ppm: 24.23 (Methyl), 116.31, 118.87, 119.00, 126.49, 129.17, 130.26, 132.59, 141.87, 144.22, 161.25, 161.57 (Azalactone Carbonyl), 169.12 (Acetamide Carbonyl); anal. calcd. for C_17_H_13_ClN_4_O_3_ (356.77 g/mol): C, 57.23; H, 3.67; N, 15.70; practical C, 57.33; H, 3.65; N, 15.64.

#### 3.1.4. Synthesis of *N*-{4-[1-Aryl-3-(hydrazinecarbonyl)-1*H*-1,2,4-triazol-5-yl]phenyl}acetamide (**9a**–**b**)

A pre-heated suspension of **8a**–**b** (2.67 mmol) in ethanol (4 mL) was subjected to an excess of hydrazine hydrate 95% (0.4 g, 8 mmol). The resulting mixture underwent reflux for 1 h before being cooled to ambient temperature and put into flake ice-containing water. The generated solid was filtered, washed sequentially with water (4 × 5 mL) and petroleum ether (3 × 2 mL) and crystallized from isopropanol to obtain acetamido-based hydrazides **9a**–**b** in 59–64.6% yield [21]. 

##### *N*-{4-[1-(4-Fluorophenyl)-3-(hydrazinecarbonyl)-1*H*-1,2,4-triazol-5-yl]phenyl}acetamide (**9a**)

Light brown crystals (yield 59%); melt. pt. 115–118 °C; ^1^H NMR (700 MHz, DMSO-d_6_) δ ppm: 2.06, 2.07 (2s, 3H, Methyl), 4.60 (s, 2H, NH_2_), 7.38–7.41 (m, 3.85H, Ar. Proton), 7.45 (dd, 0.15H, Ar. Proton, J = 6.9, 1.7 Hz), 7.52–7.55 (m, 1.85H, Ar. Proton), 7.59–7.62 (m, 1.8H, Ar. Proton), 7.65 (d, 0.15H, Ar. Proton, J =8.6 Hz), 7.72–7.74 (m, 0.2H, Ar. Proton), 9.89, 9.98 (2s, 1H, Hydrazide NH), 10.18, 10.21 (2s, 1H, Acetamide NH); ^13^C NMR (176 MHz, DMSO-d_6_) δ ppm: (24.53, 24.55 (Methyl)), 117.02 (d, ^2^J_CF_ = 23.2 Hz), 119.01, 119.12, 121.28, 121.54, 125.45, 127.01, 128.76 (d, ^3^J_CF_ = 9.1 Hz), 129.93, 130.25, 134.47 (d, ^4^J_CF_ = 3.0 Hz), 141.59, 141.90, 142.77, 147.70, 154.80, 155.22, 156.17, 156.76, (158.22, 158.52 (Hydrazide Carbonyl)), 162.52 (d, ^1^J_CF_ = 247.4 Hz), [169.29, 169.34 (Acetamide Carbonyl)]; anal. calcd. for C_17_H_15_FN_6_O_2_ (354.35 g/mol): C, 57.62; H, 4.27; N, 23.72; practical C, 57.61; H, 4.25; N, 23.74; HRMS (ESI) for C_17_H_16_FN_6_O_2_, calcd 355.1313, found 355.1318 [M+H]^+^.

##### *N*-{4-[1-(4-Chlorophenyl)-3-(hydrazinecarbonyl)-1*H*-1,2,4-triazol-5-yl]phenyl}acetamide (**9b**)

Beige crystals (yield 64.6%); melt. pt. 140–141 °C; ^1^H NMR (400 MHz, DMSO-d_6_) δ ppm: 2.06 (2s, 3H, Methyl), 4.59 (s, 2H, NH_2_), 7.40–7.45 (m, 2H, Ar. Proton), 7.49 (dt, 2H, Ar. Proton, J = 9.3, 2.6 Hz), 7.60–7.64 (m, 4H, Ar. Proton), 9.89 (s, 1H, Hydrazide NH), 10.17 (s, 1H, Acetamide NH); ^13^C NMR (101 MHz, DMSO-d_6_) δ ppm: 24.53 (Methyl), 119.09, 121.47, 127.95, 129.99, 130.07, 134.45, 136.85, 141.68, 154.80, 156.33, 158.48 (Hydrazide Carbonyl), 169.31 (Acetamide Carbonyl); anal. calcd. for C_17_H_15_ClN_6_O_2_ (370.80 g/mol): C, 55.07; H, 4.08; N, 22.67; practical C, 55.13; H, 4.06; N, 22.70.

#### 3.1.5. Synthesis of *N*-[4-(1-Aryl-3-{*N*′-[2-oxo-2,3-dihydro-1H-indol-3-ylidene]hydrazinecarbonyl}-1*H*-1,2,4-triazol-5-yl)phenyl]acetamides (**11a**–**l** and **14a**–**d**)

Indolin-2-one derivatives **10a**–**f** or **13a**–**b** (0.2 mmol) were added to a pre-heated solution of a selected hydrazide derivative **9a**–**b** (0.2 mmol) in absolute ethanol (8 mL), then a catalytic quantity of glacial acetic acid (5 drops) was included, and the resulting mixture was then subjected to reflux for a duration of 2 h. After the reaction had run its course, the formed solid was collected via filtration under reduced pressure and rinsed with water (3 × 3 mL), methanol (2 × 2 mL) and ethyl ether (2 × 2 mL). Finally, the precipitate was dried at 100 °C to yield hybrids **11a**–**l** and **14a**–**d** in 38.9–72.7% yield.

##### *N*-{4-[1-(4-Fluorophenyl)-3-{*N*′-[2-oxo-2,3-dihydro-1*H*-indol-3-ylidene]hydrazinecarbonyl}-1*H*-1,2,4-triazol-5-yl]phenyl}acetamide (**11a**)

Yellow powder (yield 39.1%); melt. pt. > 300 °C; ^1^H NMR (700 MHz, DMSO-d_6_) δ ppm: 2.07, 2.08 (2s, 3H, Methyl), 6.98 (d, 1H, Ar. Proton, J = 7.8 Hz), 7.14 (t, 1H, Ar. Proton, J = 7.7 Hz), 7.38–7.50 (m, 4.7H, Ar. Proton), 7.59–7.70 (m, 4.7H, Ar. Proton), 7.80 (d, 0.3H, Ar. Proton, J = 8.9 Hz), 8.42 (d, 0.3H, Ar. Proton, J = 9.1 Hz), 10.21, 10.25 (2s, 1H, Acetamide NH), 11.29 (s, 1H, Indolin-2-one NH), 14.29, 14.32 (2s, 1H, Hydrazide NH); ^13^C NMR (176 MHz, DMSO-d_6_) δ ppm: 24.55 (Methyl), 111.72, 117.10, 117.23, 119.07, 120.24, 121.10, 121.68, 123.22, 127.37, 128.97, 129.02, 130.02, 130.33, 132.59, 134.33, 139.33, 141.85, 143.31, 154.89, 155.66 (Hydrazide Carbonyl), 163.07 (Indolin-2-one Carbonyl), 169.33 (Acetamide Carbonyl); anal. calcd. for C_25_H_18_FN_7_O_3_ (483.46 g/mol): C, 62.11; H, 3.75; N, 20.28; practical C, 62.13; H, 3.77; N, 20.21; HRMS (ESI) for C_25_H_19_FN_7_O_3_, calcd 484.1528, found 484.1532 [M+H]^+^, and for C_25_H_18_FN_7_NaO_3_, calcd 506.1347, found 506.1352 [M+Na]^+^.

##### *N*-[4-(3-{*N*′-[5-Fluoro-2-oxo-2,3-dihydro-1*H*-indol-3-ylidene]hydrazinecarbonyl}-1-(4-fluorophenyl)-1*H*-1,2,4-triazol-5-yl)phenyl]acetamide (**11b**)

Yellow powder (yield 56.2%); melt. pt. > 300 °C; ^1^H NMR (700 MHz, DMSO-d_6_) δ ppm: 2.07 (s, 3H, Methyl), 6.98 (s, 1H, Ar. Proton), 7.27 (s, 1H, Ar. Proton), 7.35–7.55 (m, 5H, Ar. Proton), 7.56–7.72 (m, 4H, Ar. Proton), 7.81 (d, 0.5H, Ar. Proton, J = 6.9 Hz), 8.42 (d, 0.5H, Ar. Proton, J = 5.8 Hz), 10.22, 10.25 (2s, 1H, Acetamide NH), 11.31 (s, 1H, Indolin-2-one NH), 14.30, 14.32 (2s, 1H, Hydrazide NH); anal. calcd. for C_25_H_17_F2N_7_O_3_ (501.45 g/mol): C, 59.88; H, 3.42; N, 19.55; practical C, 60.03; H, 3.41; N, 19.47; HRMS (ESI) for C_25_H_18_F_2_N_7_O_3_, calcd 502.1434, found 502.1437 [M+H]^+^, and for C_25_H_17_F2N_7_NaO_3_, calcd 524.1253, found 524.1256 [M+Na]^+^.

##### *N*-[4-(3-{*N*′-[5-Chloro-2-oxo-2,3-dihydro-1*H*-indol-3-ylidene]hydrazinecarbonyl}-1-(4-fluorophenyl)-1H-1,2,4-triazol-5-yl)phenyl]acetamide (**11c**)

Yellow powder (yield 48.9%); melt. pt. > 300 °C; ^1^H NMR (500 MHz, DMSO-d_6_) δ ppm: 2.05, 2.06 (2s, 3H, Methyl), 6.96 (d, 1H, Ar. Proton, J = 8.0 Hz), 7.42–46 (m, 2H, Ar. Proton), 7.48–7.52 (m, 3H, Ar. Proton), 7.62–7.66 (m, 4H, Ar. Proton), 7.98 (d, 1H, Ar. Proton, J = 1.6 Hz), 10.20, 10.23 (2s, 1H, Acetamide NH), 11.03, 11.39 (2s, 1H, Indolin-2-one NH), 11.88, 14.24 (2s, 1H, Hydrazide NH); ^13^C NMR (126 MHz, DMSO-d_6_) δ ppm: 24.11 (Methyl), 112.54, 116.67, 116.78, 116.86, 118.54, 120.45, 125.13, 125.65, 125.85, 126.77, 128.33, 128.41, 129.44, 129.77, 132.55, 133.87, 141.53, 142.91, 154.64, 154.73 (Hydrazide Carbonyl), 161.33, 163.30 (Indolin-2-one Carbonyl), 164.14, 168.84 (Acetamide Carbonyl); anal. calcd. for C_25_H_17_ClFN_7_O_3_ (517.91 g/mol): C, 57.98; H, 3.31; N, 18.93; practical C, 57.86; H, 3.32; N, 18.99.

##### *N*-[4-(3-{*N*′-[5-Bromo-2-oxo-2,3-dihydro-1*H*-indol-3-ylidene]hydrazinecarbonyl}-1-(4-fluorophenyl)-1*H*-1,2,4-triazol-5-yl)phenyl]acetamide (**11d**)

Yellow powder (yield 44.3%); melt. pt. > 300 °C; ^1^H NMR (700 MHz, DMSO-d_6_) δ ppm: 2.06, 2.07 (2s, 3H, Methyl), 6.92 (d, 0.3H, Ar. Proton, J = 8.2 Hz), 6.93 (d, 0.7H, Ar. Proton, J = 8.2 Hz), 7.40–7.70 (m, 9H, Ar. Proton), 8.11 (s, 0.8H, Ar. Proton), 8.43 (d, 0.2H, Ar. Proton, J = 8.9 Hz), 10.20, 10.23 (2s, 1H, Acetamide NH), 11.04, 11.39 (2s, 1H, Indolin-2-one NH), 11.83, 14.21 (2s, 1H, Hydrazide NH); ^13^C NMR (176 MHz, DMSO-d_6_) δ ppm: (24.55, 24.56 (Methyl)), 113.50, 113.68, 113.77, 114.87, 117.16 (d, ^2^J_CF_ = 23.2 Hz), 117.23 (d, ^2^J_CF_ = 23.3 Hz), 117.77, 119.06, 119.15, 120.92, 121.03, 122.36, 123.89, 125.59, 127.22, 128.81 (d, ^3^J_CF_ = 9.1 Hz), 128.93, 128.99, 129.93, 129.99, 130.26, 134.30 (d, ^4^J_CF_ = 2.99 Hz), 134.36 (d, ^4^J_CF_ = 2.9 Hz), 134.69, 135.81, 138.23, 141.80, 141.87, 141.98, 142.32, 143.71, 154.74, 155.10, 155.20, (155.65, 155.75 (Hydrazide Carbonyl)), (162.06, 162.70 (Indolin-2-one Carbonyl)), 162.79 (d ^1^J_CF_ = 247.8 Hz), 164.50, (169.32, 169.34 (Acetamide Carbonyl)); anal. calcd. for C_25_H_17_BrFN_7_O_3_ (562.36 g/mol): C, 53.40; H, 3.05; N, 17.44; practical C, 53.37; H, 3.06; N, 17.48; HRMS (ESI) for C_25_H_18_BrFN_7_O_3_, calcd 562.0633, found 562.0636 [M+H]^+^, and for C_25_H_17_BrFN_7_NaO_3_, calcd 584.0452, found 584.0455 [M+Na]^+^.

##### *N*-{4-[1-(4-Fluorophenyl)-3-{*N*′-[5-methoxy-2-oxo-2,3-dihydro-1*H*-indol-3-ylidene]hydrazinecarbonyl}-1*H*-1,2,4-triazol-5-yl]phenyl}acetamide (**11e**)

Orange powder (yield 61.4%); melt. pt. > 300 °C; ^1^H NMR (700 MHz, DMSO-d_6_) δ ppm: 2.07, 2.08 (2s, 3H, Methyl), 3.72, 3.79 (2s, 3H, Methoxy), 6.88 (d, 0.5H, Ar. Proton, J = 8.5 Hz), 6.89 (d, 0.5H, Ar. Proton, J = 8.5 Hz), 6.99 (dd, 0.4H, Ar. Proton, J = 8.5, 2.6 Hz), 7.06 (dd, 0.6H, Ar. Proton, J = 8.5, 2.4 Hz), 7.17 (d, 0.35H, Ar. Proton, J = 2.5 Hz), 7.38–7.52 (m, 4H, Ar. Proton), 7.55 (d, 0.65H, Ar. Proton, J = 2.1 Hz), 7.58–7.71 (m, 4H, Ar. Proton), 10.20, 10.21 (2s, 1H, Acetamide NH), 10.71, 11.09 (2s, 1H, Indolin-2-one NH), 11.72, 14.31 (2s, 1H, Hydrazide NH); ^13^C NMR (176 MHz, DMSO-d_6_) δ ppm: (24.55, 24.56 (Methyl)), (55.98, 56.10 (Methoxy)), 106.35, 111.91, 112.29, 112.60, 116.25, 117.15 (d, ^2^J_CF_ = 23.2 Hz), 117.18 (d, ^2^J_CF_ = 23.4 Hz), 119.05, 119.44, 120.91, 121.01, 121.08, 125.54, 127.34, 128.82 (d, ^3^J_CF_ = 9.5 Hz), 128.97 (d, ^3^J_CF_ = 9.8 Hz), 129.96, 130.00, 134.29, 136.96, 138.34, 139.60, 141.94, 142.95, 154.86, 154.94, 155.16, 155.39, 155.63, (155.79, 155.89 (Hydrazide Carbonyl)), 162.76 (d, ^1^J_CF_ = 248.0 Hz), (163.16, 164.94 (Indolin-2-one Carbonyl)), (169.33, 169.35 (Acetamide Carbonyl)); anal. calcd. for C_26_H_20_FN_7_O_4_ (513.49 g/mol): C, 60.82; H, 3.93; N, 19.09; practical C, 60.81; H, 3.91; N, 19.07; HRMS (ESI) for C_26_H_21_FN_7_O_4_, calcd 514.1634, found 514.1639 [M+H]^+^, and for C_26_H_20_FN_7_NaO_4_, calcd 536.1453, found 536.1458 [M+Na]^+^.

##### *N*-{4-[1-(4-Fluorophenyl)-3-{*N*′-[5-nitro-2-oxo-2,3-dihydro-1*H*-indol-3-ylidene]hydrazinecarbonyl}-1*H*-1,2,4-triazol-5-yl]phenyl}acetamide (**11f**)

Greenish-yellow powder (yield 46.6%); melt. pt. > 300 °C; ^1^H NMR (700 MHz, DMSO-d_6_) δ ppm: 2.07, 2.08 (2s, 3H, Methyl), 7.14 (d, 1H, Ar. Proton, J = 8.4 Hz), 7.47 (t, 2H, Ar. Proton, J = 8.4 Hz), 7.58 (d, 1.6H, Ar. Proton, J = 8.2 Hz), 7.61 (d, 0.4H, Ar. Proton, J = 8.1 Hz), 7.64–7.72 (m, 3.8H, Ar. Proton), 7.88 (d, 0.2H, Ar. Proton, J = 8.3 Hz), 8.38 (d, 0.85H, Ar. Proton, J = 8.4 Hz), 8.43 (d, 0.15H, Ar. Proton, J = 8.2 Hz), 8.84 (s, 1H, Ar. Proton), 10.23, 10.26 (2s, 1H, Acetamide NH), 11.61 (s, 1H, Indolin-2-one NH), 12.11 (s, 1H, Hydrazide NH); ^13^C NMR (176 MHz, DMSO-d_6_) δ ppm: 24.55 (Methyl), 111.63, 115.94, 117.24 (d, ^2^J_CF_ = 23.3 Hz), 119.03, 120.86, 122.08, 125.57, 128.89 (d, ^3^J_CF_ = 8.9 Hz), 129.56, 130.00, 134.41 (d, ^4^J_CF_ = 2.8 Hz), 142.00, 142.33, 149.94, 155.28 (Hydrazide Carbonyl), 162.11, 163.12 (Indolin-2-one Carbonyl), 165.24, 169.36 (Acetamide Carbonyl); anal. calcd. for C_25_H_17_FN_8_O_5_ (528.46 g/mol): C, 56.82; H, 3.24; N, 21.20; practical C, 56.95; H, 3.25; N, 21.12; HRMS (ESI) for C_25_H_18_FN_8_O_5_, calcd 529.1379, found 529.1384 [M+H]^+^, and for C_25_H_17_FN_8_NaO_5_, calcd 551.1198, found 551.1200 [M+Na]^+^.

##### *N*-{4-[1-(4-Chlorophenyl)-3-{*N*′-[2-oxo-2,3-dihydro-1*H*-indol-3-ylidene]hydrazinecarbonyl}-1*H*-1,2,4-triazol-5-yl]phenyl}acetamide (**11g**)

Yellow powder (yield 69.1%); melt. pt. > 300 °C; ^1^H NMR (400 MHz, DMSO-d_6_) δ ppm: 2.07, 2.08 (2s, 3H, Methyl), 6.97 (d, 1H, Ar. Proton, J = 7.8 Hz), 7.11–7.18 (m, 1H, Ar. Proton), 7.40–7.50 (m, 3H, Ar. Proton), 7.56–7.59 (m, 2H, Ar. Proton), 7.63–7.69 (m, 5H, Ar. Proton), 10.20 (s, 1H, Acetamide NH), 10.90, 11.27 (2s, 1H, Indolin-2-one NH), 11.70, 14.29 (2s, 1H, Hydrazide NH); ^13^C NMR (101MHz, DMSO-d_6_) δ ppm: 24.49 (Methyl), 111.69, 119.10, 120.14, 121.01, 121.64, 123.18, 128.07, 128.15, 130.01, 130.17, 132.54, 134.88, 136.67, 139.28, 141.84, 143.22, 154.99, 155.51 (Hydrazide Carbonyl), 163.00 (Indolin-2-one Carbonyl), 169.44 (Acetamide Carbonyl); anal. calcd. for C_25_H_18_ClN_7_O_3_ (499.92 g/mol): C, 60.07; H, 3.63; N, 19.61; practical C, 60.11; H, 3.64; N, 19.55.

##### *N*-{4-[1-(4-Chlorophenyl)-3-{*N*′-[5-fluoro-2-oxo-2,3-dihydro-1*H*-indol-3-ylidene]hydrazinecarbonyl}-1*H*-1,2,4-triazol-5-yl]phenyl}acetamide (**11h**)

Yellow powder (yield 38.9%); m.p. > 300 °C; ^1^H NMR (400 MHz, DMSO-d_6_) δ ppm: 2.07 (s, 3H, CH_3_), 6.97 (dd, 1H, Ar-H, J = 8.6, 4.2 Hz), 7.26 (td, 1H, Ar-H, J = 9.4, 2.6 Hz), 7.41–7.50 (m, 3H, Ar-H), 7.53–7.61 (m, 2H, Ar-H), 7.62–7.70 (m, 4H, Ar-H), 10.20 (s, 1H, NH-Acetamide), 11.29 (s, 1H, NH-Isatin), 14.29 (s, 1H, NH-Hydrazide); ^13^C NMR (101 MHz, DMSO-d_6_) δ ppm: 24.48 (CH_3_), 112.46, 116.37 (d, ^2^J_CF_ = 22.6 Hz), 119.07, 120.86, 127.12, 128.03, 128.16, 129.98, 130.03, 130.16, 133.08, 134.84, 136.62, 141.81, 142.21, 143.25, 155.61 (Hydrazide C=O), 161.43 (d, ^1^J_CF_ = 244.4 Hz), 162.88 (Isatin C=O), 169.46 (Acetamide C=O); anal. calcd. for C_25_H_17_ClFN_7_O_3_ (517.91 g/mol): C, 57.98; H, 3.31; N, 18.93; found C, 57.92; H, 3.30; N, 18.98.

##### *N*-[4-(3-{*N*′-[5-Chloro-2-oxo-2,3-dihydro-1*H*-indol-3-ylidene]hydrazinecarbonyl}-1-(4-chlorophenyl)-1*H*-1,2,4-triazol-5-yl)phenyl]acetamide (**11i**)

Yellow powder (yield 77.4%); melt. pt. > 300 °C; ^1^H NMR (500 MHz, DMSO-d_6_) δ ppm: 2.05 (s, 3H, Methyl), 6.96 (dd, 1H, Ar. Proton, J = 8.4, 2.8 Hz), 7.41–7.45 (m, 1H, Ar. Proton), 7.50 (d, 2H, Ar. Proton, J = 8.8 Hz), 7.53–7.61 (m, 2.4H, Ar. Proton), 7.62–7.68 (m, 4H, Ar. Proton), 7.98 (s, 0.6H, Ar. Proton), 10.21 (s, 1H, Acetamide NH), 11.02, 11.39 (2s, 1H, Indolin-2-one NH), 11.88, 14.23 (2s, 1H, Hydrazide NH); ^13^C NMR (101 MHz, DMSO-d_6_) δ ppm: 24.51 (Methyl), 113.03, 117.13, 119.03, 120.82, 126.23, 128.04, 128.14, 129.92, 130.01, 130.18, 130.23, 132.99, 134.92, 136.72, 141.88, 142.01, 143.30, 155.12 (Hydrazide Carbonyl), 162.77 (Indolin-2-one Carbonyl), 169.40 (Acetamide Carbonyl); anal. calcd. for C_25_H_17_Cl_2_N_7_O_3_ (534.36 g/mol): C, 56.19; H, 3.21; N, 18.35; practical C, 56.36; H, 3.20; N, 18.28.

##### *N*-[4-(3-{*N*′-[5-Bromo-2-oxo-2,3-dihydro-1*H*-indol-3-ylidene]hydrazinecarbonyl}-1-(4-chlorophenyl)-1*H*-1,2,4-triazol-5-yl)phenyl]acetamide (**11j**)

Yellow powder (yield 72.7%); melt. pt. > 300 °C; ^1^H NMR (400 MHz, DMSO-d_6_) δ ppm: 2.07 (s, 3H, Methyl), 6.93 (d, 1H, Ar. Proton, J = 8.4 Hz), 7.44 (d, 1H, Ar. Proton, J = 8.7 Hz), 7.52–7.71 (m, 8.6H, Ar. Proton), 8.13 (s, 0.4H, Ar. Proton), 10.20 (s, 1H, Acetamide NH), 11.02, 11.39 (2s, 1H, Indolin-2-one NH), 11.85, 14.22 (2s, 1H, Hydrazide NH); anal. calcd. for C_25_H_17_BrClN_7_O_3_ (578.81 g/mol): C, 51.88; H, 2.96; N, 16.94; practical C, 51.92; H, 2.97; N, 16.98.

##### *N*-{4-[1-(4-Chlorophenyl)-3-{*N*′-[5-methoxy-2-oxo-2,3-dihydro-1*H*-indol-3-ylidene]hydrazinecarbonyl}-1*H*-1,2,4-triazol-5-yl]phenyl}acetamide (**11k**)

Orange powder (yield 70%); melt. pt. > 300 °C; ^1^H NMR (400 MHz, DMSO-d_6_) δ ppm: 2.07 (s, 3H, Methyl), 3.72, 3.79 (2s, 3H, Methoxy), 6.87 (d, 1H, Ar. Proton, J = 8.6 Hz), 6.98 (d, 0.6H, Ar. Proton, J = 8.6 Hz), 7.05 (d, 0.4H, Ar. Proton, J = 8.8 Hz), 7.16 (s, 1H, Ar. Proton), 7.43–7.48 (m, 2H, Ar. Proton), 7.55–7.59 (m, 2H, Ar. Proton), 7.64–7.66 (m, 4H, Ar. Proton), 10.20 (s, 1H, Acetamide NH), 10.69, 11.07 (2s, 1H, Indolin-2-one NH), 11.70, 14.30 (2s, 1H, Hydrazide NH); ^13^C NMR (101 MHz, DMSO-d_6_) δ ppm: 24.45 (Methyl), (55.88, 55.93 (Methoxy)), 106.21, 112.34, 112.49, 119.02, 119.56, 120.73, 120.93, 127.96, 128.06, 129.91, 130.11, 134.86, 136.64, 136.81, 138.22, 139.43, 141.78, 141.87, 142.15, 154.89, 155.33, 155.60, 155.75 (Hydrazide Carbonyl), 162.98, 164.90 (Indolin-2-one Carbonyl), 169.46 (Acetamide Carbonyl); anal. calcd. for C_26_H_20_ClN_7_O_4_ (529.94 g/mol): C, 58.93; H, 3.80; N, 18.50; practical C, 58.95; H, 3.78; N, 18.54.

##### *N*-{4-[1-(4-Chlorophenyl)-3-{*N*′-[5-nitro-2-oxo-2,3-dihydro-1*H*-indol-3-ylidene]hydrazinecarbonyl}-1*H*-1,2,4-triazol-5-yl]phenyl}acetamide (**11l**)

Yellow powder (yield 66.4%); melt. pt. > 300 °C; ^1^H NMR (400 MHz, DMSO-d_6_) δ ppm: 2.07 (s, 3H, Methyl), 7.12–7.18 (m, 1H, Ar. Proton), 7.44 (d, 1H, Ar. Proton, J = 8.9 Hz), 7.55–7.72 (m, 7H, Ar. Proton), 8.28–8.34 (m, 1H, Ar. Proton), 8.38 (dd, 0.5H, Ar. Proton, J = 8.7, 2.2 Hz), 8.85 (s, 0.5H, Ar. Proton), 10.20, 10.22 (2s, 1H, Acetamide NH), 11.59, 11.91 (2s, 1H, Indolin-2-one NH), 12.11, 14.11 (2s, 1H, Hydrazide NH); anal. calcd. for C_25_H_17_ClN_8_O_5_ (544.91 g/mol): C, 55.11; H, 3.14; N, 20.56; practical C, 55.13; H, 3.13; N, 20.50.

##### *N*-{4-[1-(4-Fluorophenyl)-3-{*N*′-[1-methyl-2-oxo-2,3-dihydro-1*H*-indol-3-ylidene]hydrazinecarbonyl}-1*H*-1,2,4-triazol-5-yl]phenyl}acetamide (**14a**)

Yellow powder (yield 39.2%); melt. pt. > 300 °C; ^1^H NMR (700 MHz, DMSO-d_6_) δ ppm: 2.08 (s, 3H, Acetamide Methyl), 3.24 (s, 3H, N-Methyl), 7.19 (s, 2H, Ar. Proton), 7.38–7.56 (m, 4.7H, Ar. Proton), 7.57–7.85 (m, 5H, Ar. Proton), 8.42 (s, 0.3H, Ar. Proton), 10.22, 10.25 (2s, 1H, Acetamide NH), 14.27 (s, 1H, Hydrazide NH); ^13^C NMR (176 MHz, DMSO-d_6_) δ ppm: 24.55 (Acetamide Methyl), 26.22 (N-Methyl), 110.48, 117.17 (d, ^2^J_CF_ = 23.3 Hz), 119.07, 119.53, 121.06, 121.31, 123.73, 127.36, 128.98 (d, ^3^J_CF_ = 9.5 Hz), 129.99, 130.31, 132.42, 134.34, 138.56, 141.86, 144.51, 154.83, 155.74 (Hydrazide Carbonyl), 161.33 (Indolin-2-one Carbonyl), 169.33 (Acetamide Carbonyl); anal. calcd. for C_26_H_20_FN_7_O_3_ (497.49 g/mol): C, 62.77; H, 4.05; N, 19.71; practical C, 62.62; H, 4.07; N, 19.79; HRMS (ESI) for C_26_H_21_FN_7_O_3_, calcd 498.1684, found 498.1691 [M+H]^+^, and for C_26_H_20_FN_7_NaO_3_, calcd 520.1504, found 520.1511 [M+Na]^+^.

##### *N*-[4-(3-{*N*′-[1-Benzyl-5-bromo-2-oxo-2,3-dihydro-1*H*-indol-3-ylidene]hydrazinecarbonyl}-1-(4-fluorophenyl)-1*H*-1,2,4-triazol-5-yl)phenyl]acetamide (**14b**)

Yellow powder (yield 71 melt. pt. > 300 °C; ^1^H NMR (700 MHz, DMSO-d_6_) δ ppm: 2.06, 2.07 (2s, 3H, Methyl), 5.02 (s, 2H, N-Methylene), 7.01 (d, 1H, Ar. Proton, J = 8.5 Hz), 7.29 (t, 1H, Ar. Proton, J = 7.3 Hz), 7.35 (t, 2H, Ar. Proton, J = 7.6 Hz), 7.39 (d, 2H, Ar. Proton, J = 7.4 Hz), 7.40–7.49 (m, 4H, Ar. Proton), 7.59–7.69 (m, 5H, Ar. Proton), 7.80 (d, 1H, Ar. Proton, J = 1.9 Hz), 10.21, 10.24 (2s, 1H, Acetamide NH), 14.19, 14.21 (2s, 1H, Hydrazide NH); ^13^C NMR (176 MHz, DMSO-d_6_) δ ppm: 24.55 (Methyl), 43.11 (N-Methylene), 113.00, 115.72, 117.19 (d, ^2^J_CF_ = 23.1 Hz), 119.09, 121.01, 121.93, 123.81, 127.39, 127.77, 127.78, 127.83, 128.14, 128.98 (d, ^3^J_CF_ = 9.3 Hz), 129.21, 130.03, 130.33, 134.29 (d, ^4^J_CF_ = 3.0 Hz), 134.52, 135.73, 137.31, 141.89, 142.56, 154.72, 155.77 (Hydrazide Carbonyl), 161.07 (Indolin-2-one Carbonyl), 162.08, 169.33 (Acetamide Carbonyl); anal. calcd. for C_32_H_23_BrFN_7_O_3_ (652.48 g/mol): C, 58.91; H, 3.55; N, 15.03; practical C, 58.91; H, 3.54; N, 15.09; HRMS (ESI) for C_32_H_24_BrFN_7_O_3_, calcd 652.1103, found 652.1100 [M+H]^+^, and for C_32_H_23_BrFN_7_NaO_3_, calcd 674.0922, found 674.0921 [M+Na]^+^.

##### *N*-{4-[1-(4-Chlorophenyl)-3-{*N*′-[1-methyl-2-oxo-2,3-dihydro-1*H*-indol-3-ylidene]hydrazinecarbonyl}-1*H*-1,2,4-triazol-5-yl]phenyl}acetamide (**14c**)

Yellow powder (yield 49%); melt. pt. > 300 °C; ^1^H NMR (400 MHz, DMSO-d_6_) δ ppm: 2.07 (s, 3H, Acetamide Methyl), 3.21, 3.24 (s, 3H, N-Methyl), 7.14–7.23 (m, 2H, Ar. Proton), 7.43–7.67 (m, 10H, Ar. Proton), 10.20 (s, 1H, Acetamide NH), 14.26 (s, 1H, Hydrazide NH); ^13^C NMR (101MHz, DMSO-d_6_) δ ppm: 24.51 (Acetamide Methyl), 26.17 (N-Methyl), 110.44, 119.11, 119.46, 121.00, 121.32, 123.73, 128.20, 130.04, 130.20, 132.69, 134.91, 136.69, 138.56, 141.87, 144.47, 154.96, 155.59, 155.68 (Hydrazide Carbonyl), 161.28 (Indolin-2-one Carbonyl), 169.42 (Acetamide Carbonyl); anal. calcd. for C_26_H_20_ClN_7_O_3_ (513.94 g/mol): C, 60.76; H, 3.92; N, 19.08; practical C, 60.73; H, 3.91; N, 19.11.

##### *N*-[4-(3-{*N*′-[1-Benzyl-5-bromo-2-oxo-2,3-dihydro-1*H*-indol-3-ylidene]hydrazinecarbonyl}-1-(4-chlorophenyl)-1*H*-1,2,4-triazol-5-yl)phenyl]acetamide (**14d**)

Yellow powder (yield 58.1%); melt. pt. > 300 °C; ^1^H NMR (400 MHz, DMSO-d_6_) δ ppm: 2.07 (s, 3H, Methyl), 5.02 (s, 2H, N-Methylene), 7.01 (d, 1H, Ar. Proton, J = 8.5 Hz), 7.25–7.48 (m, 7H, Ar. Proton), 7.54–7.68 (m, 7H, Ar. Proton), 7.78 (s, 1H, Ar. Proton), 10.20 (s, 1H, Acetamide NH), 14.17 (s, 1H, Hydrazide NH); ^3^C NMR (101MHz, DMSO-d_6_) δ ppm: 24.56 (Methyl), 43.14 (N-Methylene), 112.96, 115.72, 119.09, 120.91, 121.87, 123.77, 126.74, 127.78, 128.17, 129.19, 130.04, 130.21, 134.47, 134.92, 135.70, 136.83, 137.19, 141.96, 142.49, 147.16, 154.84, 155.66 (Hydrazide Carbonyl), 161.00 (Indolin-2-one Carbonyl), 169.29 (Acetamide Carbonyl); anal. calcd. for C_32_H_23_BrClN_7_O_3_ (668.94 g/mol): C, 57.46; H, 3.47; N, 14.66; practical C, 57.44; H, 3.46; N, 14.62.

### 3.2. Biological Evaluation

Evaluating the anti-proliferative activities for the herein disclosed 1,2,4-triazole-tethered indolin-2-ones **11a**–**l** and **14a**–**d** toward the examined cell lines (HepG2 and PANC1) was performed by utilizing the protocol of the MTT cytotoxicity assay, as previously described [35], whereas the VEGFR-2 inhibitory effect was assessed using VEGFR-2 Kinase Assay Kits (Cat. No. 40325 BPS Bioscience, San Diego, CA, USA) in accordance with the manufacturer’s instructions [21]. The examined human cancer HepG2 and PANC1 cell lines were obtained from the American Type Culture Collection (ATCC). All the utilized experimental procedures are provided in the Supplementary Materials.

### 3.3. Molecular Docking

The complete docking analysis utilized Vina Autodock 1.1.2 software to predict binding affinities and protein–ligand interactions [36]. The *.pdb format of the 3D crystal structure of 4ASD complexed with Sorafenib was obtained from RCSB PDB [28]. The details of the docking protocol are provided in the Supplementary Materials.

### 3.4. Molecular Dynamics

Three 100 ns molecular dynamic simulations (MDS) were executed using GROMACS 2023.2 software. Input structures for MDS were derived from the docking results and crystal coordinates of the VEGFR-2 enzyme complexed with compound **11d** and Sorafenib, respectively, as well as the apo VEGFR-2 [37,38,39].

### 3.5. In Silico ADME Study

The ADME study was conducted for all the synthesized compounds using SwissADME by using the compounds’ SMILES. Different physicochemical and pharmacokinetic parameters were calculated, and the ADME behavior was predicted.

## 4. Conclusions

Novel 1,2,4-triazole-tethered indolin-2-one congeners were designed based on the key structural features of the anti-VEGFR-2 drug, Sunitinib. These molecules were synthesized, characterized and biologically appraised for their anti-neoplastic activities against PANC1 and HepG2 cell lines. The synthesized indolin-2-one derivatives displayed moderate to potent anti-tumor activity, with IC_50_ values ranging from 0.17 to 4.29 µM for PANC1 and 0.58 to 4.49 µM for HepG2. The conducted SAR analysis revealed enhanced anti-cancer activity of the *N*-substituted indolin-2-one derivatives over the *N*-unsubstituted ones (with exception of compound **14b**). The potent analogs **11e**, **11d**, **11g**, **11k** and **14c** showed excellent VEGFR-2 inhibition in vitro (IC_50_ ranged from 16.3 to 119.6 nM). Interestingly, Compound **11d** (IC_50_ = 16.3 nM) emerged as the most active analog with superior activity over Sorafenib (IC_50_ = 29.7 nM). The in silico ADME study revealed the underlying drug-likeness of the synthesized compounds. Finally, the molecular docking study conducted for compound **11d** illustrating the binding mode and interactions with the active site greatly supports the biological results. The preceding findings strongly promote the optimization of 1,2,4-triazole-indolin-2-one hybrids for further discharging of novel VEGFR-2 inhibitors with substantial targeted anti-cancer activity.

## Figures and Tables

**Table 1:** In vitro anti-proliferative activities (IC_50_) for the synthesized indolin-2-one derivatives **11a**–**l** and **14a**–**d** against PANC1 and HepG2 cell lines.

	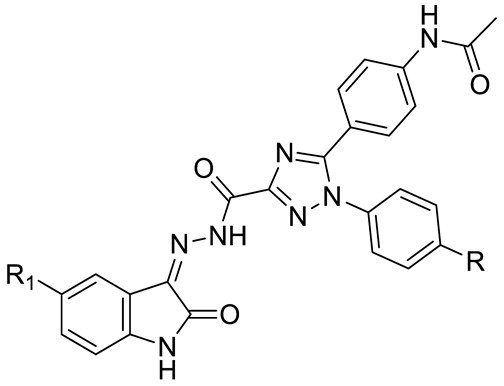 11a–l			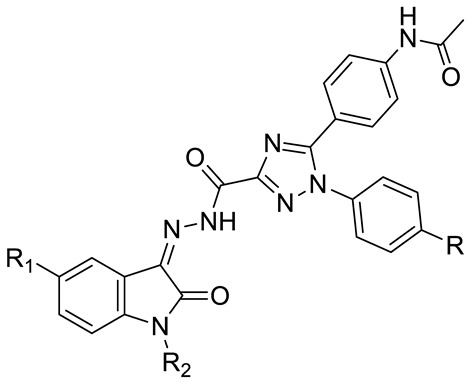 14a–d	
Comp.	R	R_1_	R_2_	IC_50_ (µM) ^a^	
				PANC1	HepG2
**11a**	F	H	---	4.29 ± 0.1	2.06 ± 0.03
**11b**	F	F	---	1.74 ± 0.02	1.17 ± 0.03
**11c**	F	Cl	---	0.98 ± 0.06	2.21 ± 0.03
**11d**	F	Br	---	1.16 ± 0.02	0.73 ± 0.02
**11e**	F	OCH_3_	---	2.22 ± 0.07	0.76 ± 0.02
**11f**	F	NO_2_	---	0.23 ± 0.01	2.41 ± 0.04
**11g**	Cl	H	---	0.77 ± 0.02	0.71 ± 0.01
**11h**	Cl	F	---	0.22 ± 0.01	1.27 ± 0.04
**11i**	Cl	Cl	---	3.75 ± 0.09	4.49 ± 0.15
**11j**	Cl	Br	---	1.68 ± 0.06	1.01 ± 0.02
**11k**	Cl	OCH_3_	---	1.78 ± 0.04	0.73 ± 0.01
**11l**	Cl	NO_2_	---	0.17 ± 0.01	1.73 ± 0.06
**14a**	F	H	CH_3_	4.05 ± 0.1	1.63 ± 0.02
**14b**	F	Br	C_6_H_5_	0.76 ± 0.04	0.98 ± 0
**14c**	Cl	H	CH_3_	0.2 ± 0	0.58 ± 0.01
**14d**	Cl	Br	C_6_H_5_	0.42 ± 0.01	0.92 ± 0.02
**Dox.**				0.19 ± 0.01	0.43 ± 0.02

^a^ IC_50_ values are presented as means ± S.D. for 3 separate experiments.

**Table 2:** Cytotoxic activities against non-tumorigenic Vero cell line.

Comp.	IC_50_ (µM)
**11d**	8.35 ± 0.62
**11e**	11.74 ± 0.93
**11g**	10.65 ± 0.71
**11k**	13.22 ± 1.09
**14c**	7.92 ± 0.48

**Table 3:** VEGFR-2 inhibitory action (IC_50_) of Sorafenib and indolin-2-one derivatives **11d**, **11e**, **11g**, **11k** and **14c**.

Comp.	VEGFR-2 IC_50_ (nM) ^a^
**11d**	16.3 ± 0.42
**11e**	48.4 ± 0.39
**11g**	119.6 ± 1.8
**11k**	41.3 ± 0.31
**14c**	53.8 ± 0.61
**Sorafenib**	29.7 ± 0.39

**^a^** IC_50_ values are the mean ± SD of three separate experiments.

**Table 4:** In silico physicochemical properties and drug-likeness of the synthesized compounds.

Compound	GI Absorption	BBB Permeant	Pgp Substrate	CYP1A2 Inhibitor	CYP2C19 Inhibitor	CYP2C9 Inhibitor	CYP2D6 Inhibitor	CYP3A4 Inhibitor	Lipinski #Violations	Veber #Violations
**11a**	High	No	No	No	Yes	Yes	No	Yes	0	0
**11b**	High	No	No	No	Yes	Yes	No	Yes	1	0
**11c**	High	No	No	No	Yes	Yes	No	Yes	1	0
**11d**	High	No	No	No	Yes	Yes	No	Yes	1	0
**11e**	Low	No	Yes	No	Yes	Yes	No	Yes	2	0
**11f**	Low	No	No	No	No	Yes	No	Yes	2	1
**11g**	High	No	No	No	Yes	Yes	No	Yes	0	0
**11h**	High	No	No	No	Yes	Yes	No	Yes	1	0
**11i**	High	No	No	No	Yes	Yes	No	Yes	1	0
**11j**	Low	No	No	No	Yes	Yes	No	Yes	1	0
**11k**	Low	No	No	No	Yes	Yes	No	Yes	2	0
**11l**	Low	No	No	No	No	Yes	No	Yes	2	1
**14a**	High	No	No	No	Yes	Yes	No	Yes	0	0
**14b**	Low	No	No	No	Yes	Yes	No	No	1	0
**14c**	High	No	No	No	Yes	Yes	No	Yes	1	0
**14d**	Low	No	No	No	Yes	No	No	No	2	0

**Table 5:** Docking energy scores for Sorafenib and target indolin-2-one-triazoles **11d**, **11e**, **11g**, **11k** and **14c**.

Compound	Docking Score (kcal/mol)
**11d**	−9.6
**11e**	−8.3
**11g**	−7.1
**11k**	−8.5
**14c**	−8.0
**Sorafenib**	−9.5

## Data Availability

Data are contained within the article and Supplementary Materials.

## Supplementary Materials

The following supporting information can be downloaded at: https://www.mdpi.com/article/10.3390/ph17010081/s1, S1: Cell viability assay; S2: VEGFR-2 kinase assay; S3: Molecular modeling; S4: HPLC Purity Analysis; S5: Physical and spectral data for target compounds.
