# Supplementary material for: 1,2,4-Triazole-Tethered Indolinones as New Cancer-Fighting Small Molecules Targeting VEGFR-2: Synthesis, Biological Evaluations and Molecular Docking
Source: Pharmaceuticals (Basel). 2024 Jan 8;17(1):81. doi: 10.3390/ph17010081 (PMC10820444; doi:10.3390/ph17010081)
Supplement: Supplementary file 1 [file pharmaceuticals-17-00081-s001.zip › pharmaceuticals-2754791-supplementary.pdf]

Supporting Materials

1,2,4-Triazole-tethered indolinones as new cancer-fighting small molecules targeting VEGFR-2: Synthesis, biological evaluations and molecular docking

Ahmed E. Elsawi[a], Mai I. Shahin[b], Hager A. Elbendary[c], Tarfah Al-Warhi[d], Fatma E. Hassan[e,f], Wagdy M. Eldehna[a,*]

[a] *Department of Pharmaceutical Chemistry, Faculty of Pharmacy, Kafrelsheikh University, Kafrelsheikh, P.O. Box 33516, Egypt*
[b] *Department of Pharmaceutical Chemistry, Faculty of Pharmacy, Ain Shams University, Abassia, Cairo 11566, Egypt*
[c] *Scientific Research and Innovation Support Unit, Faculty of Pharmacy, Kafrelsheikh University, Kafrelsheikh, Egypt*
[d] *Department of Chemistry, College of Science, Princess Nourah Bint Abdulrahman University, P.O. Box 84428, Riyadh 11671, Saudi Arabia*
[e] *Department of Physiology, General Medicine Practice Program, Batterjee Medical College, Jeddah 21442, Saudi Arabia*
[f] *Medical Physiology Department, Kasr Alainy, Faculty of Medicine, Cairo University, Giza 11562, Egypt*

*Corresponding author. E-mail addresses: wagdy2000@gmail.com (W.M. Eldehna).

1. Anti-proliferative activity against HepG2 and PANC1 cancer cell lines

The examined human cancer HepG2 and PANC1 cell lines have been obtained from American Type Culture Collection (ATCC). Cells lines were maintained as monolayers in Dulbecco's Modified Eagle's Medium (DMEM) supplemented with 10% FBS, 2 mM L-glutamine, 100 U/ml penicillin and 100µg/ml streptomycin sulfate. Cells were sub-cultured with trypsine /EDTA solution, counted with haemocytometer and plated onto 96-well plates (5000 cells/well) and left overnight to form a semi-confluent monolayer. Cell monolayers were treated in quadrates with vehicle (DMSO, 0.1% v/v), and test samples 1,2,4-triazole-tethered indolin-2-ones **11a-l** and **14a-d** for an exposure time of 48 h. At the end of exposure, MTT solution in PBS (5 mg/ml) was then added to all well including no cell blank and left to incubate for 90 min. The formation of formazan crystals were visually confirmed using phase contract microscopy. DMSO (100 µl/well) was added to dissolve the formazan crystals with shaking for 10 min after which the absorbance was read at 590 nm against no cell blanks on a FLuo Star Optima microplate reader (BMG technologies, Germany). Cell proliferation was calculated comparing the OD values of the DMSO control wells and those of the samples represented as % proliferation to the control. Dose-response experiment was performed on samples producing > or =50% loss of cell proliferation using five serial 2-fold dilutions (50, 25, 12.5, 6.25 and 3.125 µM) of the sample. IC_{50} values (concentration of sample causing 50% loss of cell proliferation of the vehicle control) were calculated using non-linear regression curve fitting of the dose response plots on GraphPad Prism V.6.0 software.

2. VEGFR-2 Kinase Assay

In vitro VEGFR-2 inhibitory activity of indolin-2-one derivatives **11d**, **11e**, **11g**, **11k** and **14c** was evaluated using serial dilutions (10, 5, 2.5, 1, 0.1, 0.01 μM) against VEGFR-2 Kinase Assay Kits (Cat. No.# 40325 BPS Bioscience) according to manufacturer's instructions. In brief, the master mixture was produced (25 μL per well) and poured into each well. Each well received 5 μL of inhibitor solution designated as "Test Inhibitor". The "Positive Control" and "Blank" groups received 5 μL of the same solution without the inhibitor (Inhibitor buffer). In order to prepare 3 mL of kinase buffer, 600 μL of kinase buffer were combined with 2400 μL of water. The blank wells received 20 μL of kinase buffer. The amount of VEGFR-2 required for the test was measured and the enzyme was diluted to 1 ng/ μL with kinase buffer. 20 μL of diluted VEGFR-2 enzymes was added to the wells designated as "Test Inhibitor Control" and "Positive Control" to start the reaction and the mixtures were incubated at 30 °C for 45 minutes. After the 45 minutes, each well received 50 μL of KinaseGlo Max reagent and the plate was incubated at room temperature for 15 minutes. The luminescence was measured with a microplate reader.

The autophosphorylation percentage inhibition by compounds was calculated using the following equation:

$$100\% - [(negative\ control)/(positive\ control) - (negative\ control)]$$

Using the curves of percentage inhibition of five concentrations of each compound, IC_{50} was calculated.

3. Molecular Docking

The complete docking analysis utilized Vina Autodock software to predict binding affinities and protein-ligand interactions. The *.pdb format of the 3D crystal structure of 4ASD complexed with Sorafenib was obtained in from RCSB PDB. The protein structural file was subsequently dehydrated and separated from its cocrystallized ligand to save them individually in *.pdb format. MarvinSketch was used to draw the proposed compound **11d**. Since Vina Autodock necessitates the target protein and ligands to be in pdbqt format, MGL tools were created to generate the required *.pdbqt files. The exhaustiveness parameter was set to 32, and the grid box size was 20Åx20Åx20Å, with the center based on the co-crystallized ligand's position in the VEGFR-2 active site. Protocol validation was conducted by the re-docking of the co-crystallized ligand, and the RMSD values were calculated using AutoDock Tools. The previous steps resulted in an RMSD value of 1.15 Å, highlighting the docking validity. Finally, compound **11d** was docked into the VEGFR-2 pre-established active site. The screened compound results were analyzed and visualized using Discovery Studio Visualizer software which generated 2D and 3D interactions for the docked poses.

3. Molecular Dynamics

Three 100 ns molecular dynamic simulations (MDS) were executed using GROMACS 2023.2 software. Input structures for MDS were derived from the docking results and crystal coordinates of the VEGFR-2 enzyme complexed with compound **11d** and Sorafenib, respectively, as well as the apo VEGFR-2. PDB2gmx within GROMACS and Acpype Server, respectively, generated receptor and ligand topologies using the AMBER force field. The ligands and receptor topologies were rejoined to create three distinct systems. The standard GROMACS molecular dynamics protocol was then applied to all systems, encompassing solvation, neutralization, energy minimization under the AMBER force field, and two equilibration stages (NVT and NPT). Subsequently, a 100 ns unrestricted production stage was initiated for the two systems, utilizing the Particle Mesh Ewald (PME) method to compute long-range electrostatic values with a 12 Å cut-off and 12 Å Fourier spacing. The stability of the complexes was assessed through RMSD, RMSF, and hydrogen bond stability calculations derived from MDS trajectories during the production phase.

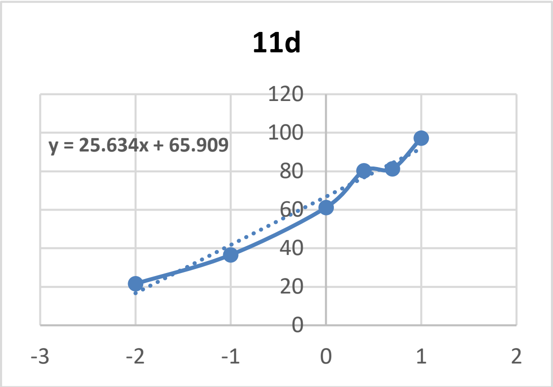
11d
y = 25.634x + 65.909
120
100
80
60
40
20
0
-3
-2
-1
0
1
2

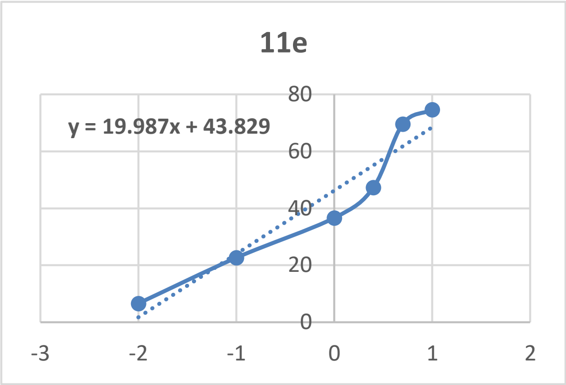
11e
y = 19.987x + 43.829
80
60
40
20
0
-3
-2
-1
0
1
2

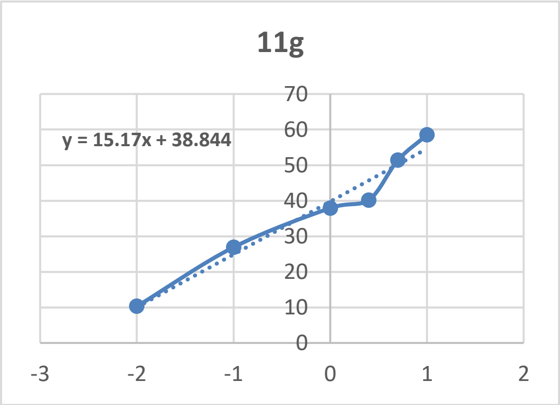
11g
y = 15.17x + 38.844
70
60
50
40
30
20
10
0
-3
-2
-1
0
1
2

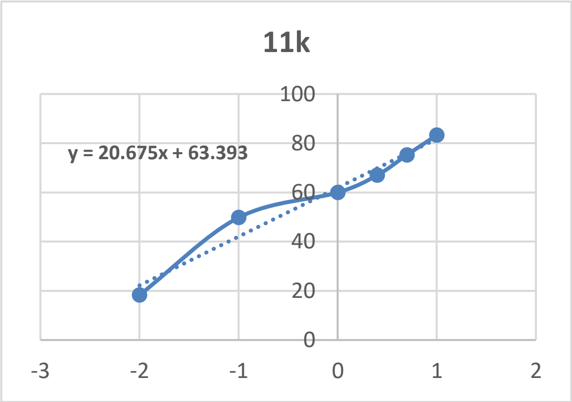
11k
y = 20.675x + 63.393
100
80
60
40
20
0
-3
-2
-1
0
1
2

HPLC Purity Analysis

Table S1. HPLC purity of the synthesized compounds **11e**, **11g**, **11k**, and **14c**.

Compound	Retention Time (minutes)	Purity (%)
**11e**	06:38	99.71
**11g**	06:52	97.41
**11k**	05:80	96.81
**14c**	07:50	99.70

Data File C:\CHEM32\1\DATA\11E-025.D
Sample Name: 11e

===

Acq. Operator : SYSTEM
Sample Operator : SYSTEM
Acq. Instrument : HPLC   Location : Vial 6
Injection Date : 12/16/2023 5:29:36 AM
Inj Volume : 5.000 µl
Acq. Method : C:\CHEM32\1\METHODS\DEF_LC.MCYANO COLUMN.M
Last changed : 12/16/2023 4:36:15 AM by SYSTEM
(modified after loading)
Analysis Method : C:\CHEM32\1\METHODS\DEF_LC.MCYANO COLUMN.M
Last changed : 12/16/2023 5:45:00 AM by SYSTEM
(modified after loading)
Sample Info : 40 ACN:60 phosphate buffer pH 3.7, Flow 1.50 mL/min, 254 nm, 5 ul injection

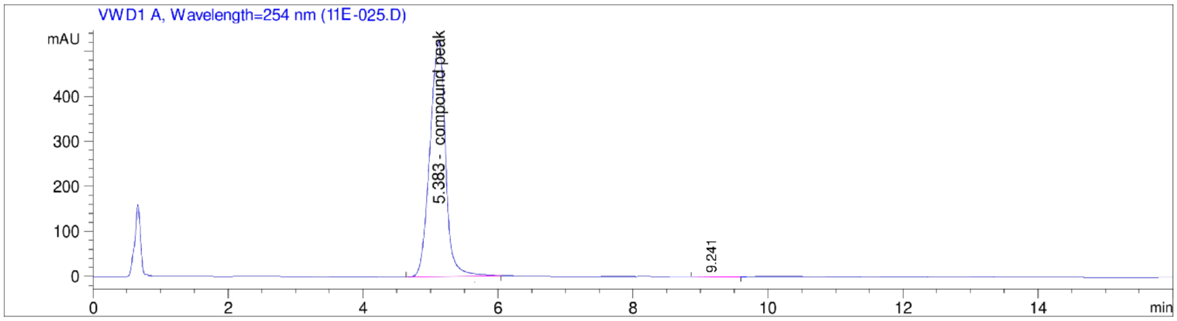

===

Area Percent Report

===

Sorted By : Signal
Calib. Data Modified : 12/16/2023 5:45:57 AM
Multiplier : 1.0000
Dilution : 1.0000
Sample Amount: : 0.500 [mg/ml] (not used in calc.)
Do not use Multiplier & Dilution Factor with ISTDs

Signal 1: VWD1 A, Wavelength=254 nm

Peak #	RetTime [min]	Type	Width [min]	Area [mAU*s]	Area %	Name
1	6.383	BBA	1.0098	1.94119e4	99.7154	Compound Peak

Totals : 1.94119e4 99.7154

*** End of Report ***

Data File C:\CHEM32\1\DATA\11G-027.D
Sample Name: 11g

===

Acq. Operator : SYSTEM
Sample Operator : SYSTEM
Acq. Instrument : HPLC Location : Vial 7
Injection Date : 12/16/2023 5:45:23 AM
Inj Volume : 5.000 µl
Acq. Method : C:\CHEM32\1\METHODS\DEF_LC.MCYANO COLUMN.M
Last changed : 12/16/2023 4:36:15 AM by SYSTEM
(modified after loading)
Analysis Method : C:\CHEM32\1\METHODS\DEF_LC.MCYANO COLUMN.M
Last changed : 12/16/2023 6:05:00 AM by SYSTEM
(modified after loading)
Sample Info : 40 ACN:60 phosphate buffer pH 3.7, Flow 1.50 mL/min, 254 nm, 5 ul injection

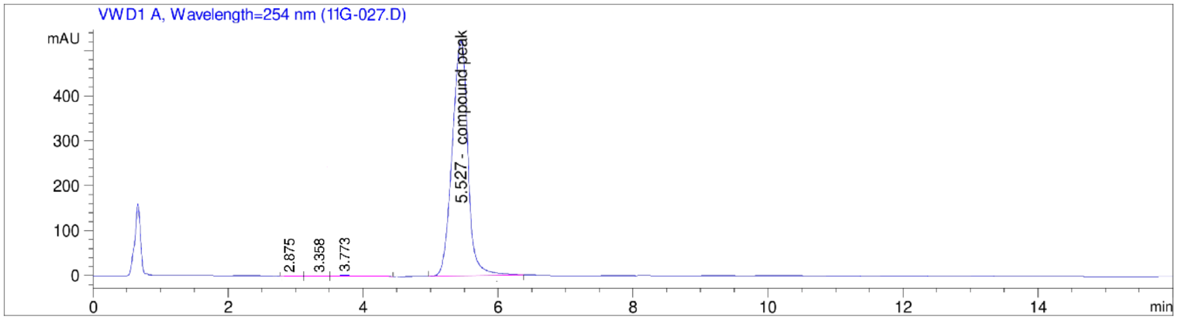

===

Area Percent Report

===

Sorted By : Signal
Calib. Data Modified : 12/16/2023 6:05:57 AM
Multiplier : 1.0000
Dilution : 1.0000
Sample Amount: : 0.500 [mg/ml] (not used in calc.)
Do not use Multiplier & Dilution Factor with ISTDs

Signal 1: VWD1 A, Wavelength=254 nm

Peak #	RetTime [min]	Type	Width [min]	Area [mAU*s]	Area %	Name
1	6.527	BBA	1.0108	1.99119e4	97.4151	Compound Peak

Totals : 1.99119e4 97.4151

*** End of Report ***

Data File C:\CHEM32\1\DATA\11K-031.D
Sample Name: 11K

===

Acq. Operator : SYSTEM
Sample Operator : SYSTEM
Acq. Instrument : HPLC Location : Vial 12
Injection Date : 12/16/2023 9:03:20 AM
Inj Volume : 5.000 µl
Acq. Method : C:\CHEM32\1\METHODS\DEF_LC.MCYANO COLUMN.M
Last changed : 12/16/2023 4:36:15 AM by SYSTEM
(modified after loading)
Analysis Method : C:\CHEM32\1\METHODS\DEF_LC.MCYANO COLUMN.M
Last changed : 12/16/2023 9:30:00 AM by SYSTEM
(modified after loading)
Sample Info : 40 ACN:60 phosphate buffer pH 3.7, Flow 1.50 mL/min, 254 nm, 5 ul injection

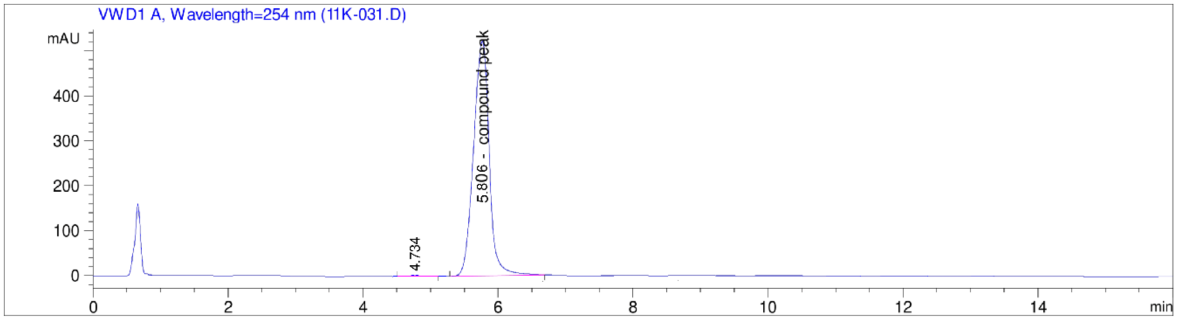

===

Area Percent Report

===

Sorted By : Signal
Calib. Data Modified : 12/16/2023 9:29:57 AM
Multiplier : 1.0000
Dilution : 1.0000
Sample Amount: : 0.500 [mg/ml] (not used in calc.)
Do not use Multiplier & Dilution Factor with ISTDs

Signal 1: VWD1 A, Wavelength=254 nm

Peak #	RetTime [min]	Type	Width [min]	Area [mAU*s]	Area %	Name
1	5.806	BBA	0.8918	1.19169e4	96.8145	Compound Peak
Totals :				1.19169e4	96.8145	

*** End of Report ***

Data File C:\CHEM32\1\DATA\14C-042.D
Sample Name: 14c

===

Acq. Operator : SYSTEM
Sample Operator : SYSTEM
Acq. Instrument : HPLC   Location : Vial 16
Injection Date : 12/16/2023 10:12:20 AM
Inj Volume : 5.000 µl
Acq. Method : C:\CHEM32\1\METHODS\DEF_LC.MCYANO COLUMN.M
Last changed : 12/16/2023 4:36:15 AM by SYSTEM
(modified after loading)
Analysis Method : C:\CHEM32\1\METHODS\DEF_LC.MCYANO COLUMN.M
Last changed : 12/16/2023 10:31:00 AM by SYSTEM
(modified after loading)
Sample Info : 40 ACN:60 phosphate buffer pH 3.7, Flow 1.50 mL/min, 254 nm, 5 ul injection

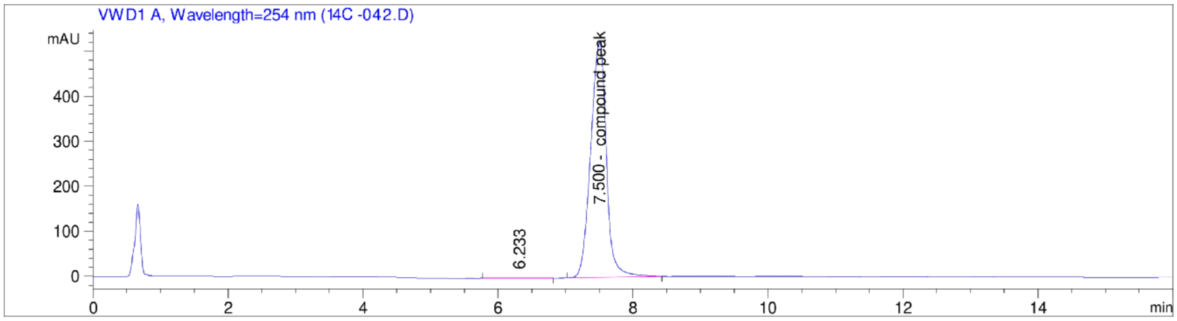

===
Area Percent Report
===

Sorted By : Signal
Calib. Data Modified : 12/16/2023 10:30:19 AM
Multiplier : 1.0000
Dilution : 1.0000
Sample Amount: : 0.500 [mg/ml] (not used in calc.)
Do not use Multiplier & Dilution Factor with ISTDs

Signal 1: VWD1 A, Wavelength=254 nm

Peak #	RetTime [min]	Type	Width [min]	Area [mAU*s]	Area %	Name
1	7.500	BBA	1.9181	2.09105e4	99.7750	Compound Peak

Totals : 2.09105e4 99.7750

*** End of Report ***

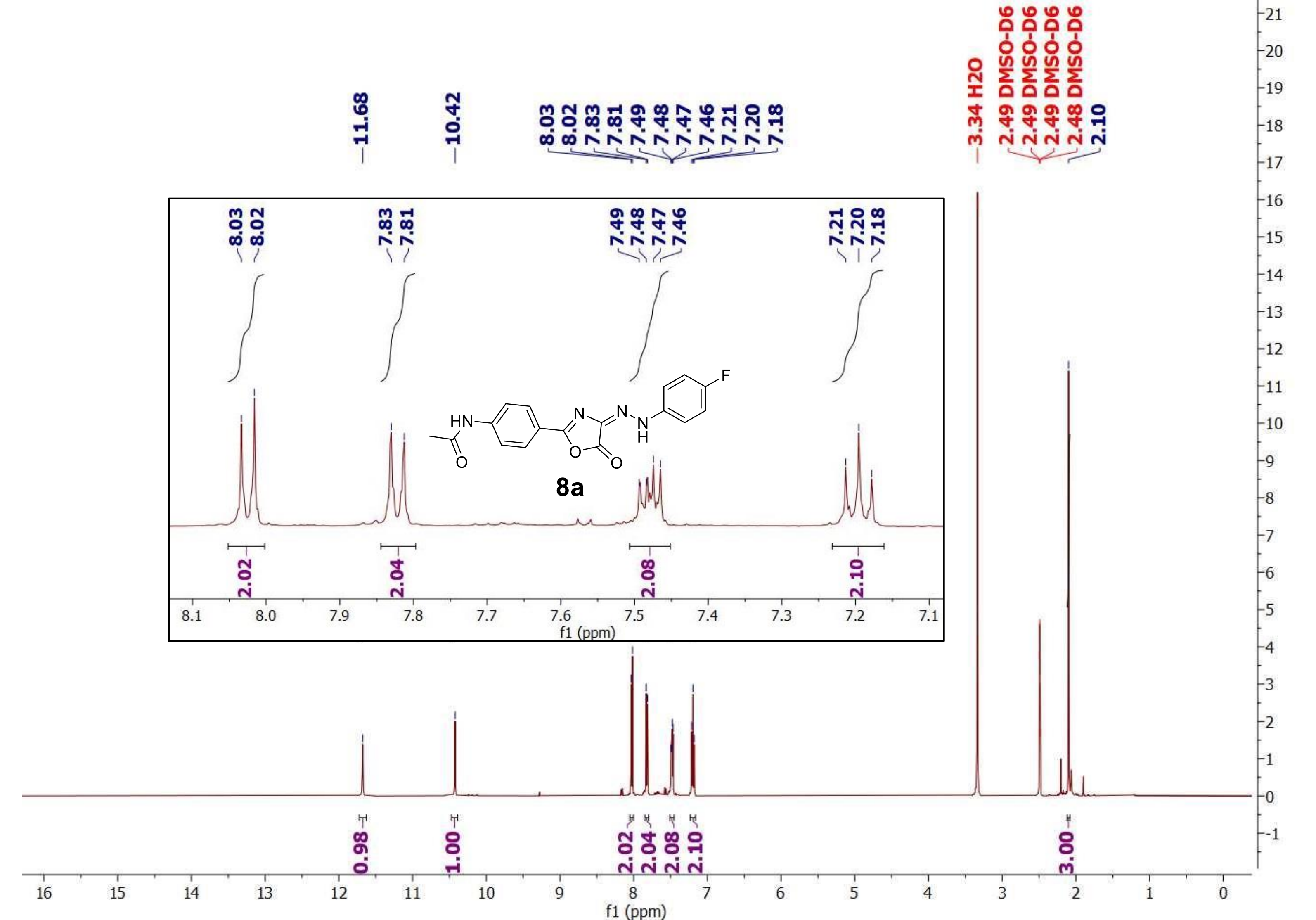

11.68
10.42
8.03 8.02 7.83 7.81 7.49 7.48 7.47 7.46 7.21 7.20 7.18
3.34 H2O
2.49 DMSO-D6
2.49 DMSO-D6
2.49 DMSO-D6
2.48 DMSO-D6
2.10
0.98
1.00
2.02
2.04
2.08
2.10
3.00
8a
f1 (ppm)

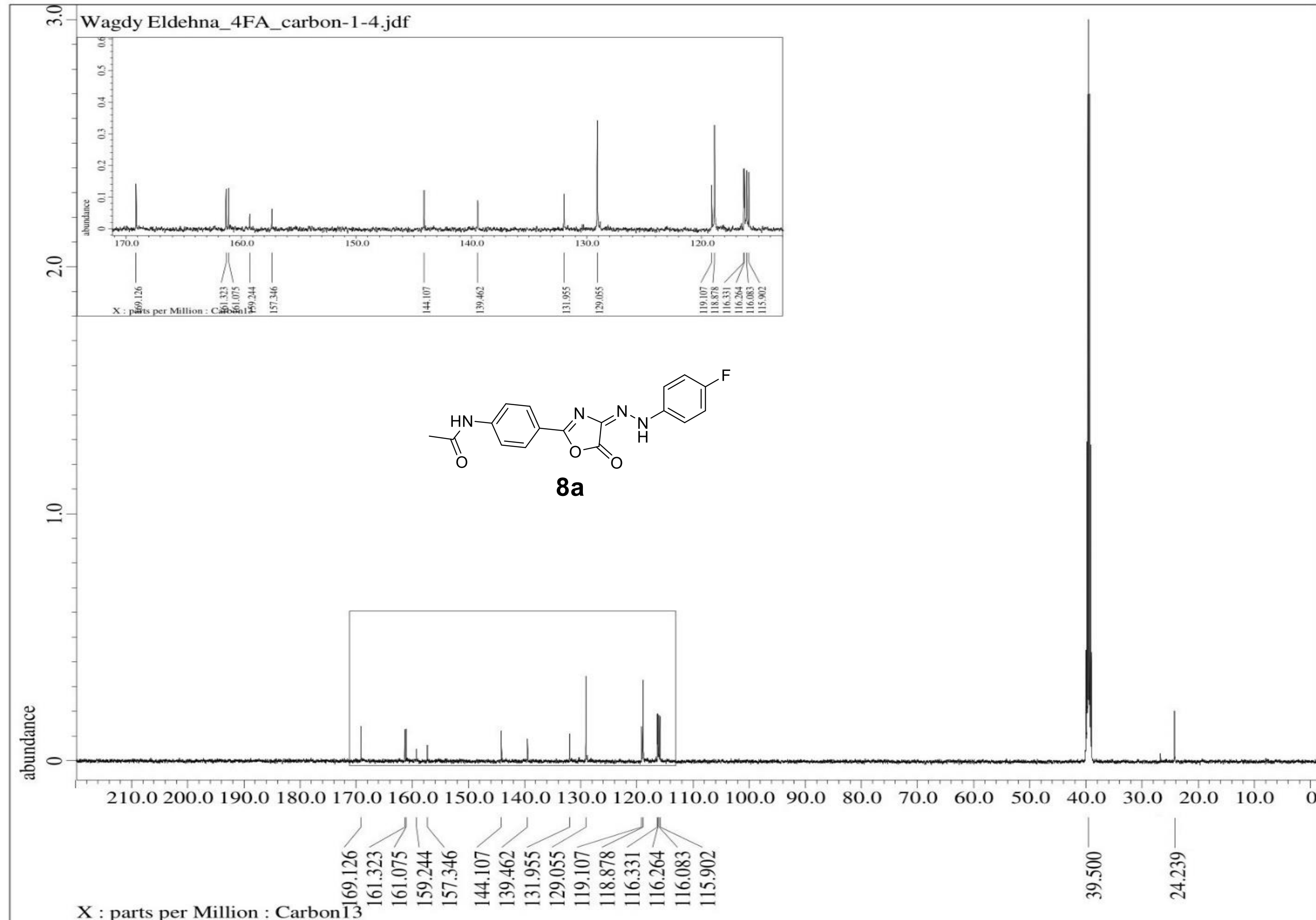
Wagdy Eldehna_4FA_carbon-1-4.jdf
abundance
X : parts per Million : Carbon13
169.126
161.323
161.075
159.244
157.346
144.107
139.462
131.955
129.055
119.107
118.878
116.331
116.264
116.083
115.902
39.500
24.239
HN
N
N
N
H
F
O
O
O
8a

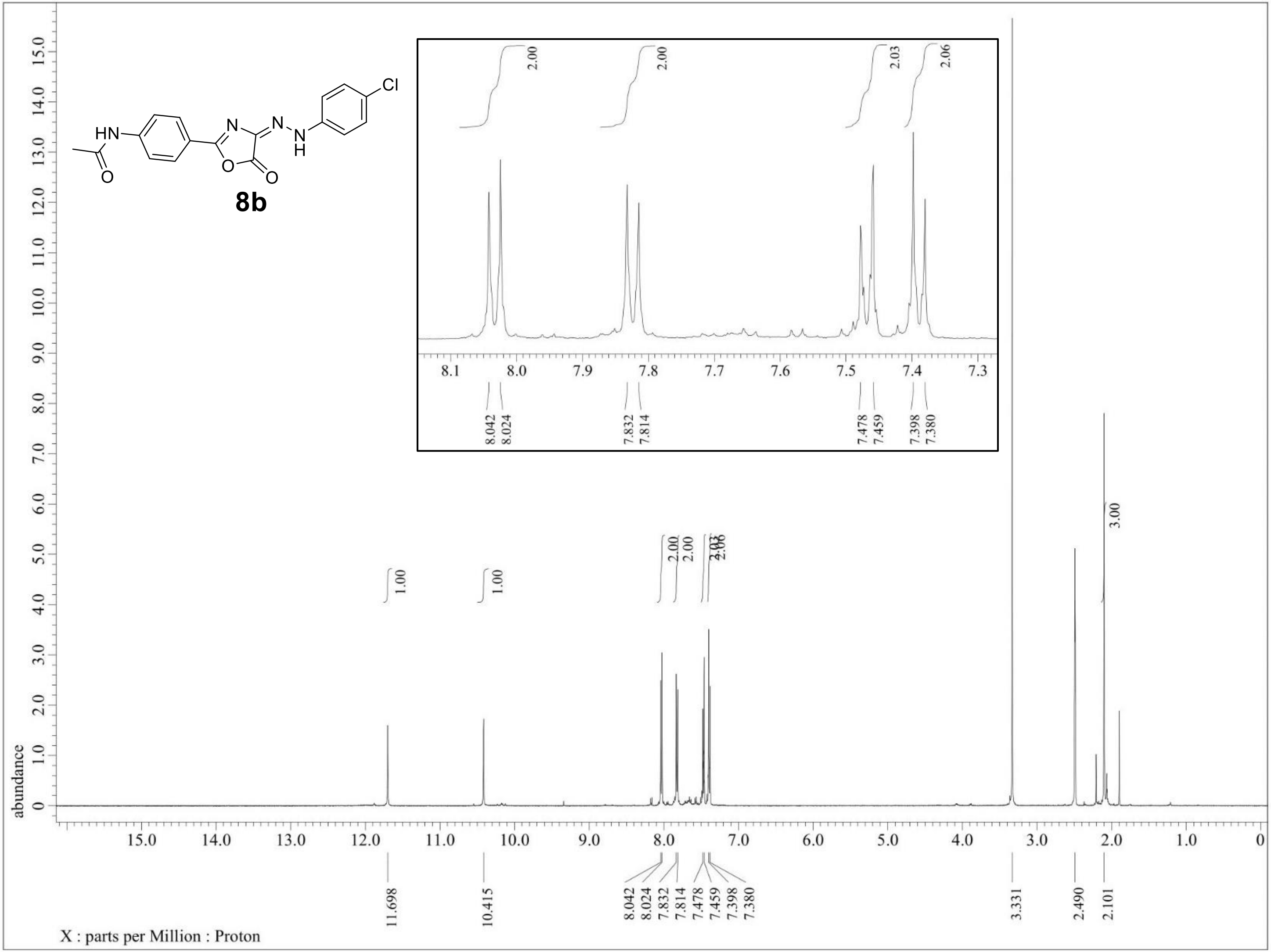
8b
HN
N
N
N
H
Cl
O
O
O
2.00
2.00
2.03
2.06
8.1
8.0
7.9
7.8
7.7
7.6
7.5
7.4
7.3
8.042
8.024
7.832
7.814
7.478
7.459
7.398
7.380
1.00
1.00
3.00
abundance
15.0
14.0
13.0
12.0
11.0
10.0
9.0
8.0
7.0
6.0
5.0
4.0
3.0
2.0
1.0
0
11.698
10.415
3.331
2.490
2.101
X : parts per Million : Proton

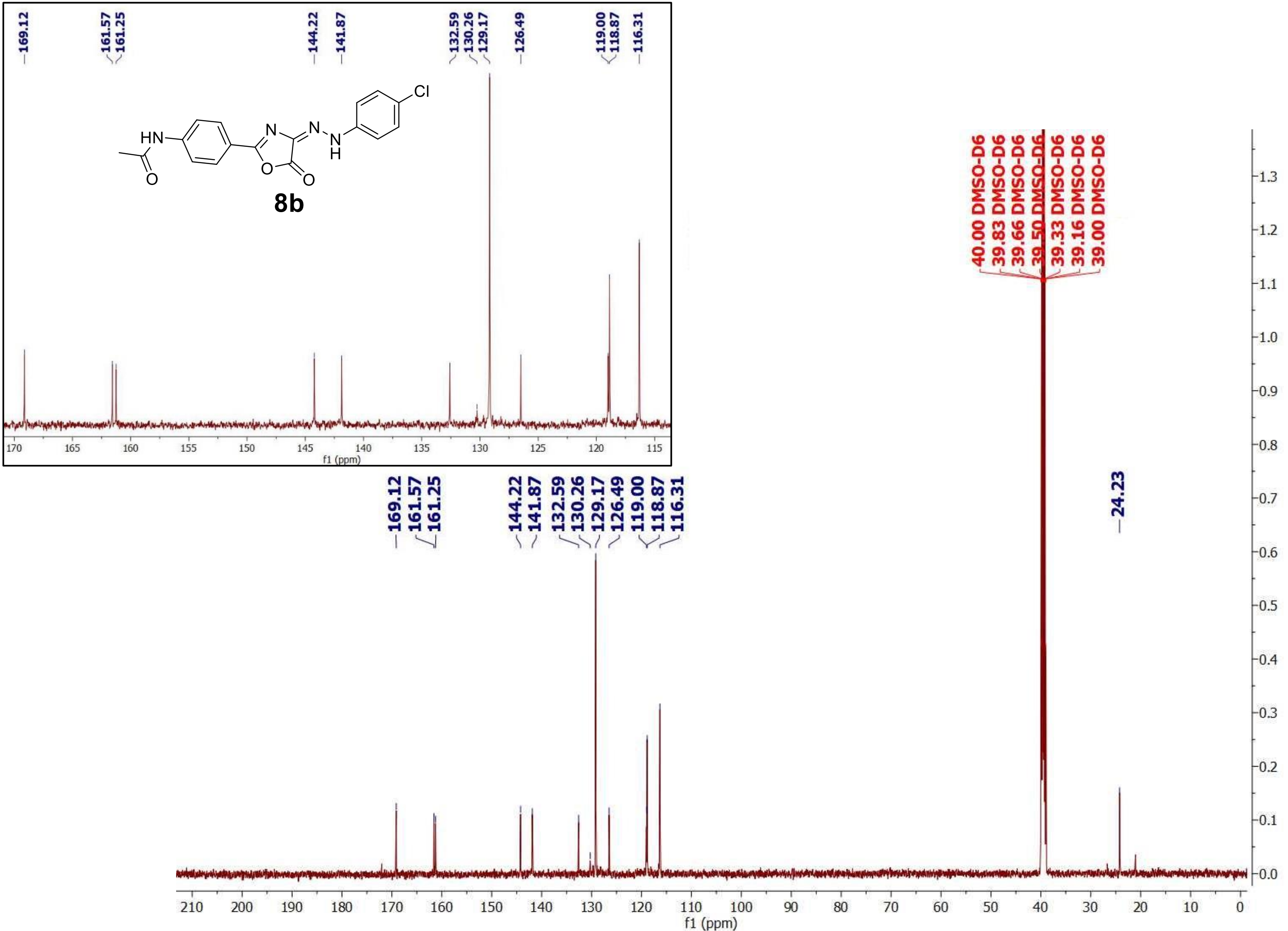
169.12
161.57
161.25
144.22
141.87
132.59
130.26
129.17
126.49
119.00
118.87
116.31
HN
N
N
N
H
O
O
O
Cl
8b
f1 (ppm)
40.00 DMSO-D6
39.83 DMSO-D6
39.66 DMSO-D6
39.50 DMSO-D6
39.33 DMSO-D6
39.16 DMSO-D6
39.00 DMSO-D6
24.23
f1 (ppm)

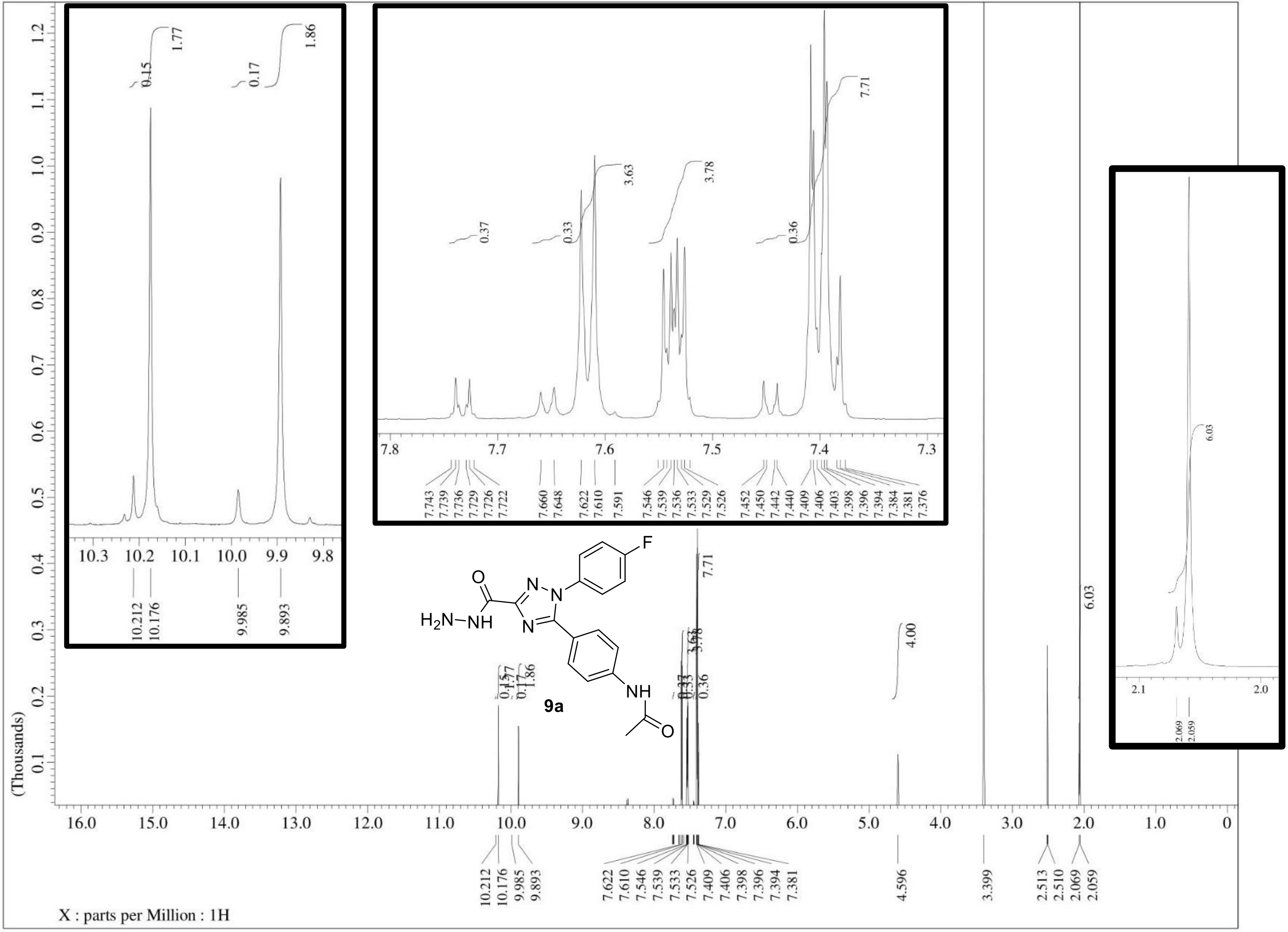

9a
F
O
N
N
H2N–NH
N
NH
O
10.212
10.176
9.985
9.893
10.3 10.2 10.1 10.0 9.9 9.8
0.15
1.77
0.17
1.86
7.8 7.7 7.6 7.5 7.4 7.3
7.743 7.739 7.736 7.729 7.726 7.722
7.660 7.648
7.622 7.610
7.591
7.546 7.539 7.536 7.533 7.529 7.526
7.452 7.450 7.442 7.440
7.409 7.406 7.403 7.398 7.396 7.394 7.384 7.381 7.376
0.37
0.33
3.63
3.78
0.36
7.71
2.1 2.0
2.069
2.059
6.03
4.00
(Thousands)
0.1 0.2 0.3 0.4 0.5 0.6 0.7 0.8 0.9 1.0 1.1 1.2
16.0 15.0 14.0 13.0 12.0 11.0 10.0 9.0 8.0 7.0 6.0 5.0 4.0 3.0 2.0 1.0 0
10.212 10.176 9.985 9.893
7.622 7.610 7.546 7.539 7.533 7.526 7.409 7.406 7.398 7.396 7.394 7.381
4.596
3.399
2.513 2.510
2.069 2.059
X : parts per Million : 1H

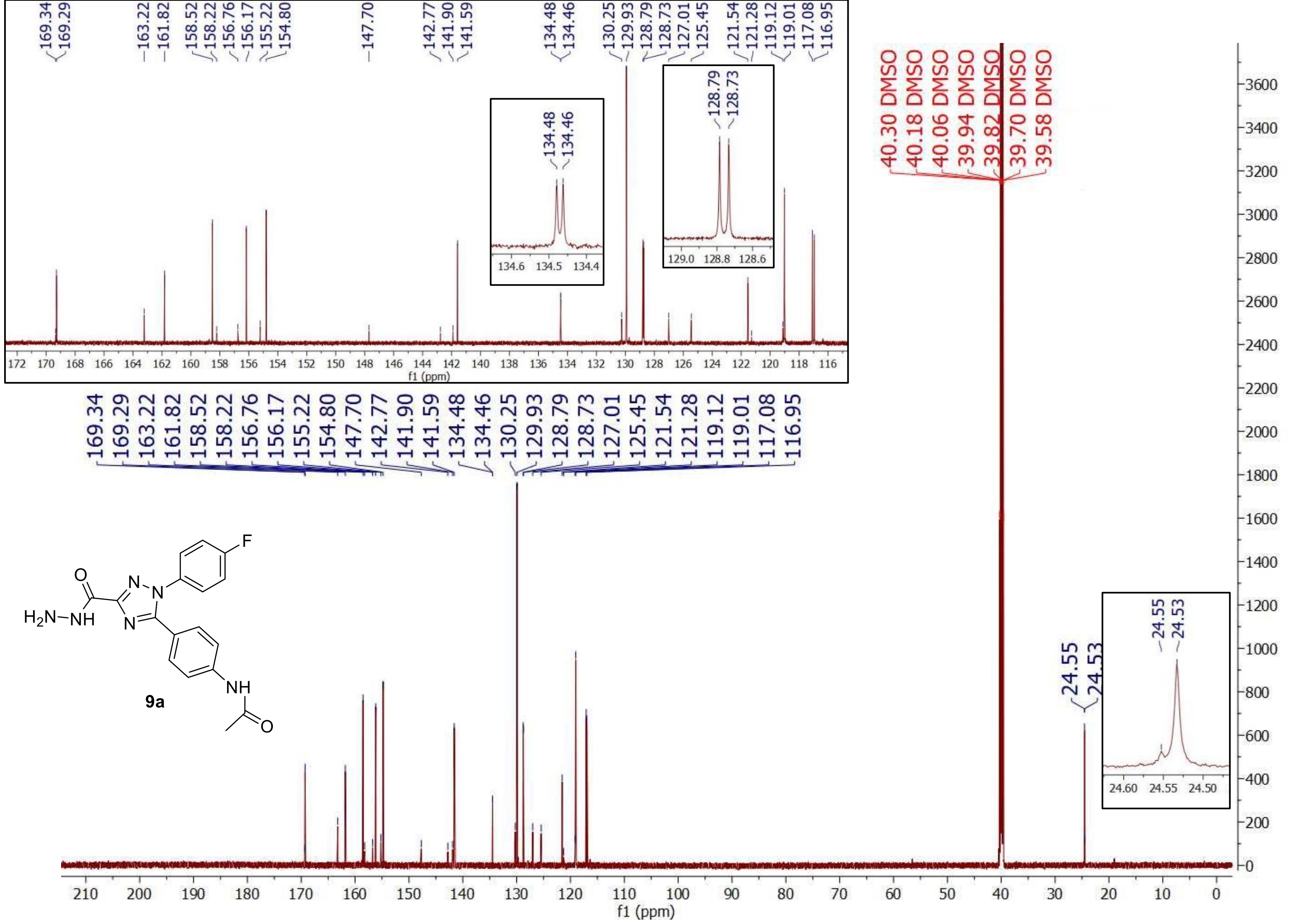
169.34
169.29
163.22
161.82
158.52
158.22
156.76
156.17
155.22
154.80
147.70
142.77
141.90
141.59
134.48
134.46
130.25
129.93
128.79
128.73
127.01
125.45
121.54
121.28
119.12
119.01
117.08
116.95
40.30 DMSO
40.18 DMSO
40.06 DMSO
39.94 DMSO
39.82 DMSO
39.70 DMSO
39.58 DMSO
24.55
24.53
f1 (ppm)
F
O
N
N
H_2N–NH
N
NH
O
9a

Item name: FAZ

Item name: FAZ, Sample position: 1:B,1, Replicate number: 1

.	Component name	Identification status	Formula	Neutral mass (Da)	Observed neutral mass (Da)	Observed m/z	Mass error (mDa)
1	FAZ	Identified	C17H15FO2N6	354.12405	354.1245	355.1318	0.5

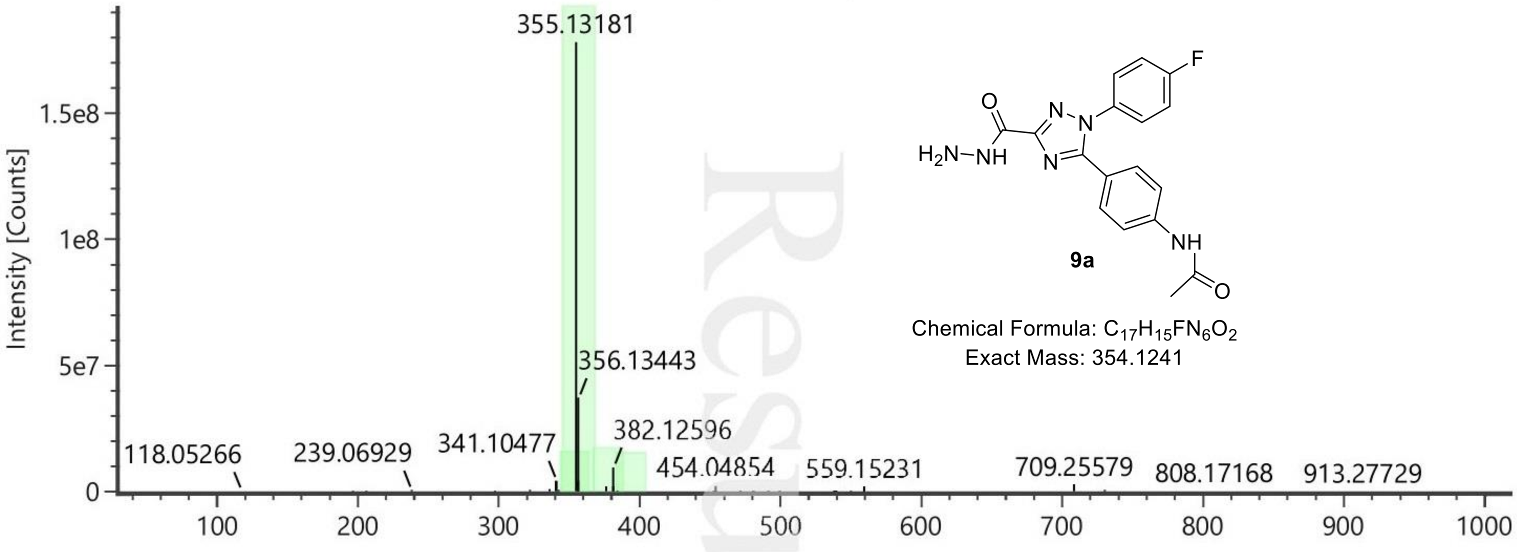

9a

Chemical Formula: $C_{17}H_{15}FN_6O_2$
Exact Mass: 354.1241

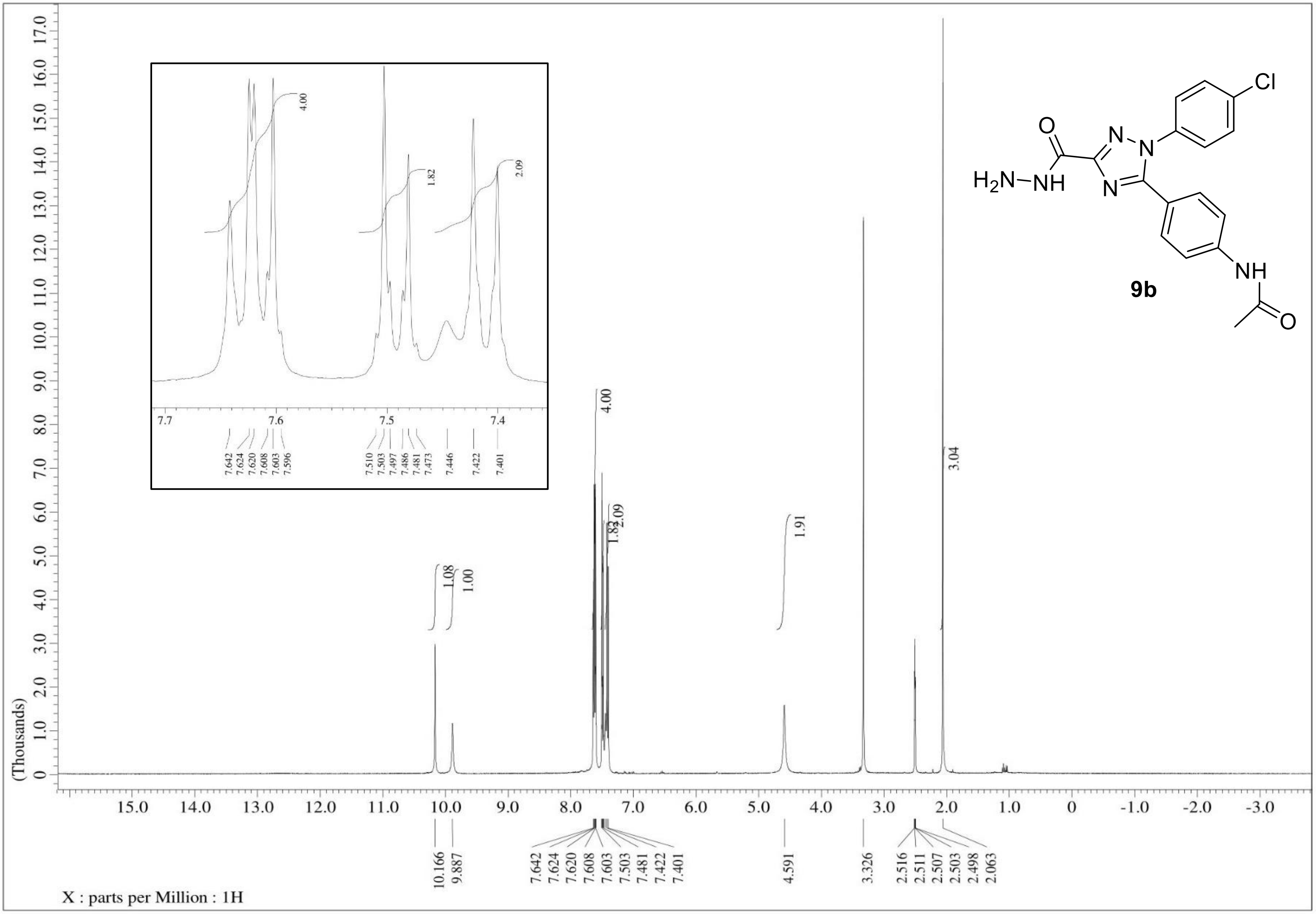

9b
X : parts per Million : 1H
(Thousands)
10.166
9.887
7.642
7.624
7.620
7.608
7.603
7.503
7.481
7.422
7.401
4.591
3.326
2.516
2.511
2.507
2.503
2.498
2.063

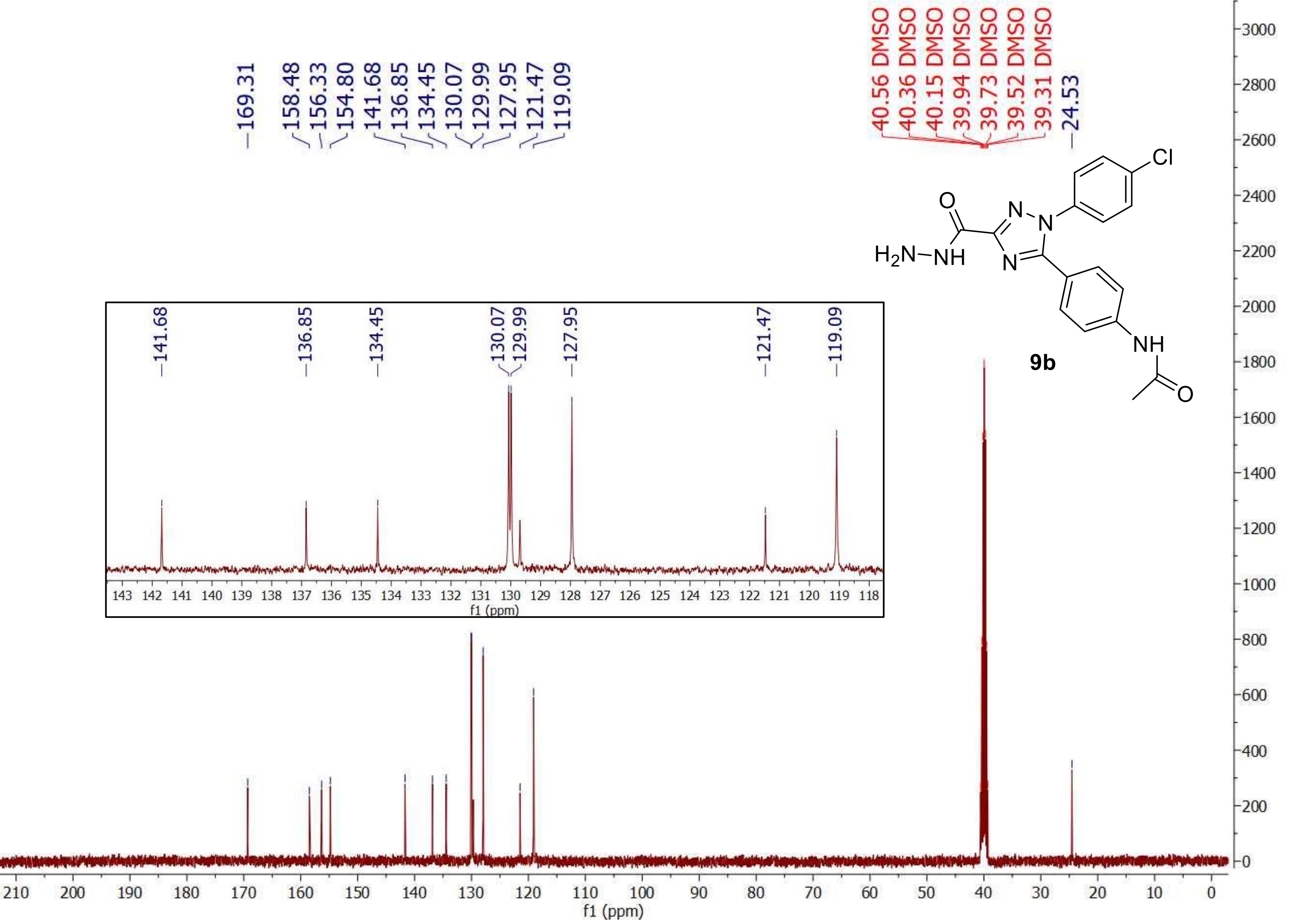
169.31
158.48
156.33
154.80
141.68
136.85
134.45
130.07
129.99
127.95
121.47
119.09
40.56 DMSO
40.36 DMSO
40.15 DMSO
39.94 DMSO
39.73 DMSO
39.52 DMSO
39.31 DMSO
24.53
O
N
N
N
Cl
H_2N–NH
NH
O
9b
f1 (ppm)
f1 (ppm)

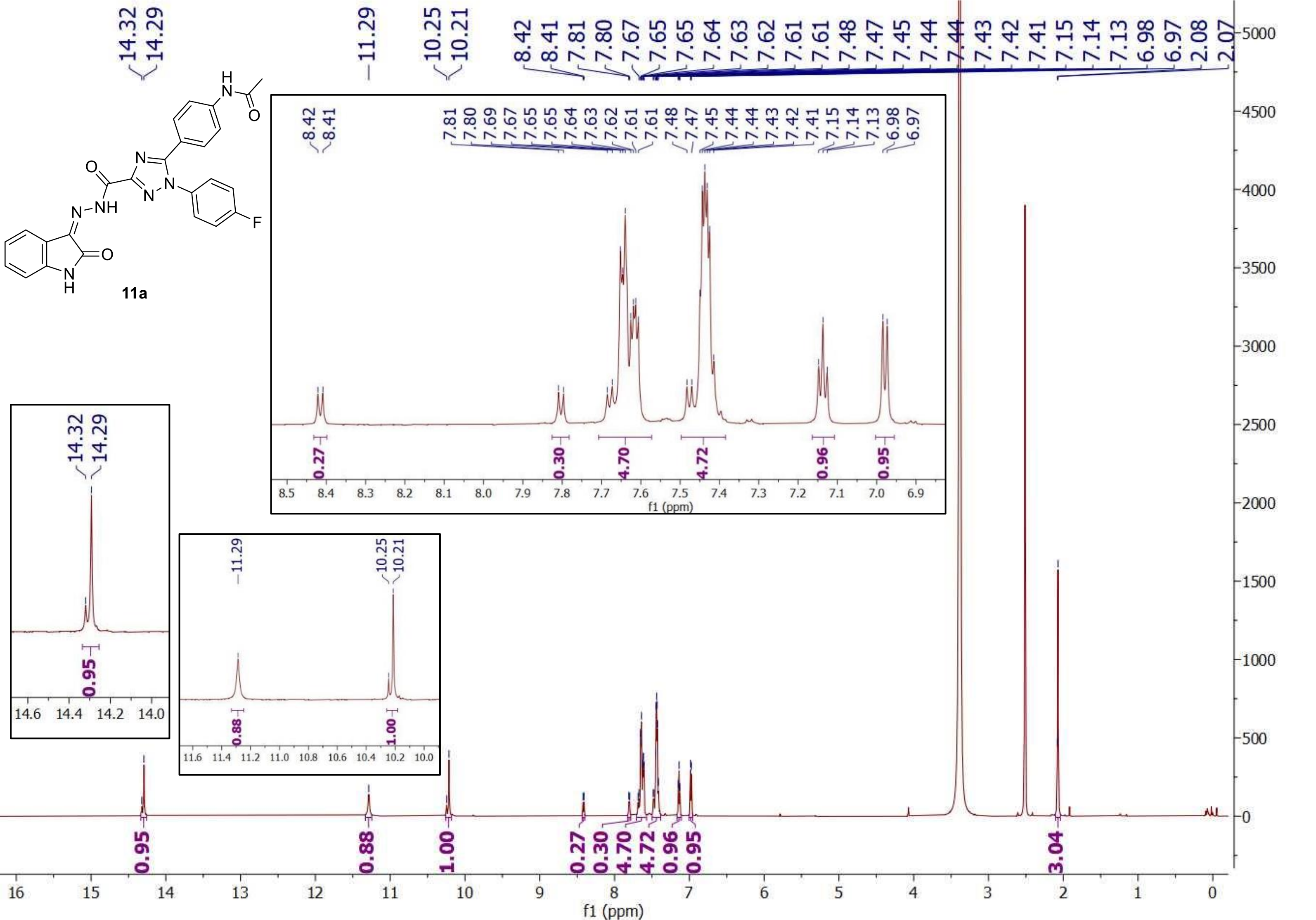

14.32
14.29
11.29
10.25
10.21
8.42 8.41 7.81 7.80 7.67 7.65 7.65 7.64 7.63 7.62 7.61 7.61 7.48 7.47 7.45 7.44 7.44 7.43 7.42 7.41 7.15 7.14 7.13 6.98 6.97 2.08 2.07
8.42 8.41 7.81 7.80 7.69 7.67 7.65 7.65 7.64 7.63 7.62 7.61 7.61 7.48 7.47 7.45 7.44 7.44 7.43 7.42 7.41 7.15 7.14 7.13 6.98 6.97
0.27 0.30 4.70 4.72 0.96 0.95
0.95
0.88 1.00
3.04
11a
f1 (ppm)

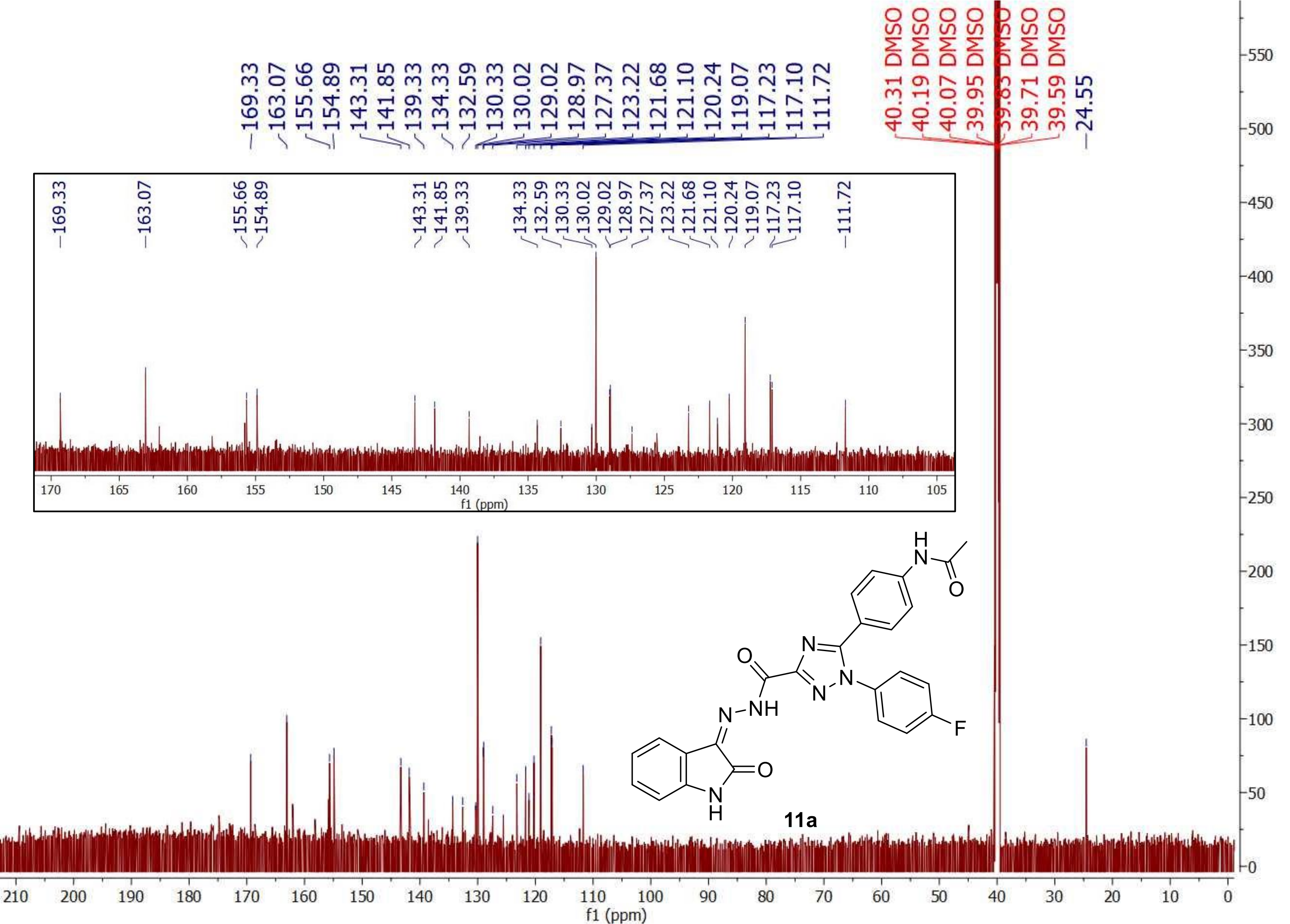

169.33
163.07
155.66
154.89
143.31
141.85
139.33
134.33
132.59
130.33
130.02
129.02
128.97
127.37
123.22
121.68
121.10
120.24
119.07
117.23
117.10
111.72
40.31 DMSO
40.19 DMSO
40.07 DMSO
39.95 DMSO
39.83 DMSO
39.71 DMSO
39.59 DMSO
24.55
f1 (ppm)
N
N
N
O
NH
N
H
N
O
F
H
N
O
H
11a

Compound Summary

Cpd	Name	Formula	RT	Mass	CAS	ID Source	Score	Score (Lib)	Score (DB)	Score (MFG)	Algorithm
1		C25 H18 F N7 O3	0.765	483.1459		FBF	99.54				FBF

Compound Details

Cpd. 1: C25 H18 F N7 O3

Name	Formula	RT	RI	Mass	Score	Algorithm	Lib/DB
	C25 H18 F N7 O3	0.765		483.1459	99.54	FBF	

Species	m/z	Score (Lib)	Num Spectra	Score (DB)	Score (MFG)	Score (RT)
(M+H)+ (M+Na)+	484.1532 506.1352					

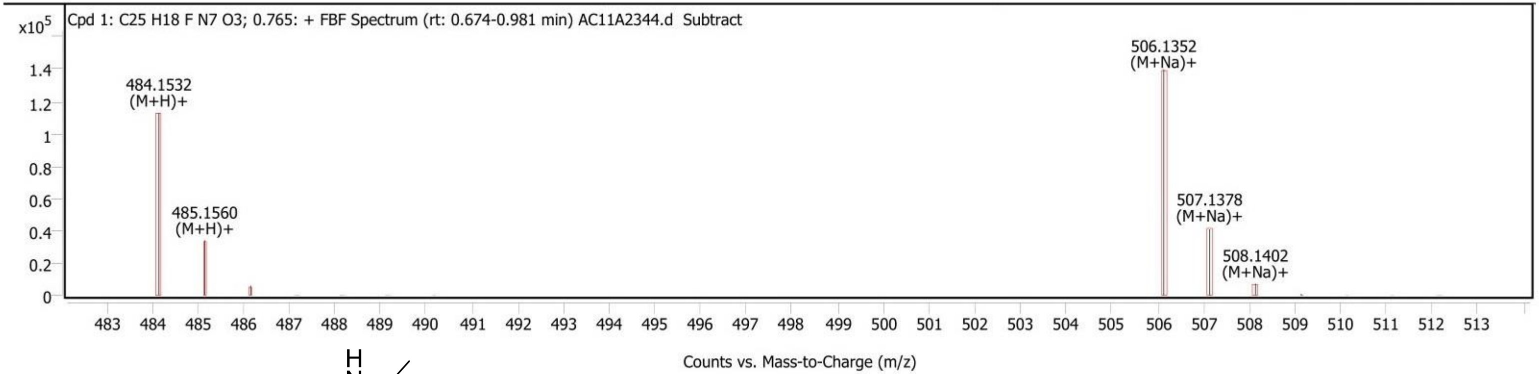

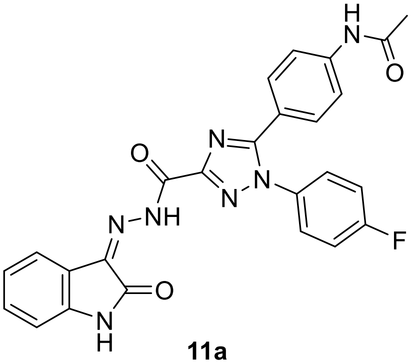

11a

Chemical Formula: $C_{25}H_{18}FN_7O_3$

Exact Mass: 483.1455

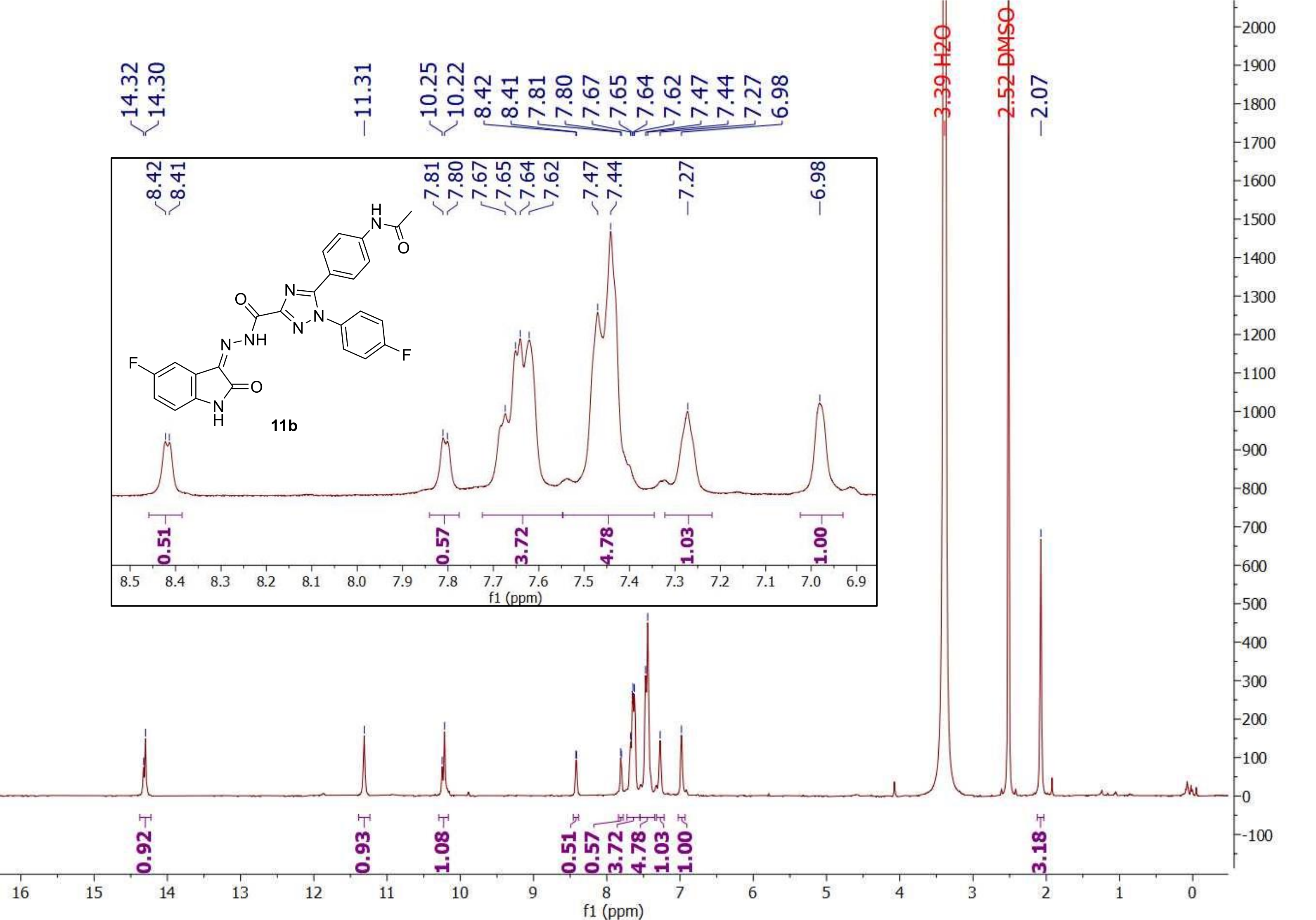

14.32
14.30
11.31
10.25
10.22
8.42
8.41
7.81
7.80
7.67
7.65
7.64
7.62
7.47
7.44
7.27
6.98
3.39 H2O
2.52 DMSO
2.07
11b
0.51
0.57
3.72
4.78
1.03
1.00
0.92
0.93
1.08
3.18
f1 (ppm)

Compound Summary

Cpd	Name	Formula	RT	Mass	CAS	ID Source	Score	Score (Lib)	Score (DB)	Score (MFG)	Algorithm
1		C25 H17 F2 N7 O3	0.762	501.1365		FBF	99.48				FBF

Compound Details

Cpd. 1: C25 H17 F2 N7 O3

Name	Formula	RT	RI	Mass	Score	Algorithm	Lib/DB
	C25 H17 F2 N7 O3	0.762		501.1365	99.48	FBF	

Species	m/z	Score (Lib)	Num Spectra	Score (DB)	Score (MFG)	Score (RT)
(M+H)+ (M+Na)+	502.1437 524.1256					

Compound Spectra

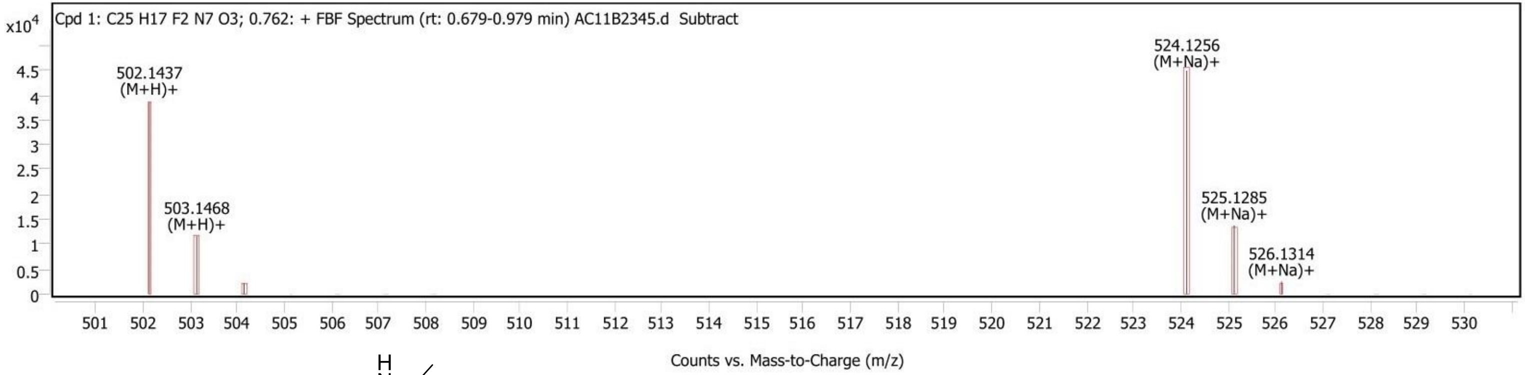

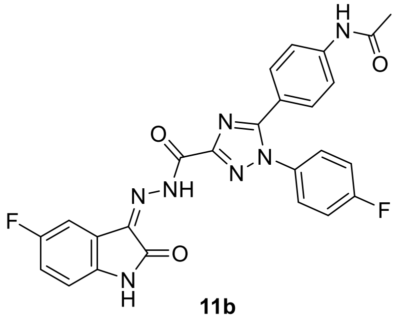

11b

Chemical Formula: $C_{25}H_{17}F_2N_7O_3$

Exact Mass: 501.1361

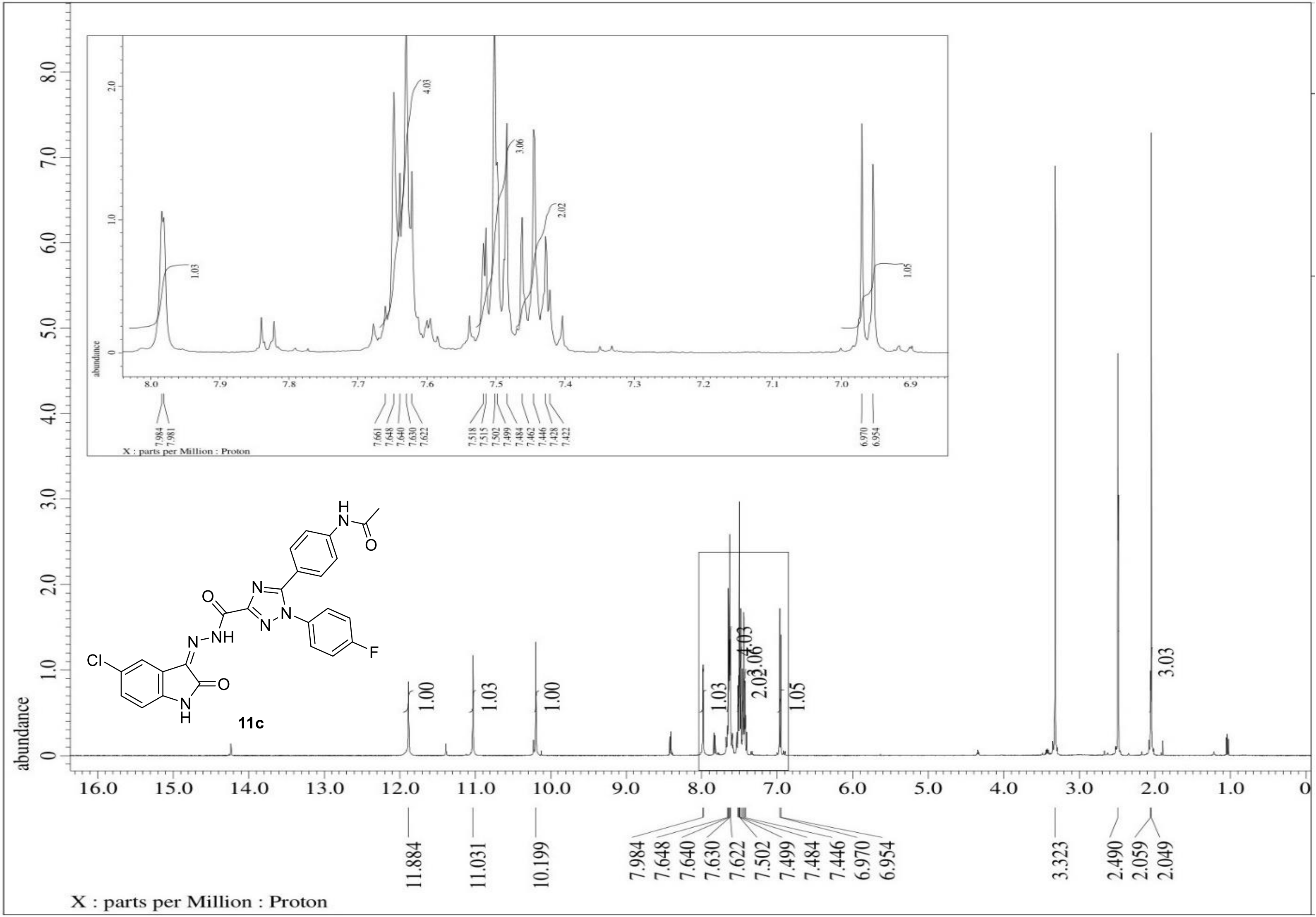
abundance
X : parts per Million : Proton
11c
Cl
N
NH
O
H
F
1.00
1.03
1.00
1.03
4.03
3.06
2.02
1.05
3.03
11.884
11.031
10.199
7.984
7.648
7.640
7.630
7.622
7.502
7.499
7.484
7.446
6.970
6.954
3.323
2.490
2.059
2.049
7.981
7.661
7.518
7.515
7.462
7.428
7.422

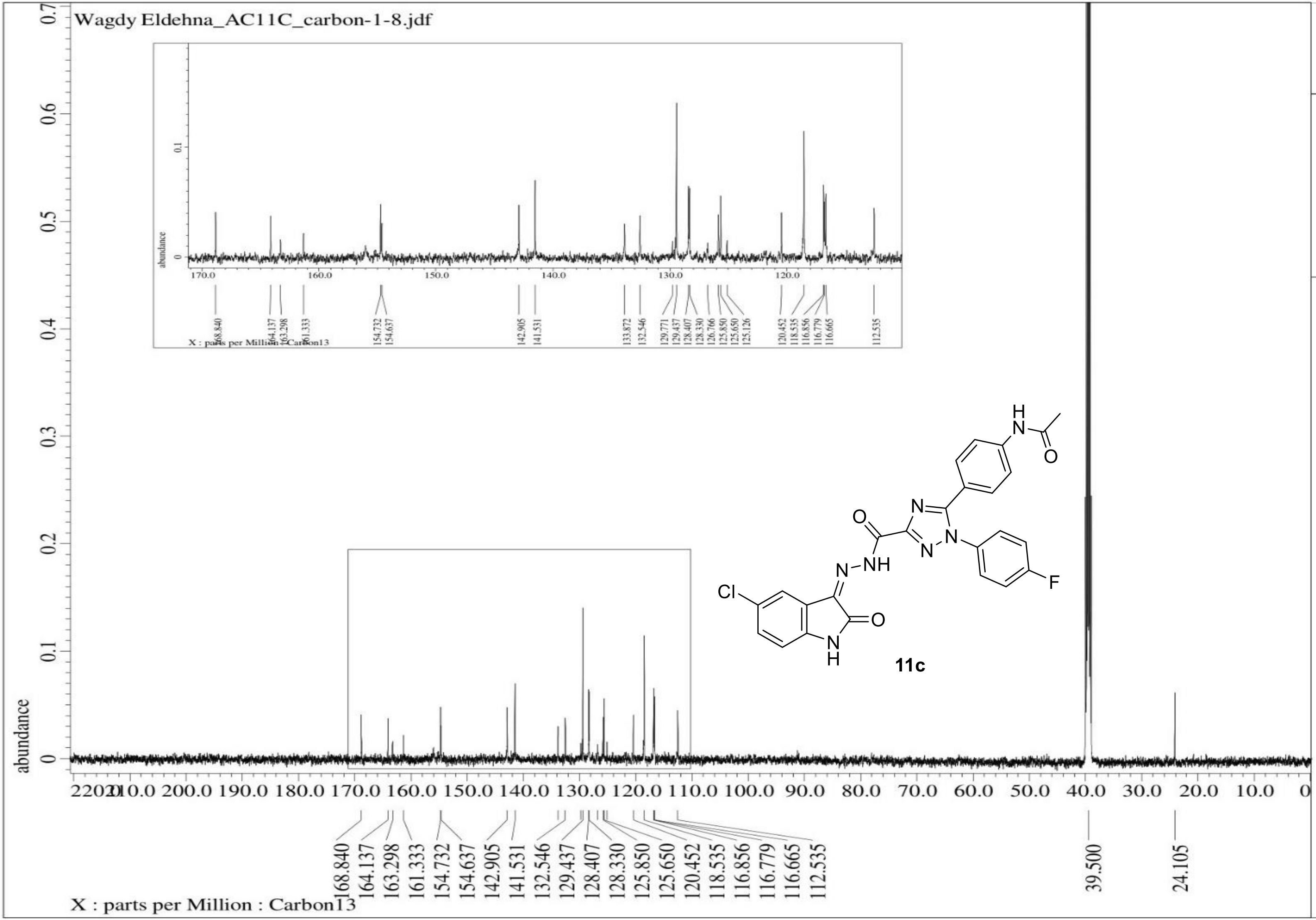

Wagdy Eldehna_AC11C_carbon-1-8.jdf
abundance
X : parts per Million : Carbon13
168.840
164.137
163.298
161.333
154.732
154.637
142.905
141.531
133.872
132.546
129.771
129.437
128.407
128.330
126.766
125.850
125.650
125.126
120.452
118.535
116.856
116.779
116.665
112.535
39.500
24.105
11c

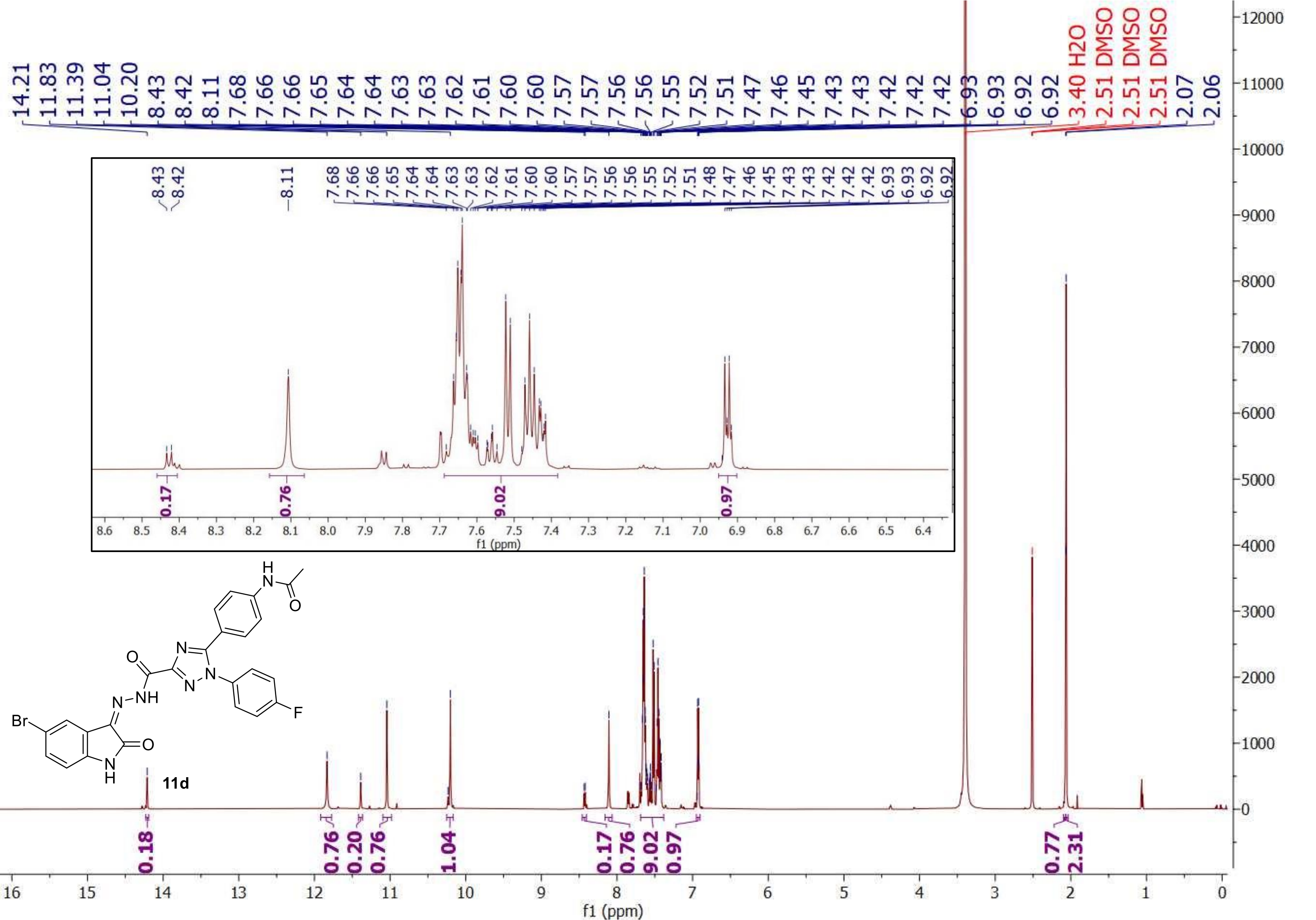

14.21
11.83
11.39
11.04
10.20
8.43
8.42
8.11
7.68
7.66
7.66
7.65
7.64
7.64
7.63
7.63
7.62
7.61
7.60
7.60
7.57
7.57
7.56
7.56
7.55
7.52
7.51
7.47
7.46
7.45
7.43
7.43
7.42
7.42
7.42
6.93
6.93
6.92
6.92
3.40 H2O
2.51 DMSO
2.51 DMSO
2.51 DMSO
2.07
2.06
0.17
0.76
9.02
0.97
f1 (ppm)
11d
0.18
0.76
0.20
0.76
1.04
0.17
0.76
9.02
0.97
0.77
2.31
f1 (ppm)

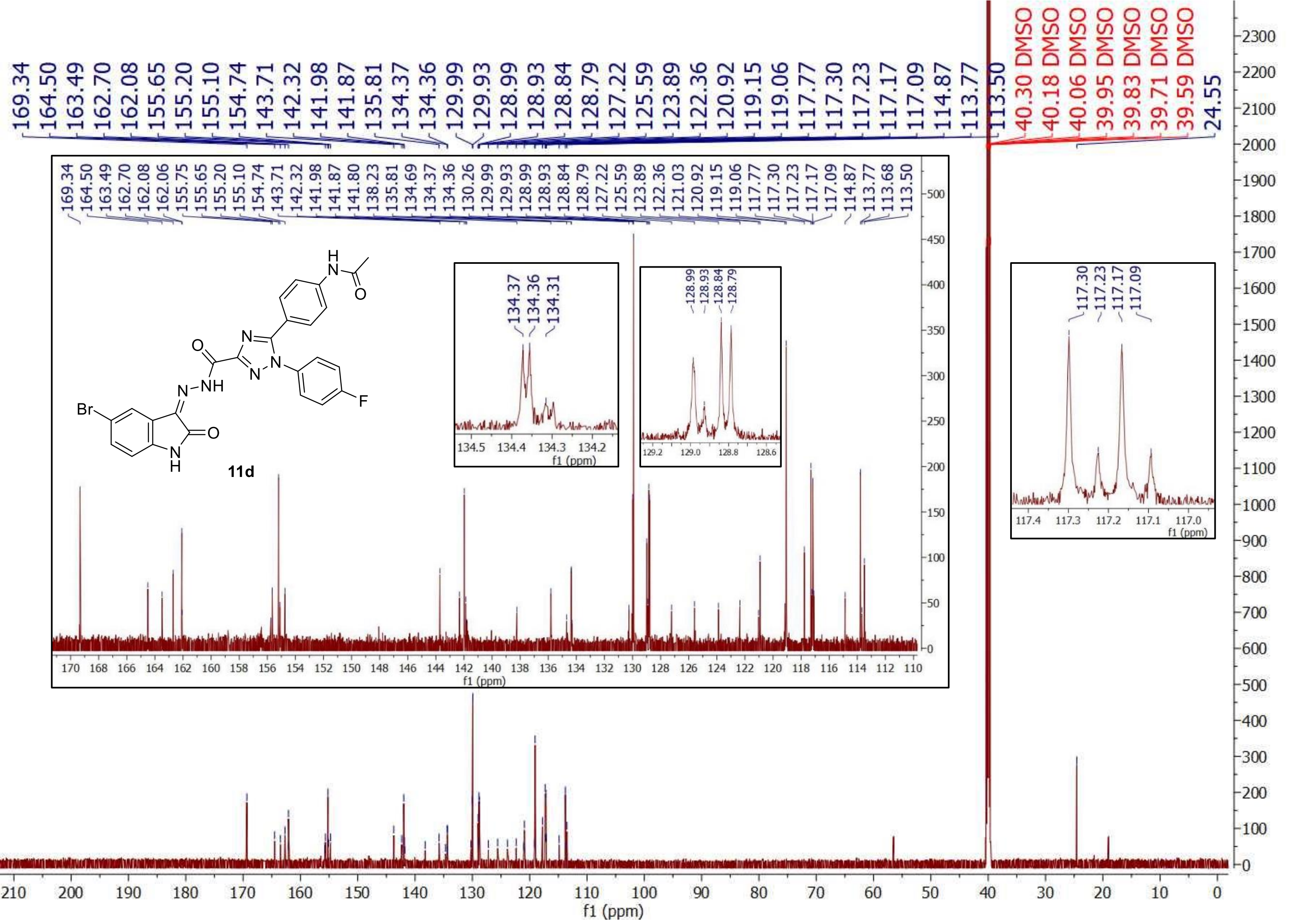

Br
N
NH
O
N
N
N
F
H
N
O
N
H
O
11d
f1 (ppm)

Compound Summary

Cpd	Name	Formula	RT	Mass	CAS	ID Source	Score	Score (Lib)	Score (DB)	Score (MFG)	Algorithm
1		C25 H17 Br F N7 O3	0.774	561.0564		FBF	99.73				FBF

Compound Details

Cpd. 1: C25 H17 Br F N7 O3

Name	Formula	RT	RI	Mass	Score	Algorithm	Lib/DB
	C25 H17 Br F N7 O3	0.774		561.0564	99.73	FBF	

Species	m/z	Score (Lib)	Num Spectra	Score (DB)	Score (MFG)	Score (RT)
(M+H)+ (M+Na)+	562.0636 584.0455					

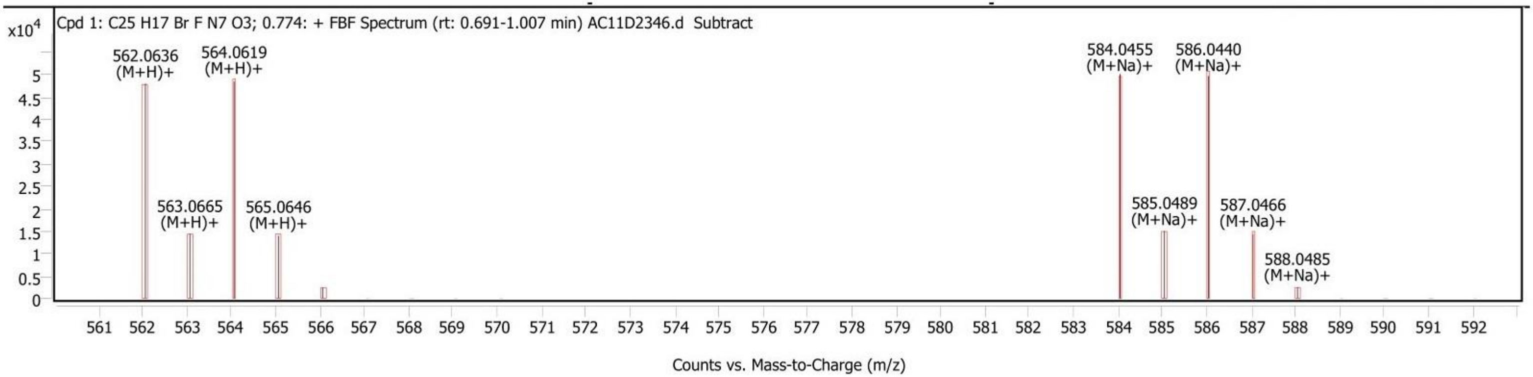

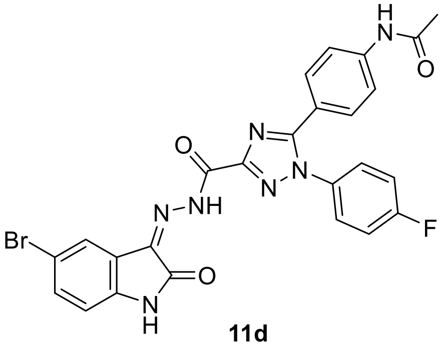

11d

Chemical Formula: $C_{25}H_{17}BrFN_7O_3$

Exact Mass: 561.0560

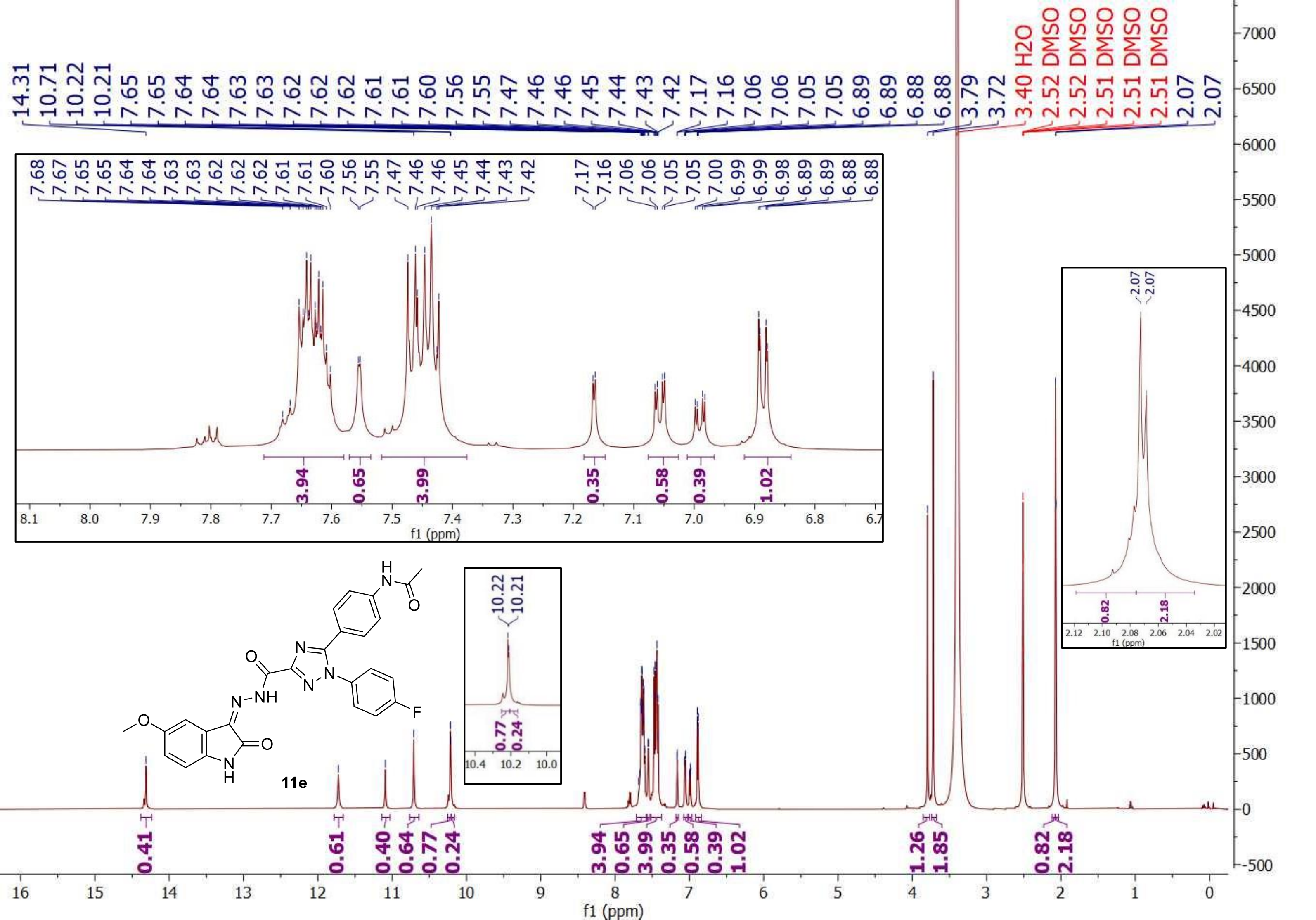

14.31 10.71 10.22 10.21 7.65 7.65 7.64 7.64 7.63 7.63 7.62 7.62 7.61 7.61 7.60 7.56 7.55 7.47 7.46 7.46 7.45 7.44 7.43 7.42 7.17 7.16 7.06 7.06 7.05 7.05 6.89 6.89 6.88 6.88 3.79 3.72
3.40 H2O 2.52 DMSO 2.52 DMSO 2.51 DMSO 2.51 DMSO 2.51 DMSO
2.07 2.07
11e
0.41 0.61 0.40 0.64 0.77 0.24 3.94 0.65 3.99 0.35 0.58 0.39 1.02 1.26 1.85 0.82 2.18
f1 (ppm)

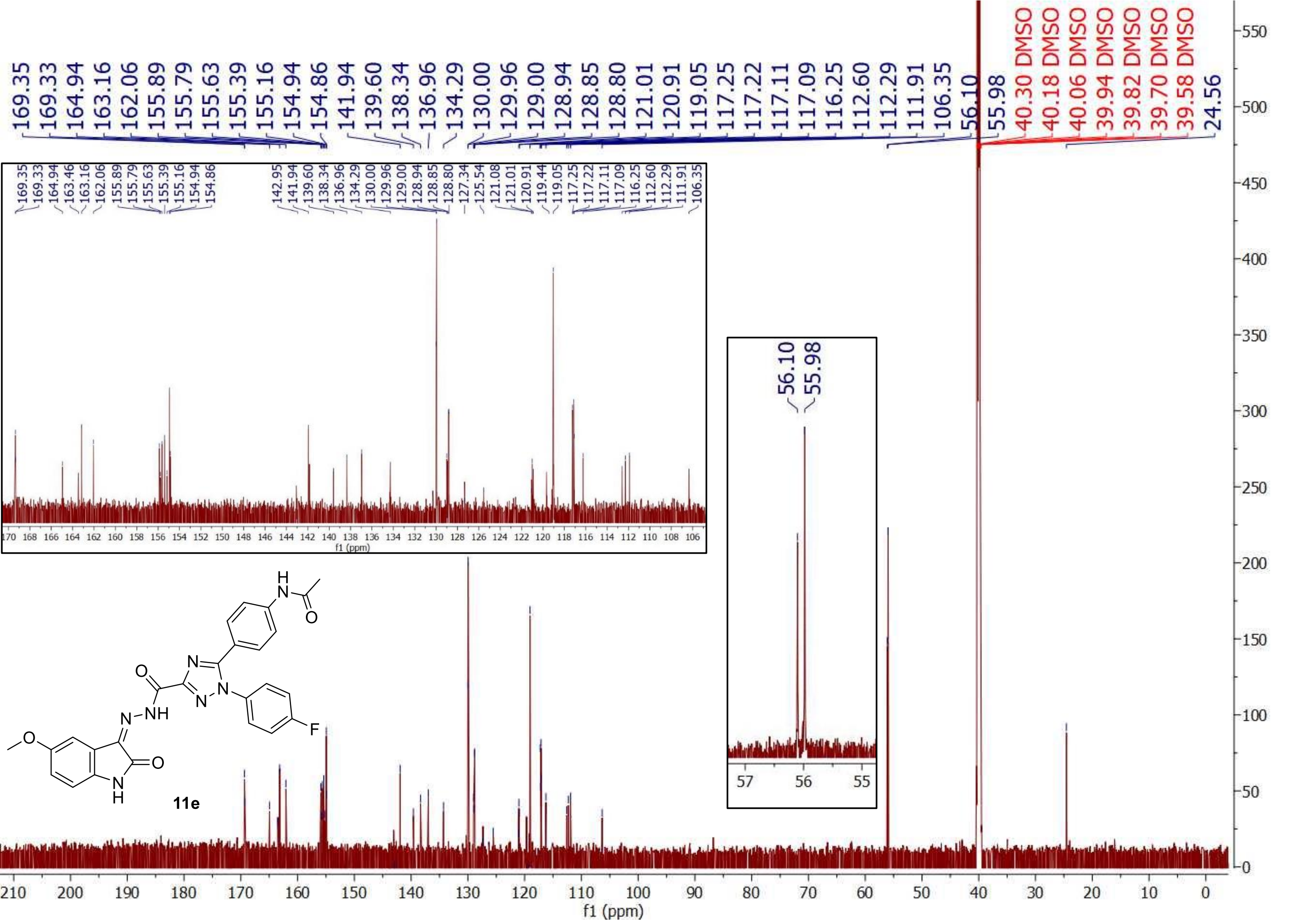
169.35
169.33
164.94
163.16
162.06
155.89
155.79
155.63
155.39
155.16
154.94
154.86
141.94
139.60
138.34
136.96
134.29
130.00
129.96
129.00
128.94
128.85
128.80
121.01
120.91
119.05
117.25
117.22
117.11
117.09
116.25
112.60
112.29
111.91
106.35
56.10
55.98
40.30 DMSO
40.18 DMSO
40.06 DMSO
39.94 DMSO
39.82 DMSO
39.70 DMSO
39.58 DMSO
24.56
169.35 169.33 164.94 163.46 163.16 162.06 155.89 155.79 155.63 155.39 155.16 154.94 154.86 142.95 141.94 139.60 138.34 136.96 134.29 130.00 129.96 129.00 128.94 128.85 128.80 127.34 125.54 121.08 121.01 120.91 119.44 119.05 117.25 117.22 117.11 117.09 116.25 112.60 112.29 111.91 106.35
f1 (ppm)
56.10
55.98
57 56 55
11e
f1 (ppm)

Compound Summary

Cpd	Name	Formula	RT	Mass	CAS	ID Source	Score	Score (Lib)	Score (DB)	Score (MFG)	Algorithm
1		C26 H20 F N7 O4	0.763	513.1566		FBF	99.45				FBF

Compound Details

Cpd. 1: C26 H20 F N7 O4

Name	Formula	RT	RI	Mass	Score	Algorithm	Lib/DB
	C26 H20 F N7 O4	0.763		513.1566	99.45	FBF	

Species	m/z	Score (Lib)	Num Spectra	Score (DB)	Score (MFG)	Score (RT)
(M+H)+ (M+Na)+	514.1639 536.1458					

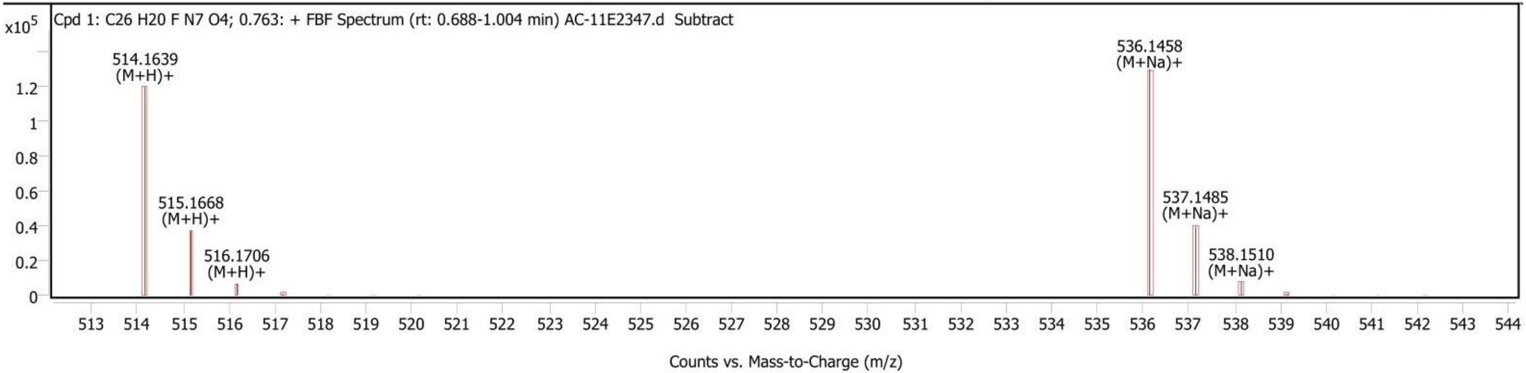

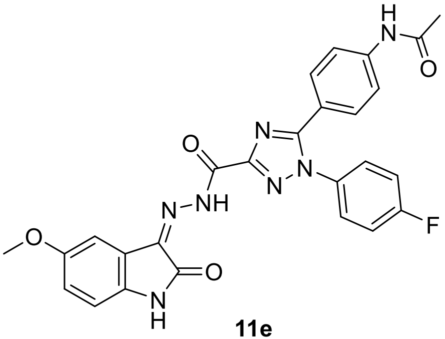

11e

Chemical Formula: $C_{26}H_{20}FN_7O_4$

Exact Mass: 513.1561

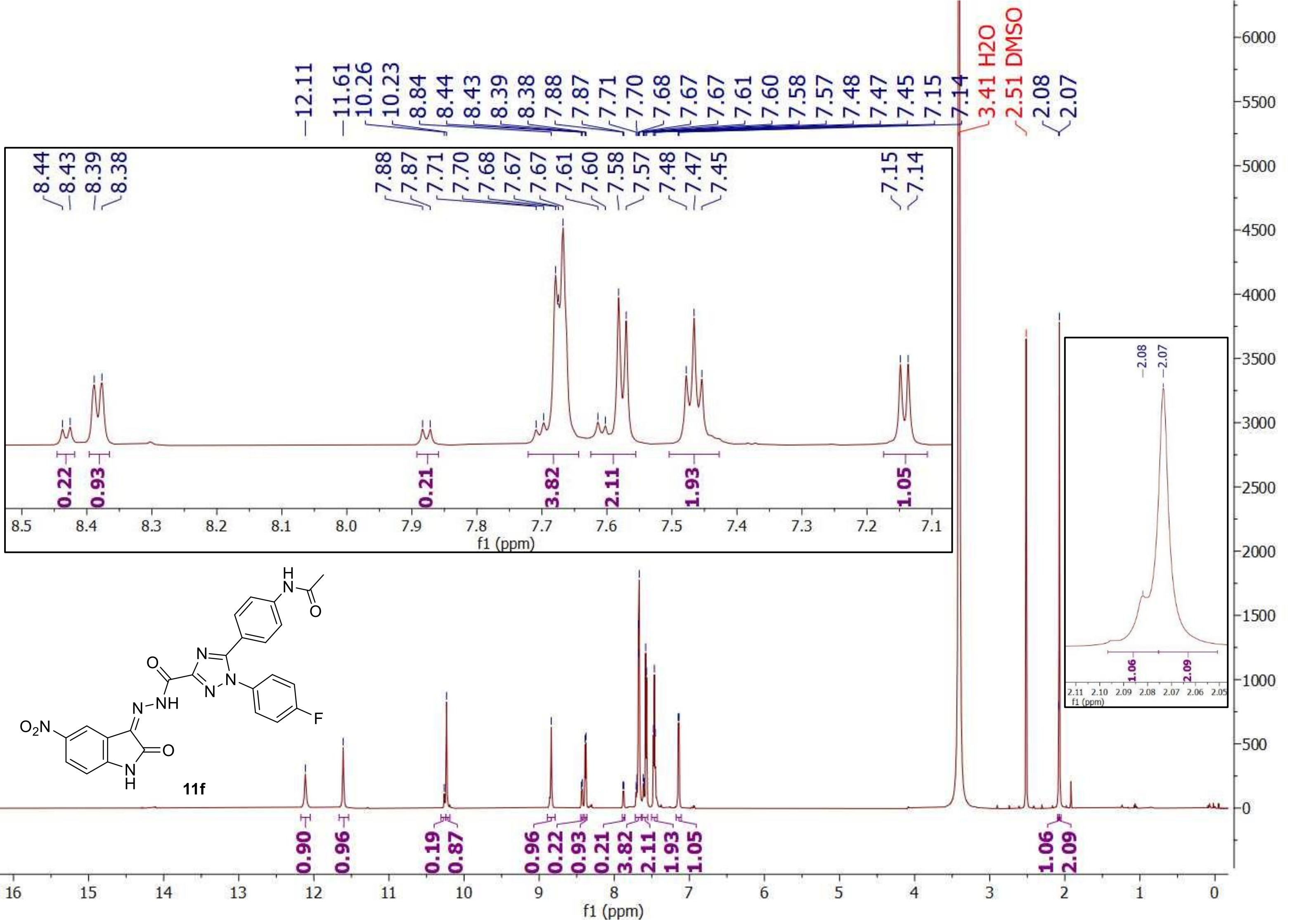
12.11
11.61
10.26
10.23
8.84
8.44
8.43
8.39
8.38
7.88
7.87
7.71
7.70
7.68
7.67
7.67
7.61
7.60
7.58
7.57
7.48
7.47
7.45
7.15
7.14
3.41 H2O
2.51 DMSO
2.08
2.07
0.22
0.93
0.21
3.82
2.11
1.93
1.05
f1 (ppm)
1.06
2.09
0.90
0.96
0.19
0.87
0.96
O$_2$N
NH
N
O
H
N
F
11f

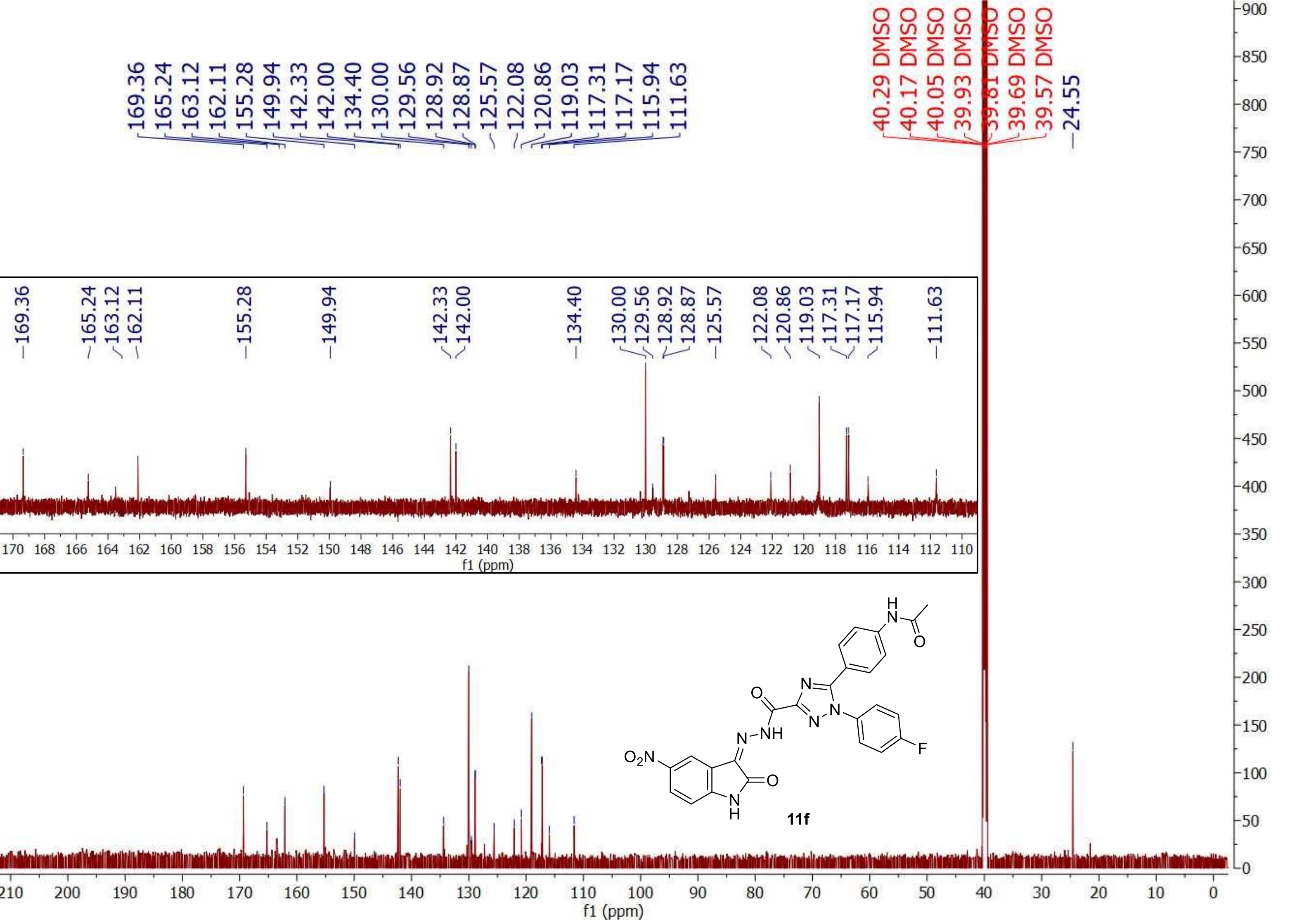

169.36
165.24
163.12
162.11
155.28
149.94
142.33
142.00
134.40
130.00
129.56
128.92
128.87
125.57
122.08
120.86
119.03
117.31
117.17
115.94
111.63
40.29 DMSO
40.17 DMSO
40.05 DMSO
39.93 DMSO
39.81 DMSO
39.69 DMSO
39.57 DMSO
24.55
f1 (ppm)
f1 (ppm)
O_2N
11f

Compound Summary

Cpd	Name	Formula	RT	Mass	CAS	ID Source	Score	Score (Lib)	Score (DB)	Score (MFG)	Algorithm
1		C25 H17 F N8 O5	0.775	528.1310		FBF	99.47				FBF

Compound Details

Cpd. 1: C25 H17 F N8 O5

Name	Formula	RT	RI	Mass	Score	Algorithm	Lib/DB
	C25 H17 F N8 O5	0.775		528.1310	99.47	FBF	

Species	m/z	Score (Lib)	Num Spectra	Score (DB)	Score (MFG)	Score (RT)
(M+H)+ (M+Na)+	529.1384 551.1200					

Compound Spectra

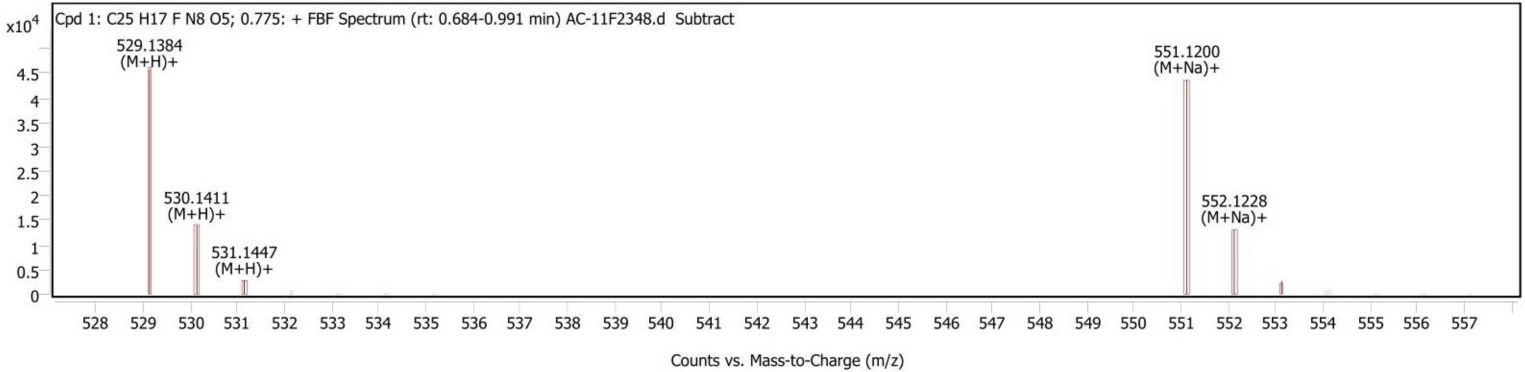

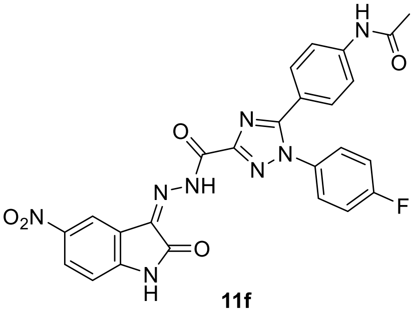

11f

Chemical Formula: $C_{25}H_{17}FN_8O_5$

Exact Mass: 528.1306

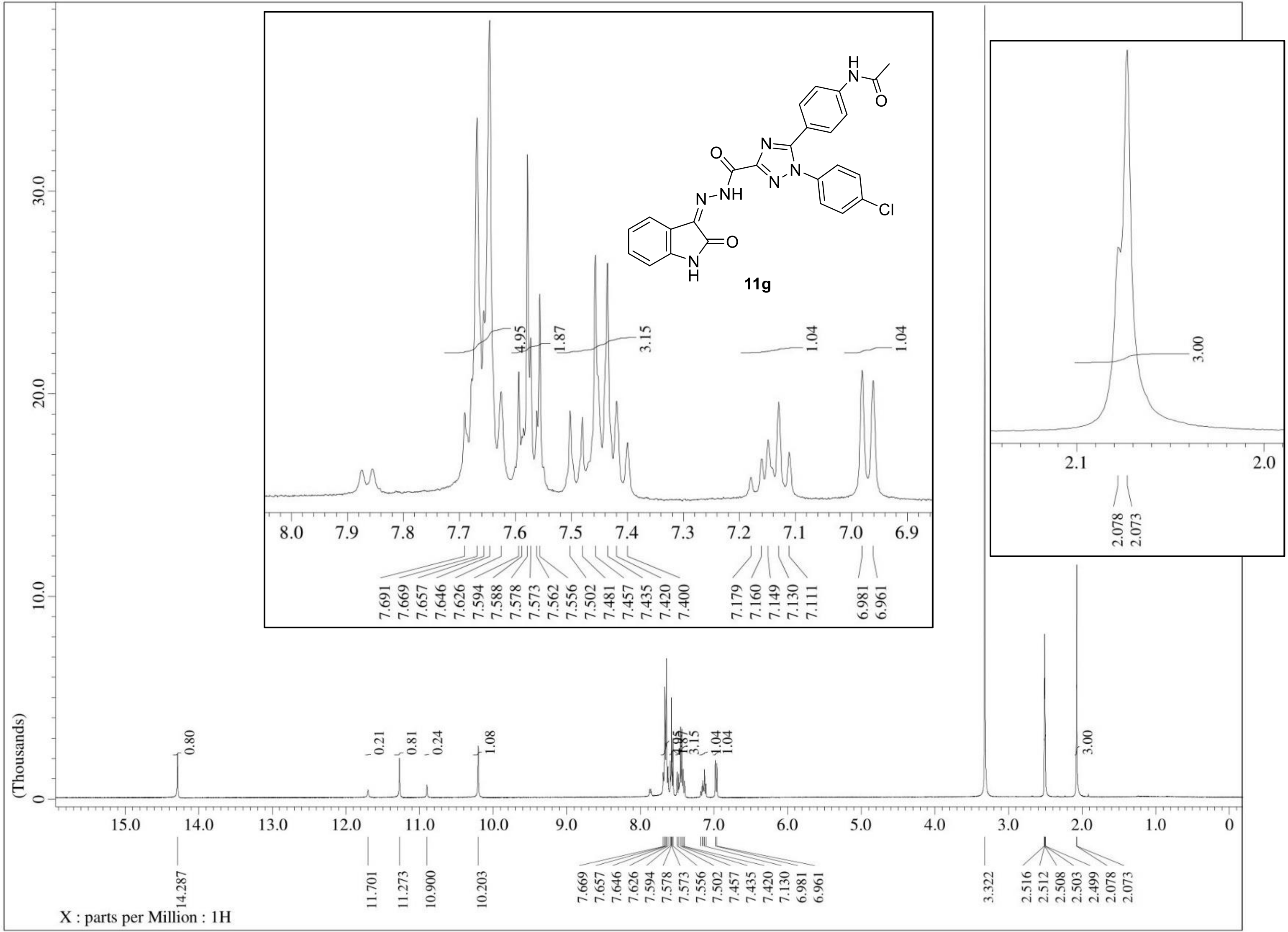

11g
H
N
O
O
N
N
NH
N
N
Cl
O
N
H
(Thousands)
30.0
20.0
10.0
0
0.80
0.21
0.81
0.24
1.08
4.95
1.87
3.15
1.04
1.04
3.00
15.0
14.0
13.0
12.0
11.0
10.0
9.0
8.0
7.0
6.0
5.0
4.0
3.0
2.0
1.0
0
14.287
11.701
11.273
10.900
10.203
7.669
7.657
7.646
7.626
7.594
7.578
7.573
7.556
7.502
7.457
7.435
7.420
7.130
6.981
6.961
3.322
2.516
2.512
2.508
2.503
2.499
2.078
2.073
X : parts per Million : 1H
8.0
7.9
7.8
7.7
7.6
7.5
7.4
7.3
7.2
7.1
7.0
6.9
7.691
7.669
7.657
7.646
7.626
7.594
7.588
7.578
7.573
7.562
7.556
7.502
7.481
7.457
7.435
7.420
7.400
7.179
7.160
7.149
7.130
7.111
6.981
6.961
2.1
2.0
2.078
2.073

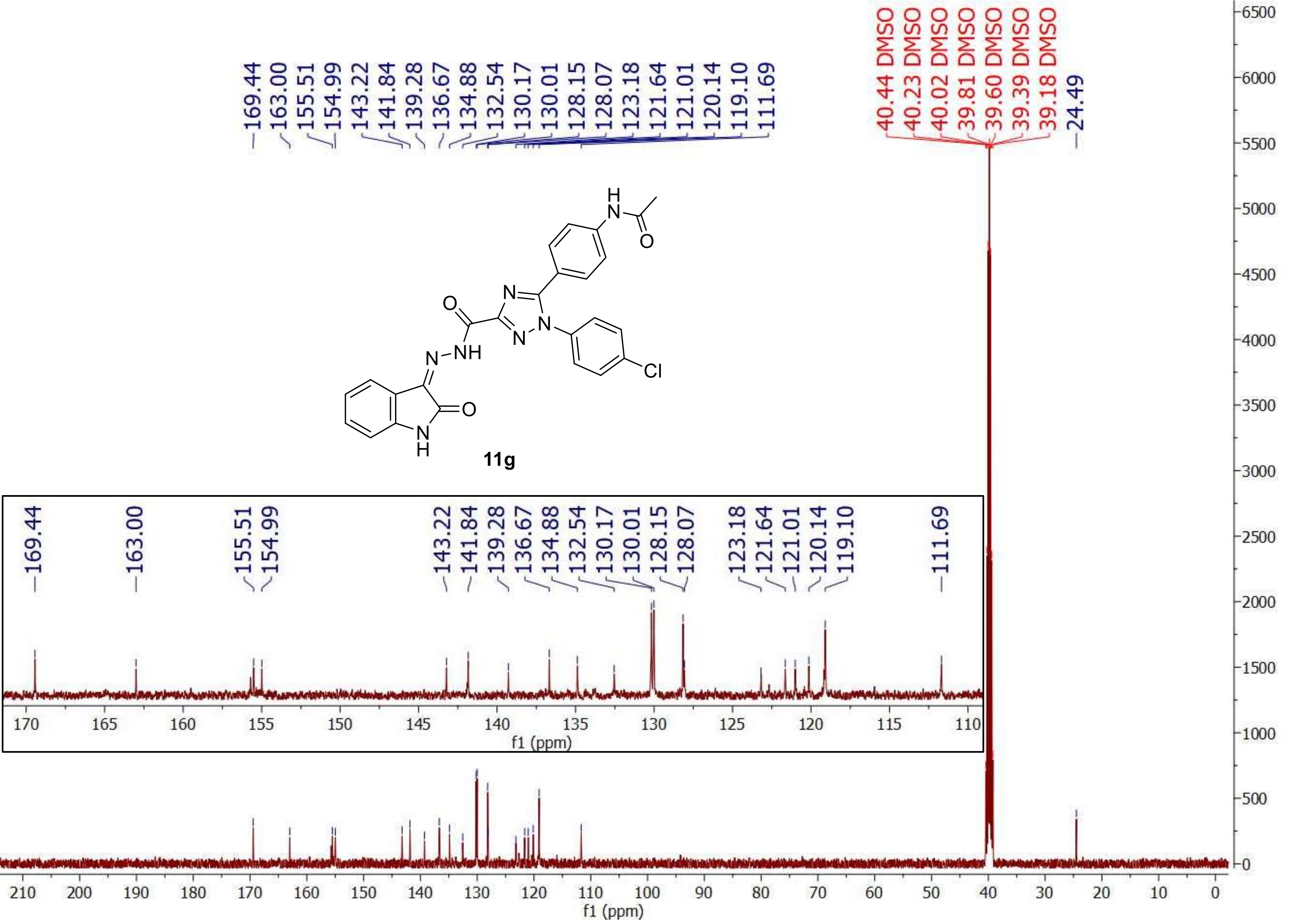
169.44
163.00
155.51
154.99
143.22
141.84
139.28
136.67
134.88
132.54
130.17
130.01
128.15
128.07
123.18
121.64
121.01
120.14
119.10
111.69
40.44 DMSO
40.23 DMSO
40.02 DMSO
39.81 DMSO
39.60 DMSO
39.39 DMSO
39.18 DMSO
24.49
H
N
O
N
N
N
O
NH
N
Cl
O
N
H
11g
f1 (ppm)
f1 (ppm)

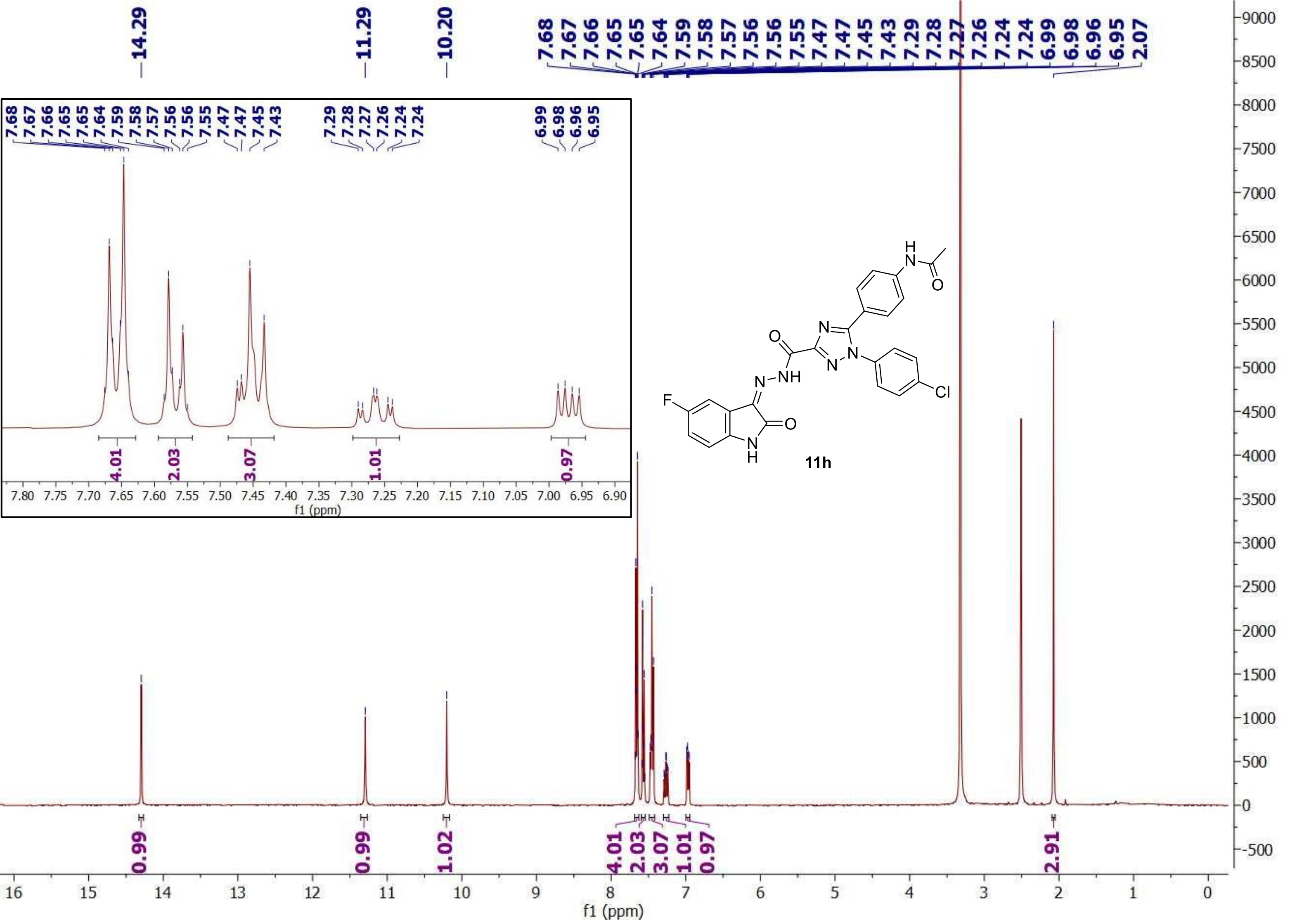

14.29
11.29
10.20
7.68 7.67 7.66 7.65 7.65 7.64 7.59 7.58 7.57 7.56 7.56 7.55 7.47 7.47 7.45 7.43 7.29 7.28 7.27 7.26 7.24 7.24 6.99 6.98 6.96 6.95 2.07
4.01 2.03 3.07 1.01 0.97
f1 (ppm)
F
N
NH
O
N
N
N
H
Cl
11h
0.99 0.99 1.02 4.01 2.03 3.07 1.01 0.97 2.91
f1 (ppm)

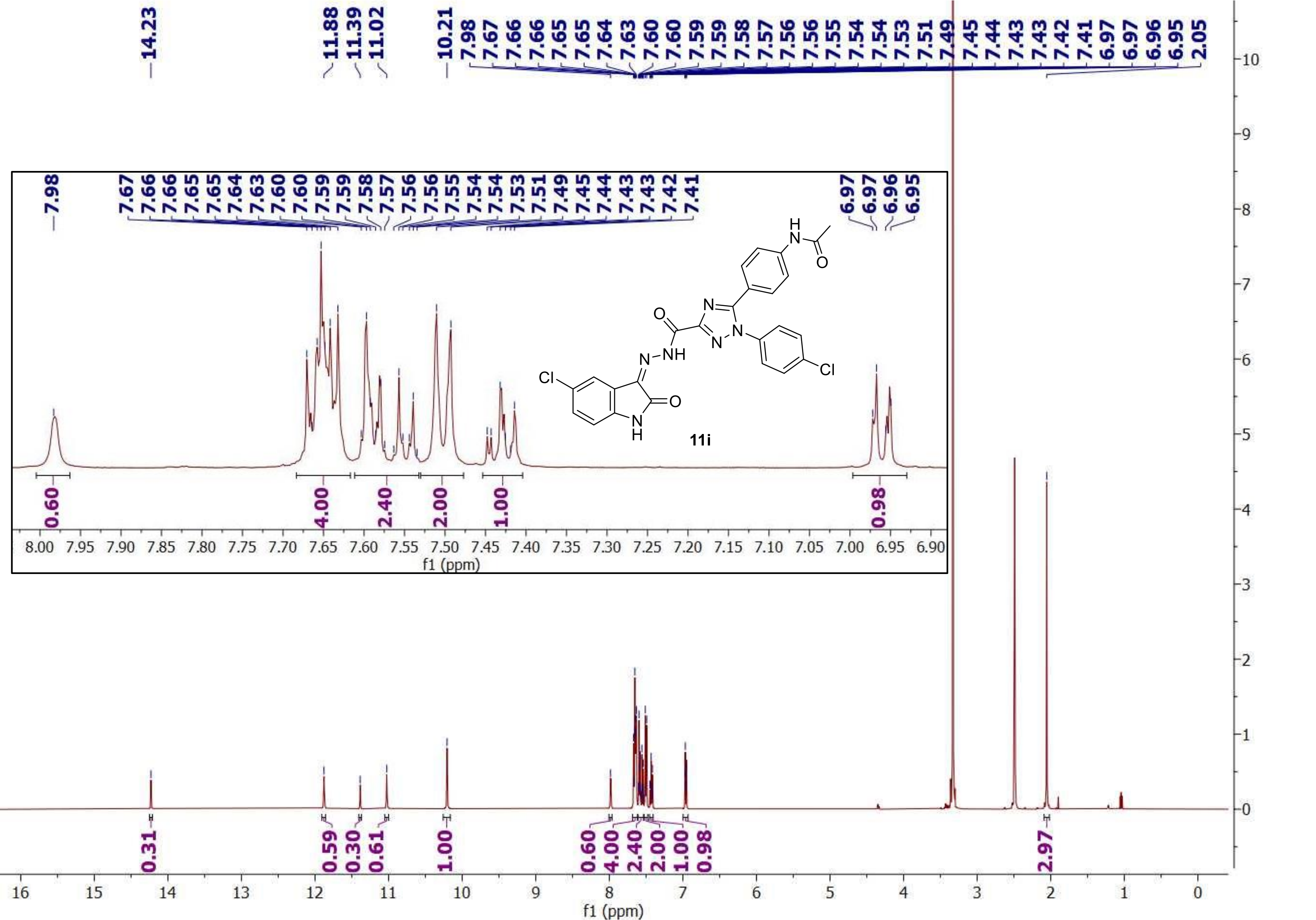
14.23
11.88
11.39
11.02
10.21
7.98
7.67
7.66
7.66
7.65
7.65
7.64
7.63
7.60
7.60
7.59
7.59
7.58
7.57
7.56
7.56
7.55
7.54
7.54
7.53
7.51
7.49
7.45
7.44
7.43
7.43
7.42
7.41
6.97
6.97
6.96
6.95
2.05
0.60
4.00
2.40
2.00
1.00
0.98
f1 (ppm)
11i
0.31
0.59
0.30
0.61
1.00
0.60
4.00
2.40
2.00
1.00
0.98
2.97
f1 (ppm)

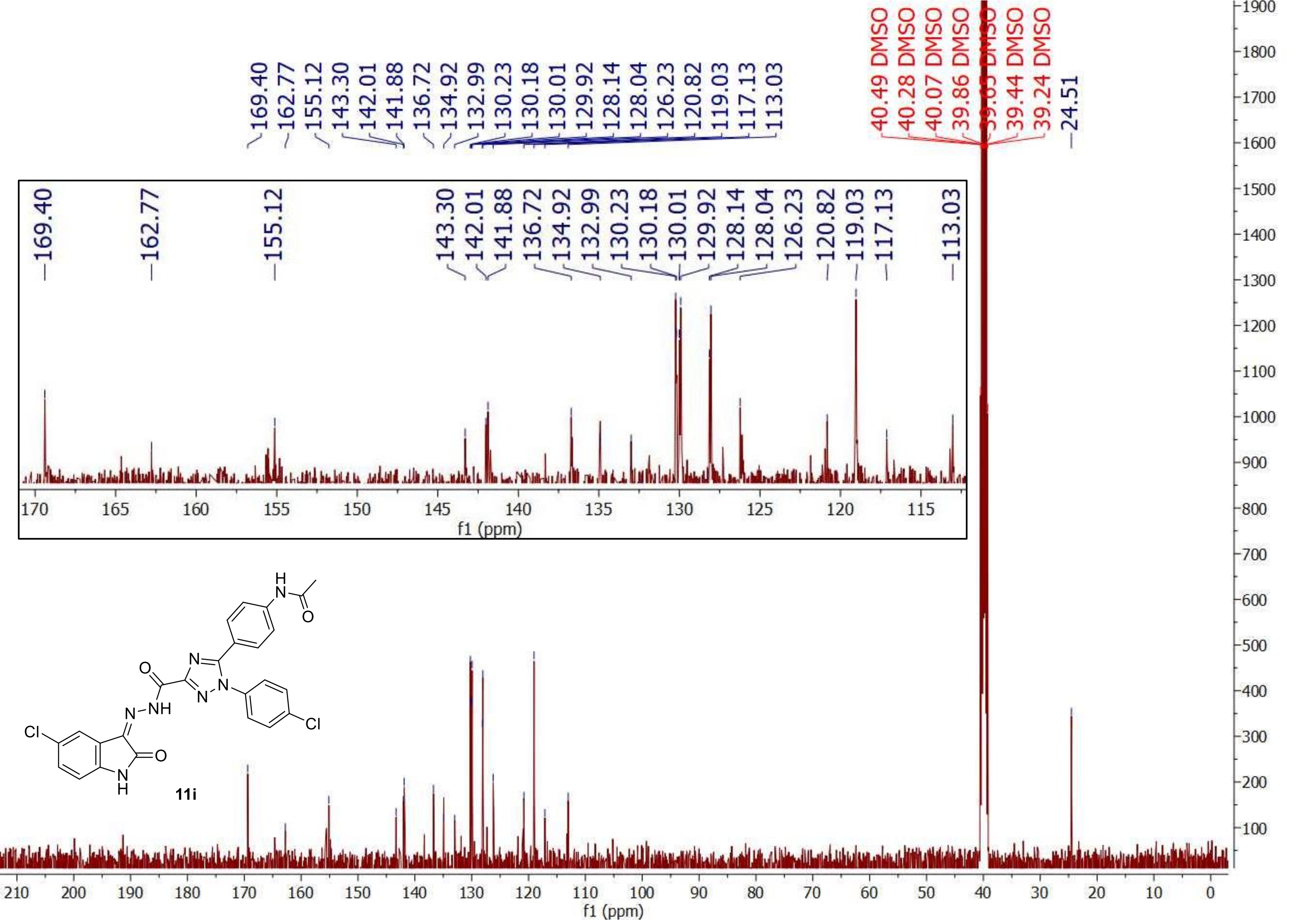
169.40
162.77
155.12
143.30
142.01
141.88
136.72
134.92
132.99
130.23
130.18
130.01
129.92
128.14
128.04
126.23
120.82
119.03
117.13
113.03
40.49 DMSO
40.28 DMSO
40.07 DMSO
39.86 DMSO
39.65 DMSO
39.44 DMSO
39.24 DMSO
24.51
f1 (ppm)
11i

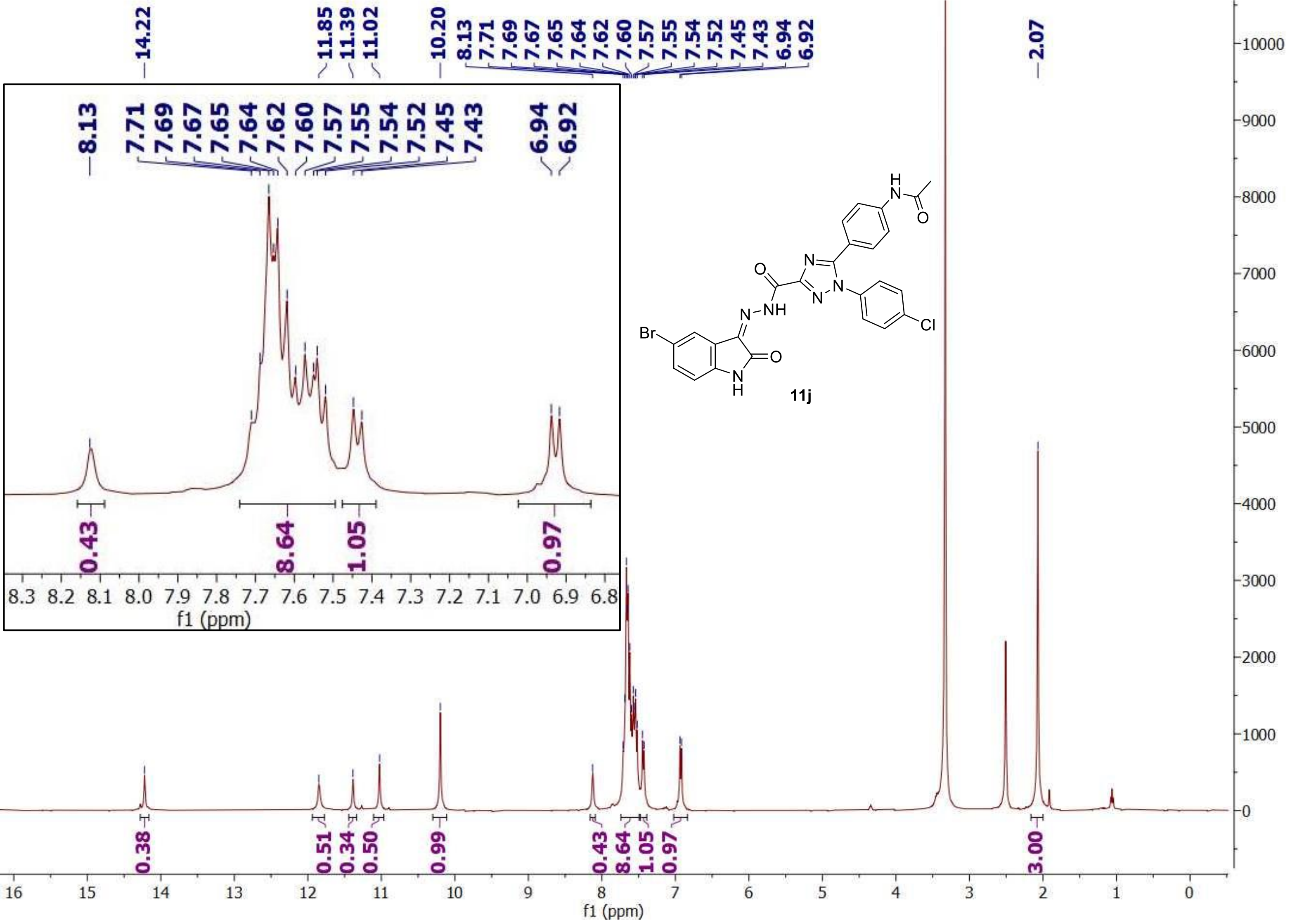

14.22
11.85
11.39
11.02
10.20
8.13
7.71
7.69
7.67
7.65
7.64
7.62
7.60
7.57
7.55
7.54
7.52
7.45
7.43
6.94
6.92
2.07
0.43
8.64
1.05
0.97
8.3 8.2 8.1 8.0 7.9 7.8 7.7 7.6 7.5 7.4 7.3 7.2 7.1 7.0 6.9 6.8
f1 (ppm)
Br
N
NH
O
N
N
N
H
Cl
NH
O
11j
0.38
0.51
0.34
0.50
0.99
3.00
16 15 14 13 12 11 10 9 8 7 6 5 4 3 2 1 0
f1 (ppm)
10000
9000
8000
7000
6000
5000
4000
3000
2000
1000
0

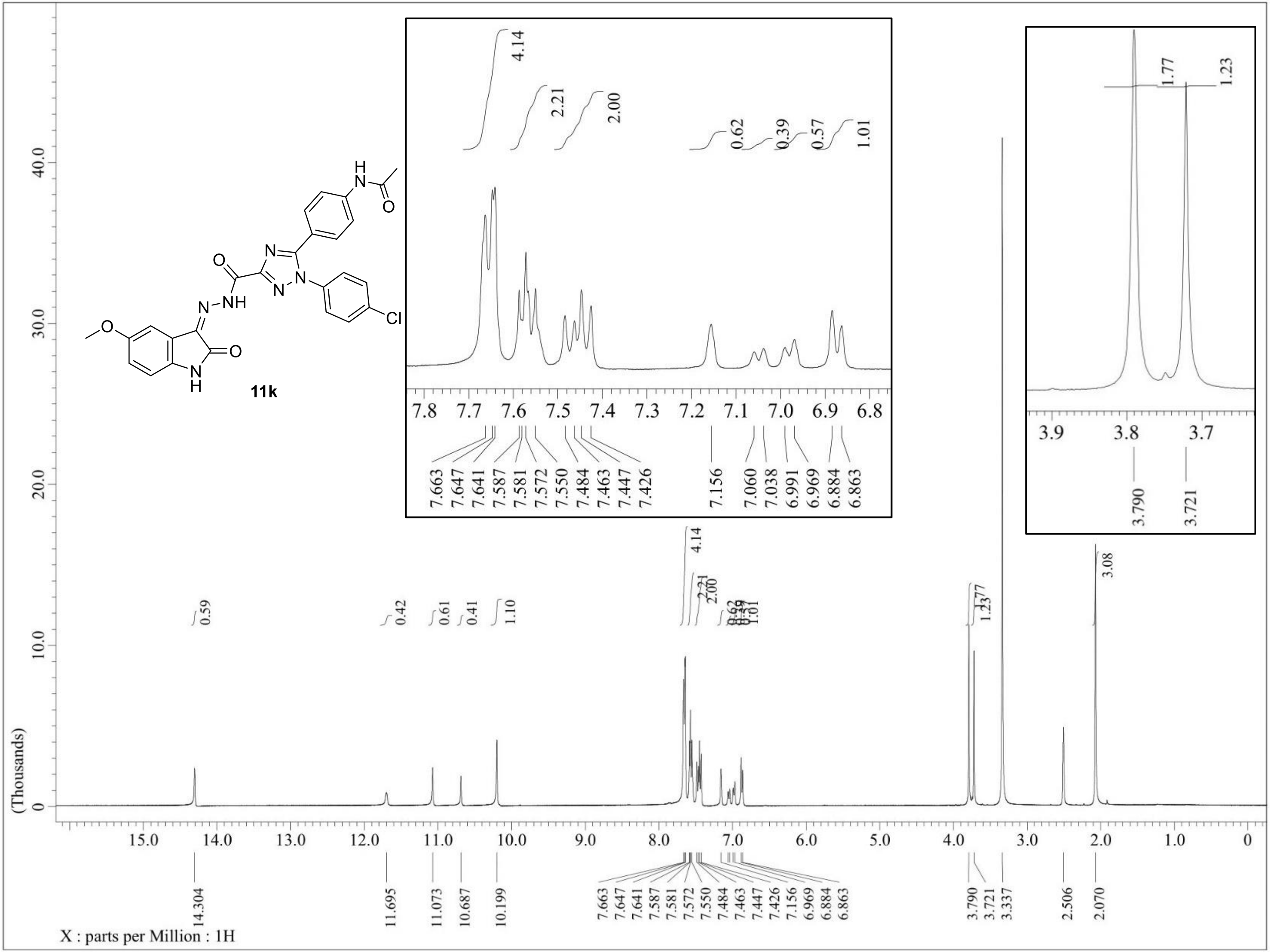

11k
Cl
O
N
NH
N–NH
H
(Thousands)
0
10.0
20.0
30.0
40.0
15.0
14.0
13.0
12.0
11.0
10.0
9.0
8.0
7.0
6.0
5.0
4.0
3.0
2.0
1.0
0
X : parts per Million : 1H
0.59
0.42
0.61
0.41
1.10
4.14
2.21
2.00
0.62
0.39
0.57
1.01
1.77
1.23
3.08
14.304
11.695
11.073
10.687
10.199
7.663
7.647
7.641
7.587
7.581
7.572
7.550
7.484
7.463
7.447
7.426
7.156
7.060
7.038
6.991
6.969
6.884
6.863
3.790
3.721
3.337
2.506
2.070
7.8
7.7
7.6
7.5
7.4
7.3
7.2
7.1
7.0
6.9
6.8
3.9
3.8
3.7

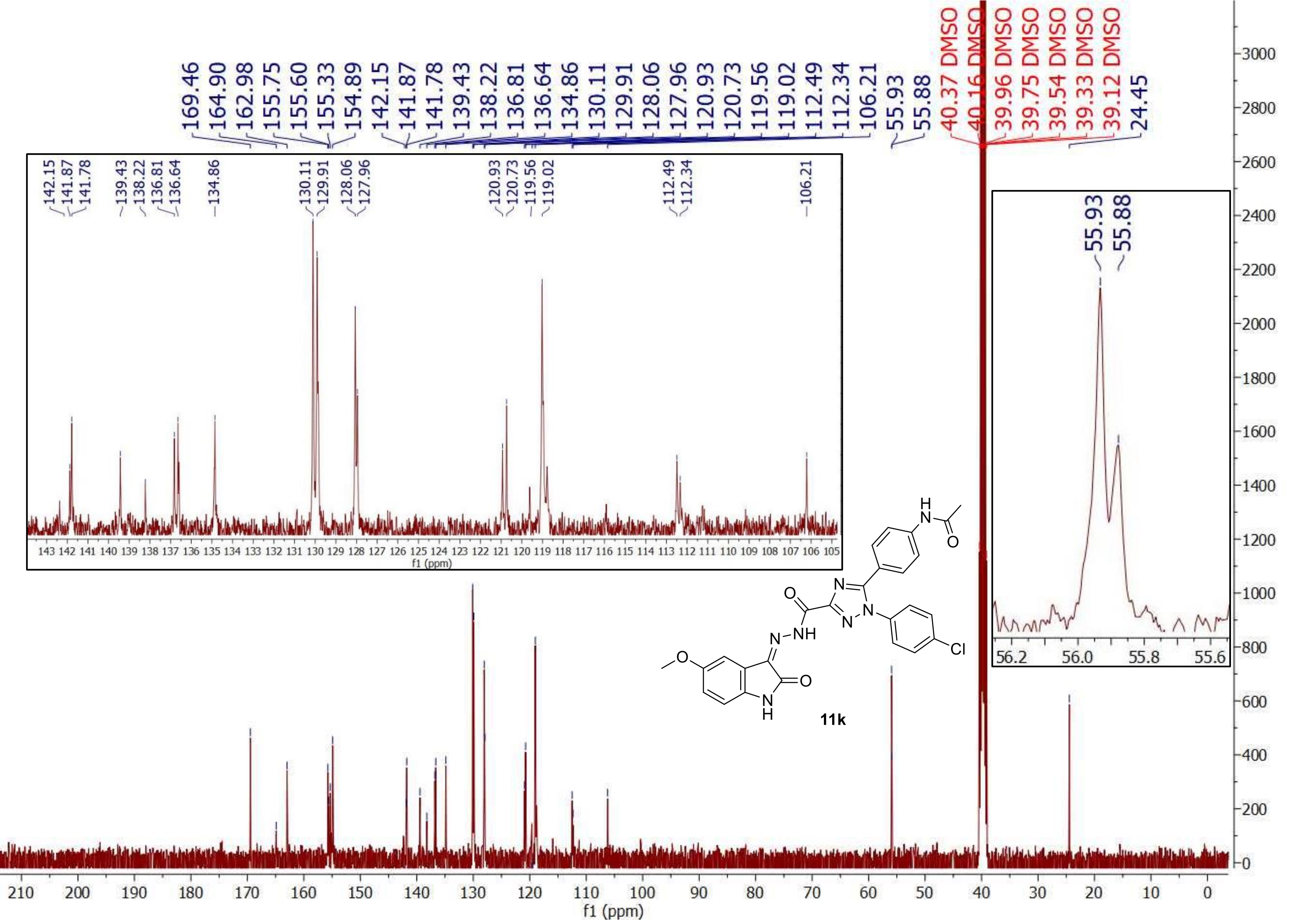
169.46
164.90
162.98
155.75
155.60
155.33
154.89
142.15
141.87
141.78
139.43
138.22
136.81
136.64
134.86
130.11
129.91
128.06
127.96
120.93
120.73
119.56
119.02
112.49
112.34
106.21
55.93
55.88
40.37 DMSO
40.16 DMSO
39.96 DMSO
39.75 DMSO
39.54 DMSO
39.33 DMSO
39.12 DMSO
24.45
f1 (ppm)
H
N
O
N
N
N
O
N–NH
O
Cl
O
N
H

11k

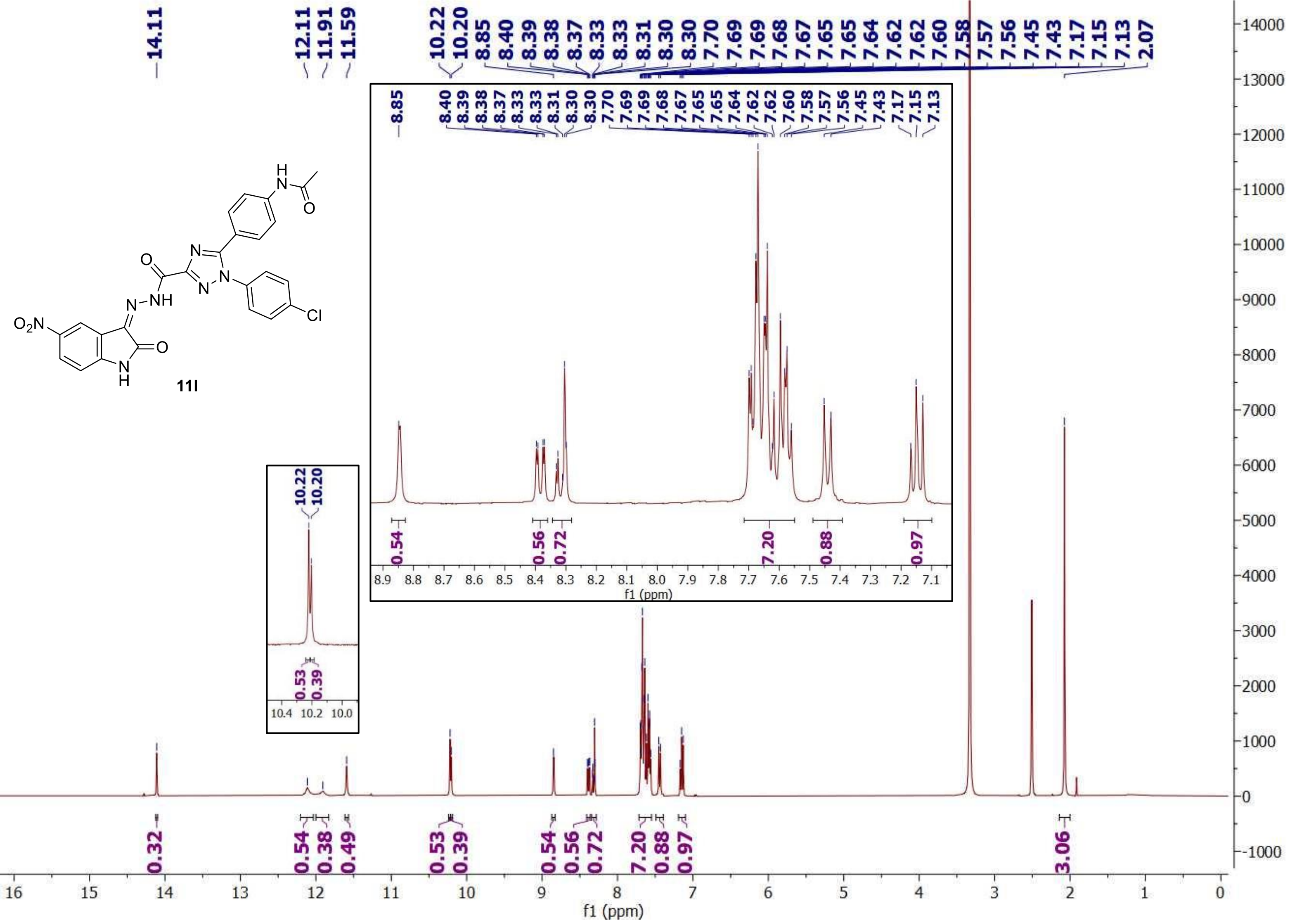

14.11
12.11
11.91
11.59
10.22
10.20
8.85
8.40
8.39
8.38
8.37
8.33
8.33
8.31
8.30
8.30
7.70
7.69
7.69
7.68
7.67
7.65
7.65
7.64
7.62
7.62
7.60
7.58
7.57
7.56
7.45
7.43
7.17
7.15
7.13
2.07
O2N
N–NH
O
N
N
N
H
N
O
Cl
11l
0.54
0.56
0.72
7.20
0.88
0.97
0.53
0.39
0.32
0.54
0.38
0.49
3.06
f1 (ppm)

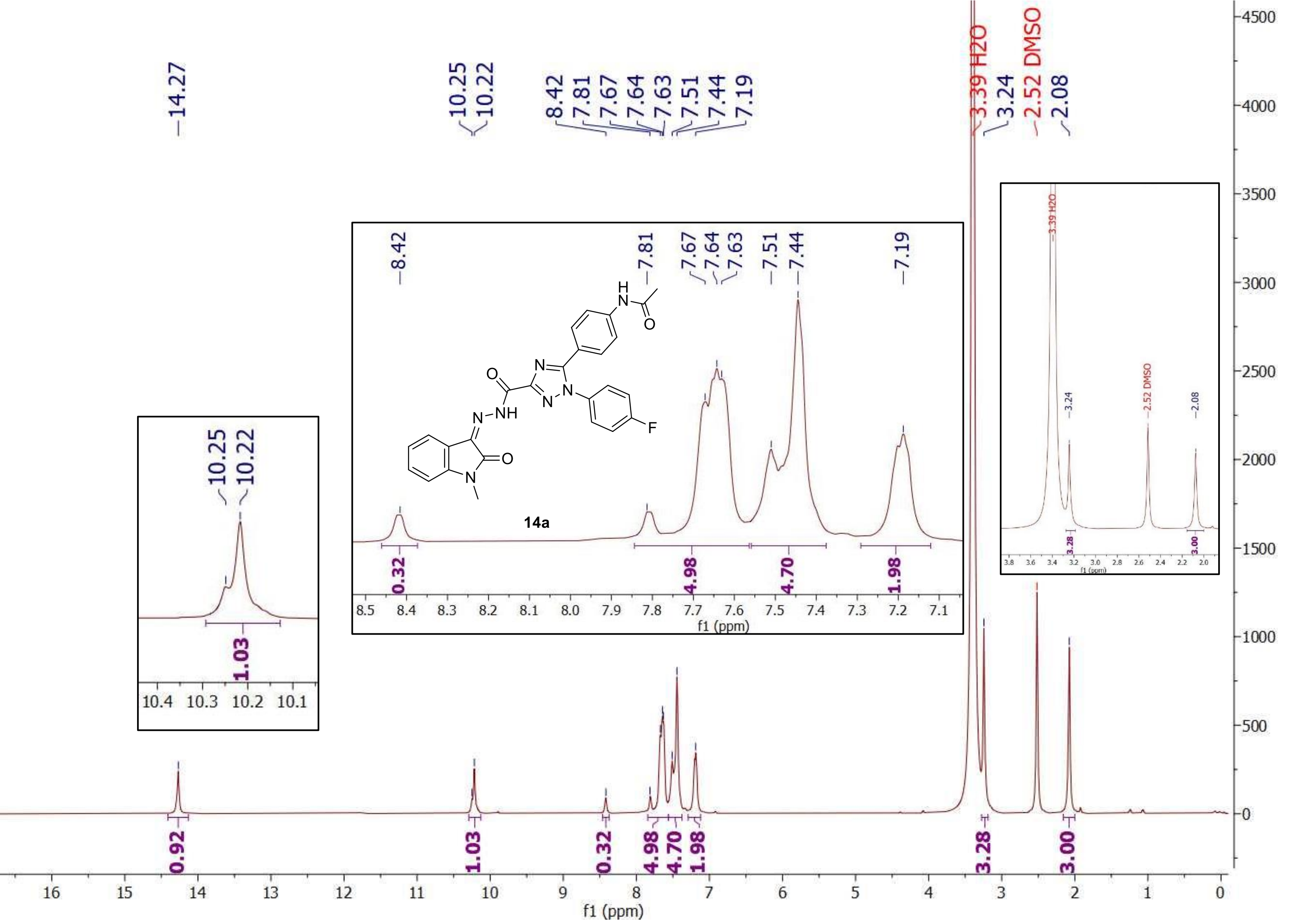

14.27
10.25
10.22
8.42
7.81
7.67
7.64
7.63
7.51
7.44
7.19
3.39 H2O
3.24
2.52 DMSO
2.08
14a
0.92
1.03
0.32
4.98
4.70
1.98
3.28
3.00
f1 (ppm)

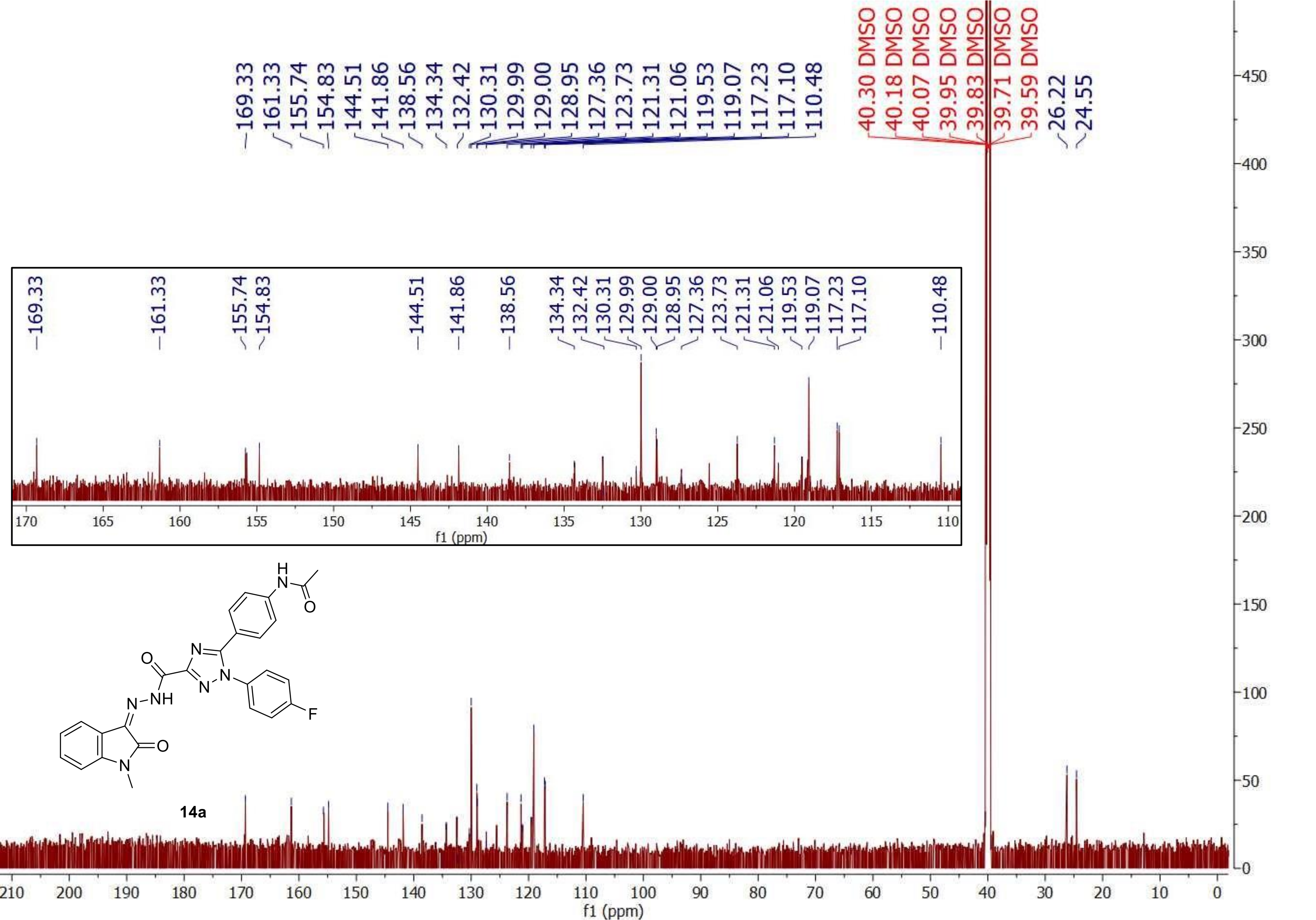
169.33
161.33
155.74
154.83
144.51
141.86
138.56
134.34
132.42
130.31
129.99
129.00
128.95
127.36
123.73
121.31
121.06
119.53
119.07
117.23
117.10
110.48
40.30 DMSO
40.18 DMSO
40.07 DMSO
39.95 DMSO
39.83 DMSO
39.71 DMSO
39.59 DMSO
26.22
24.55
f1 (ppm)
f1 (ppm)
14a

Compound Summary

Cpd	Name	Formula	RT	Mass	CAS	ID Source	Score	Score (Lib)	Score (DB)	Score (MFG)	Algorithm
1		C26 H20 F N7 O3	0.762	497.1620		FBF	98.98				FBF

Compound Details

Cpd. 1: C26 H20 F N7 O3

Name	Formula	RT	RI	Mass	Score	Algorithm	Lib/DB
	C26 H20 F N7 O3	0.762		497.1620	98.98	FBF	

Species	m/z	Score (Lib)	Num Spectra	Score (DB)	Score (MFG)	Score (RT)
(M+H)+ (M+Na)+	498.1691 520.1511					

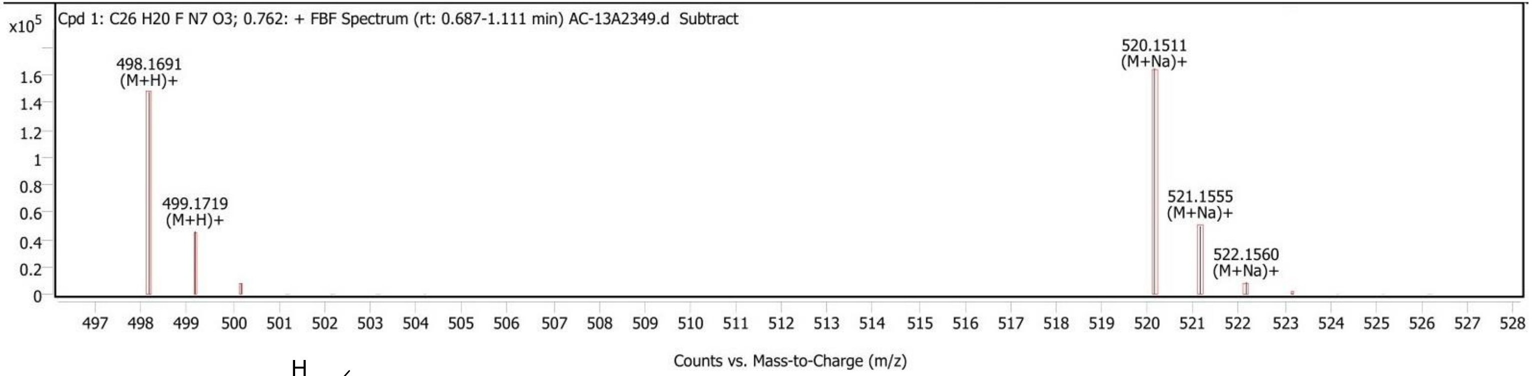

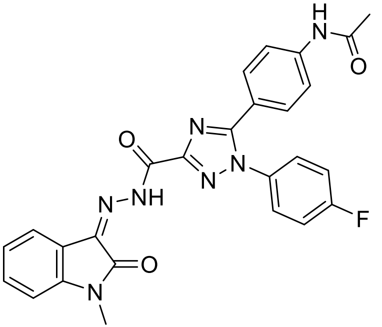

14a

Chemical Formula: $C_{26}H_{20}FN_7O_3$

Exact Mass: 497.1612

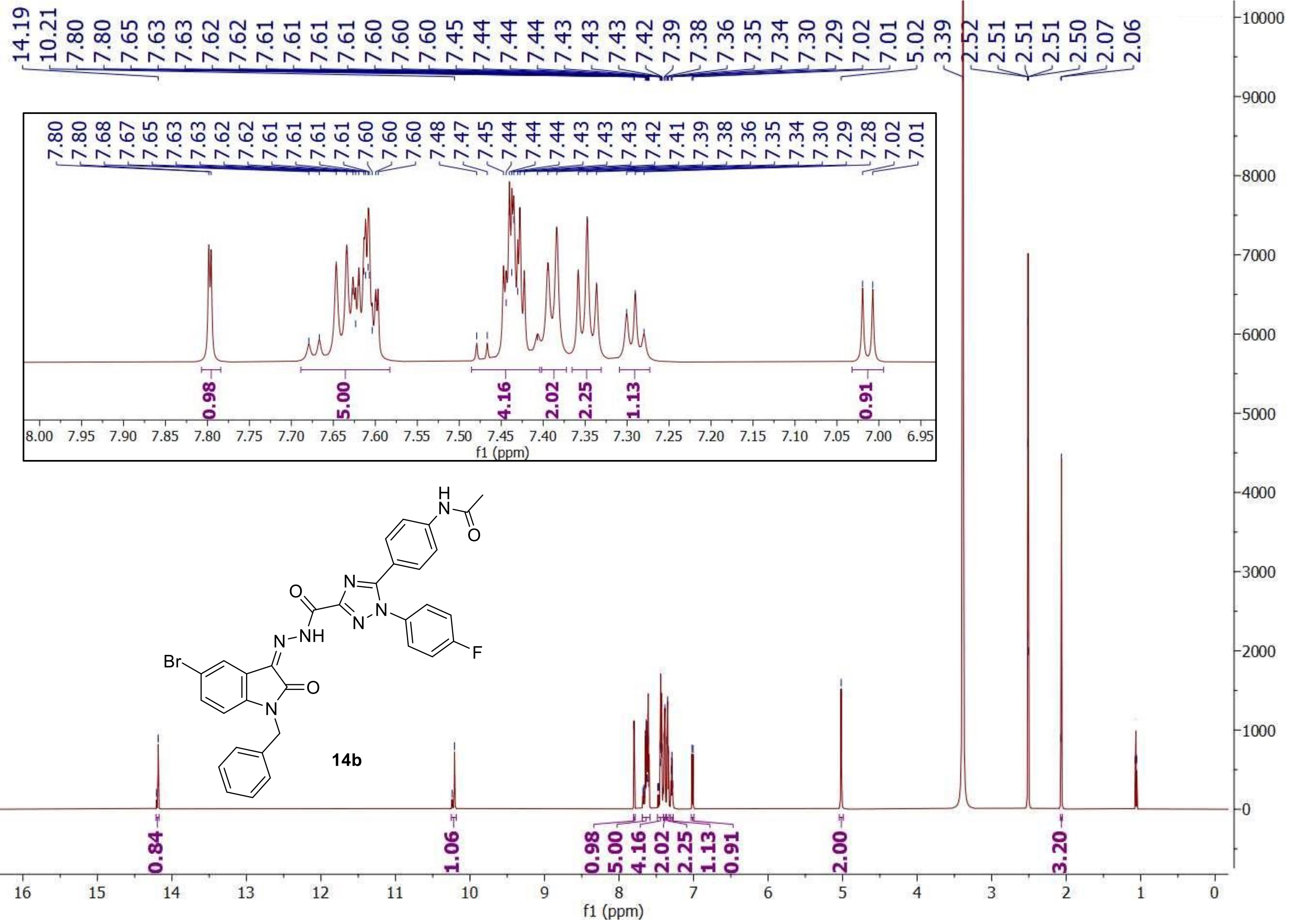

14.19 10.21 7.80 7.80 7.65 7.63 7.63 7.62 7.62 7.61 7.61 7.61 7.61 7.60 7.60 7.60 7.45 7.44 7.44 7.44 7.43 7.43 7.42 7.39 7.38 7.36 7.35 7.34 7.30 7.29 7.02 7.01 5.02 3.39 2.52 2.51 2.51 2.51 2.50 2.07 2.06
7.80 7.80 7.68 7.67 7.65 7.63 7.63 7.62 7.62 7.61 7.61 7.61 7.60 7.60 7.60 7.48 7.47 7.45 7.44 7.44 7.44 7.43 7.43 7.43 7.42 7.41 7.39 7.38 7.36 7.35 7.34 7.30 7.29 7.28 7.02 7.01
0.98 5.00 4.16 2.02 2.25 1.13 0.91
8.00 7.95 7.90 7.85 7.80 7.75 7.70 7.65 7.60 7.55 7.50 7.45 7.40 7.35 7.30 7.25 7.20 7.15 7.10 7.05 7.00 6.95
f1 (ppm)
Br
N
O
N
NH
O
N
N
N
F
H
N
O
14b
0.84 1.06 0.98 5.00 4.16 2.02 2.25 1.13 0.91 2.00 3.20
16 15 14 13 12 11 10 9 8 7 6 5 4 3 2 1 0
f1 (ppm)
10000 9000 8000 7000 6000 5000 4000 3000 2000 1000 0

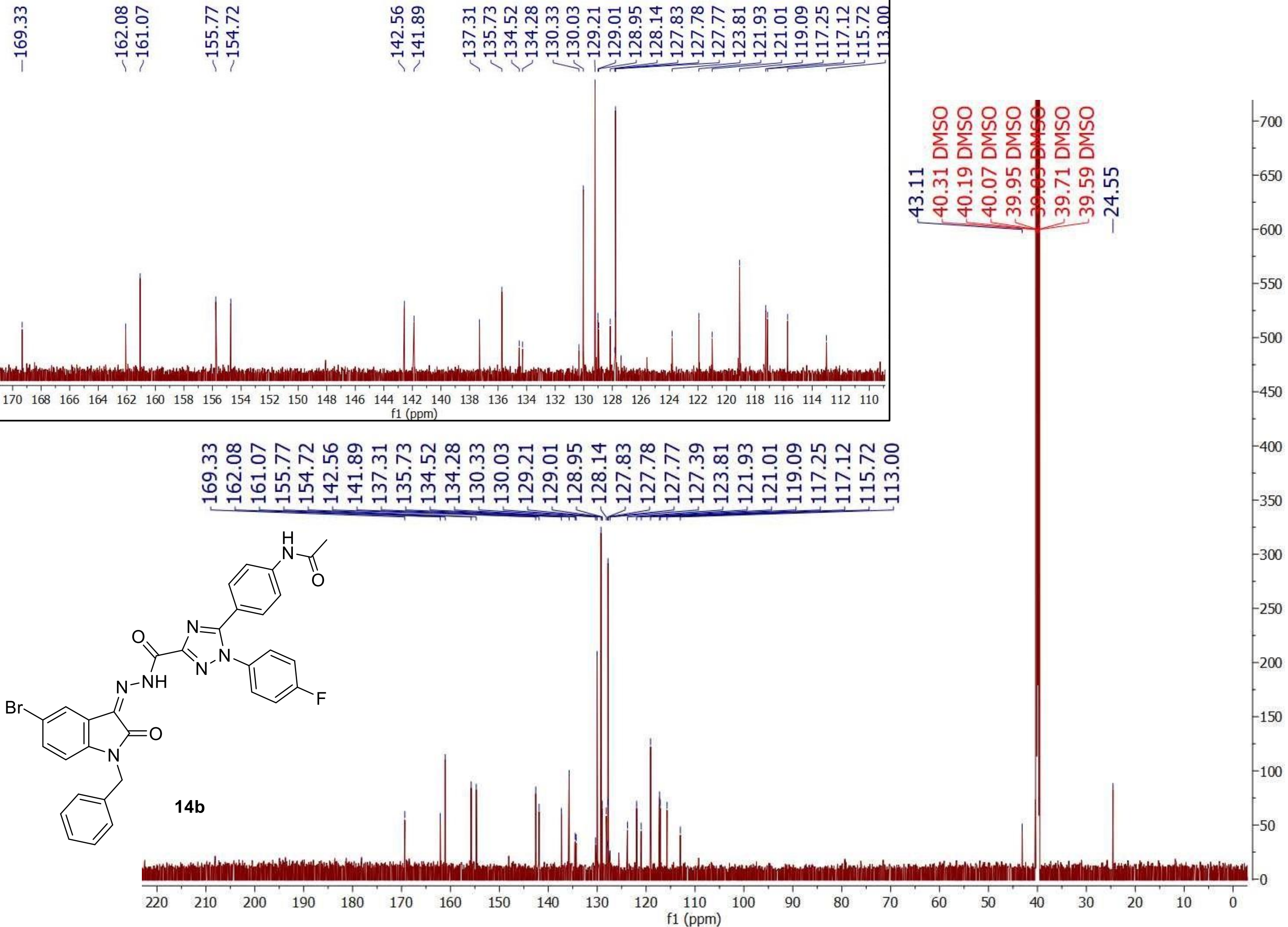
169.33
162.08
161.07
155.77
154.72
142.56
141.89
137.31
135.73
134.52
134.28
130.33
130.03
129.21
129.01
128.95
128.14
127.83
127.78
127.77
123.81
121.93
121.01
119.09
117.25
117.12
115.72
113.00
f1 (ppm)
43.11
40.31 DMSO
40.19 DMSO
40.07 DMSO
39.95 DMSO
39.83 DMSO
39.71 DMSO
39.59 DMSO
24.55
169.33
162.08
161.07
155.77
154.72
142.56
141.89
137.31
135.73
134.52
134.28
130.33
130.03
129.21
129.01
128.95
128.14
127.83
127.78
127.77
127.39
123.81
121.93
121.01
119.09
117.25
117.12
115.72
113.00
f1 (ppm)
14b

Item name: AC13B

Item name: AC13B, Sample position: 1:B,2, Replicate number: 1

.	Component name	Identification status	Formula	Neutral mass (Da)	Observed neutral mass (Da)	Observed m/z	Mass error (mDa)
1	AC13B	Identified	C32H23BrFO3N7	651.10298	651.1027	652.1100	-0.3

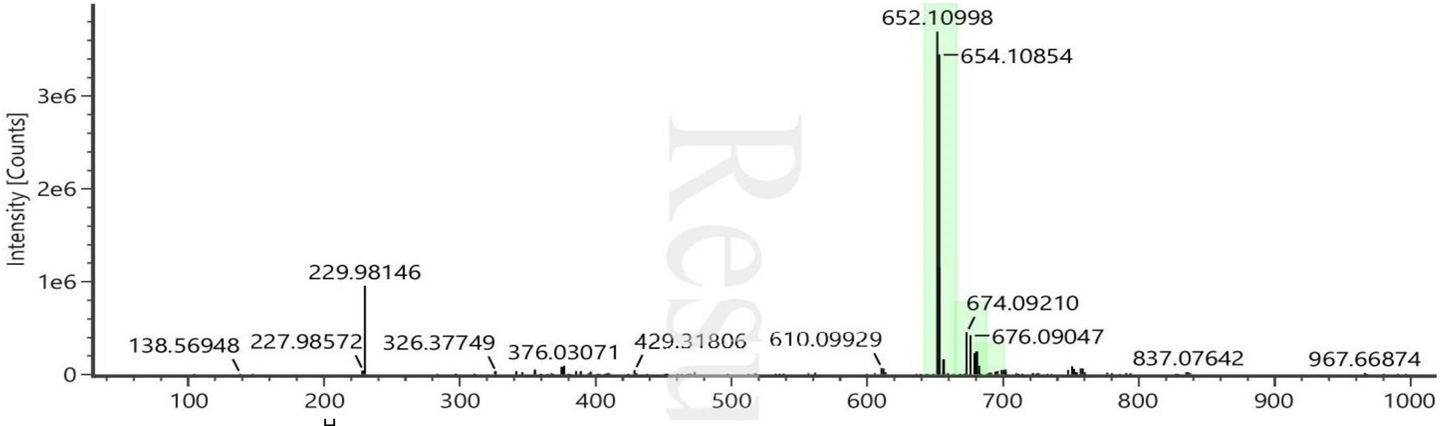

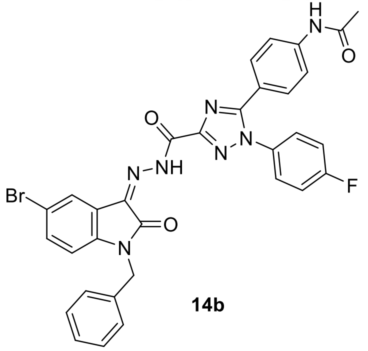

14b

Chemical Formula: $C_{32}H_{23}BrFN_7O_3$
Exact Mass: 651.1030

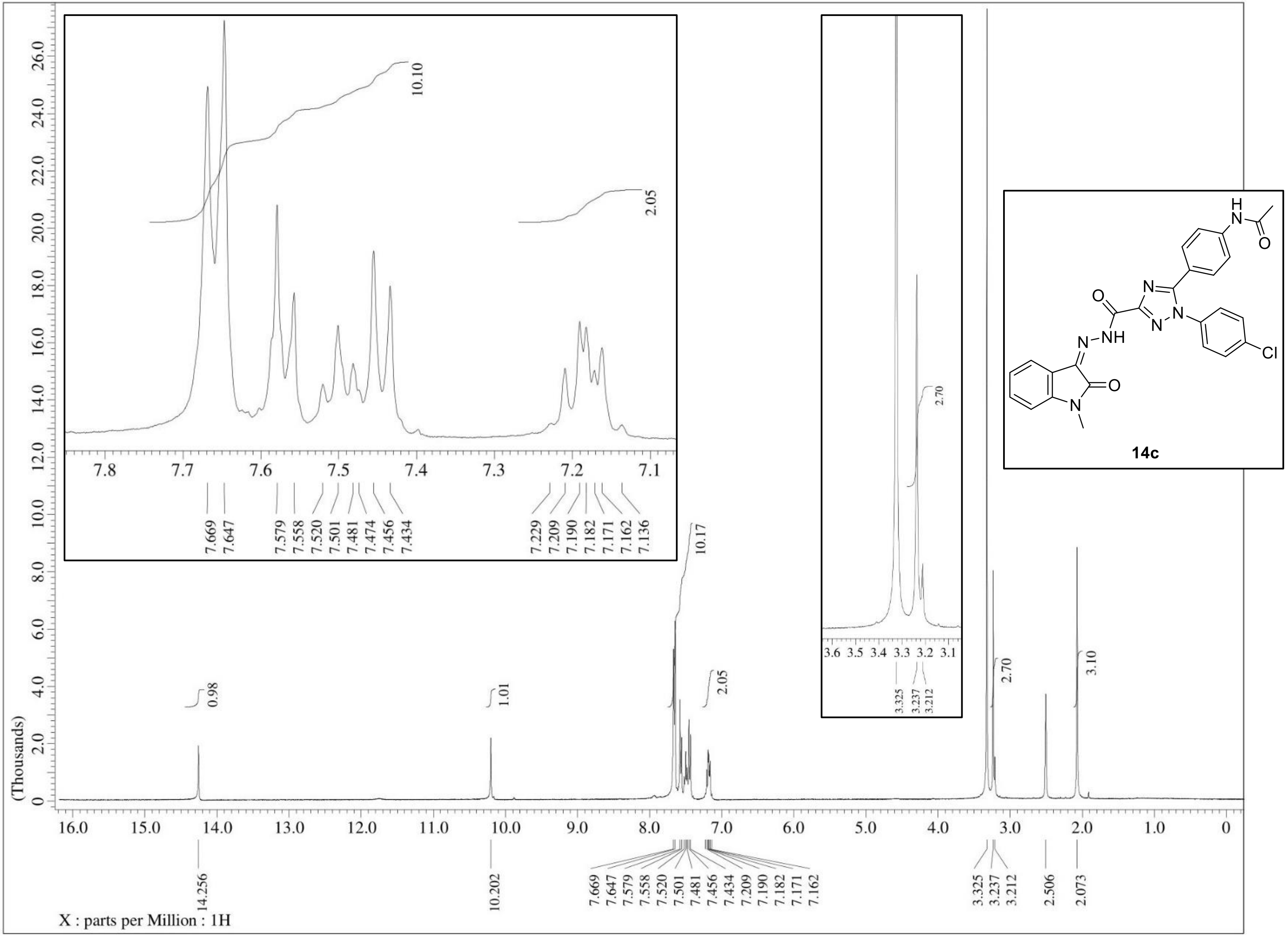

(Thousands)
X : parts per Million : 1H
14.256
10.202
7.669
7.647
7.579
7.558
7.520
7.501
7.481
7.456
7.434
7.209
7.190
7.182
7.171
7.162
3.325
3.237
3.212
2.506
2.073
0.98
1.01
10.17
2.05
2.70
3.10
7.474
7.229
7.136
10.10
N
NH
O
Cl
14c

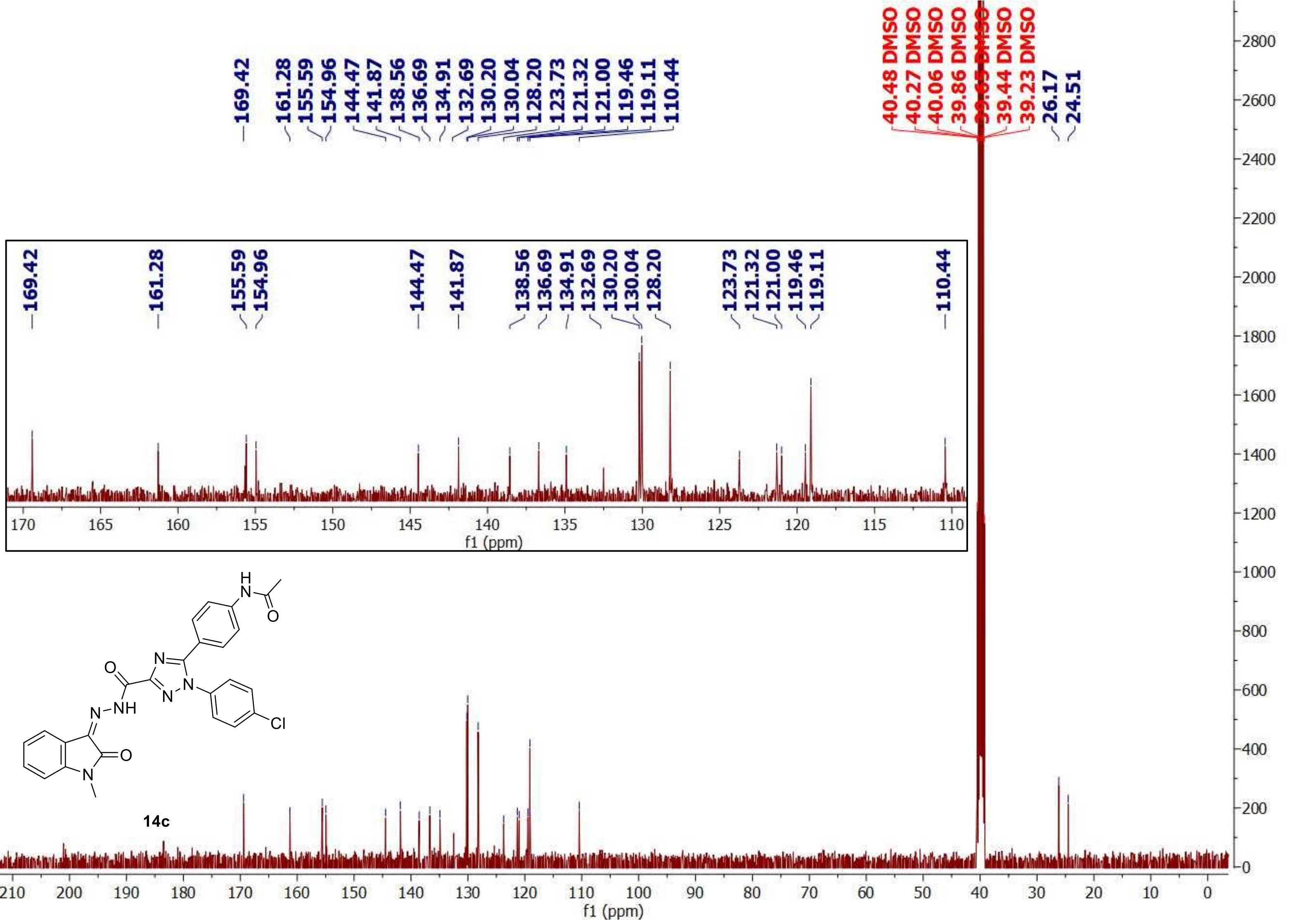

169.42
161.28
155.59
154.96
144.47
141.87
138.56
136.69
134.91
132.69
130.20
130.04
128.20
123.73
121.32
121.00
119.46
119.11
110.44
40.48 DMSO
40.27 DMSO
40.06 DMSO
39.86 DMSO
39.65 DMSO
39.44 DMSO
39.23 DMSO
26.17
24.51
f1 (ppm)
14c

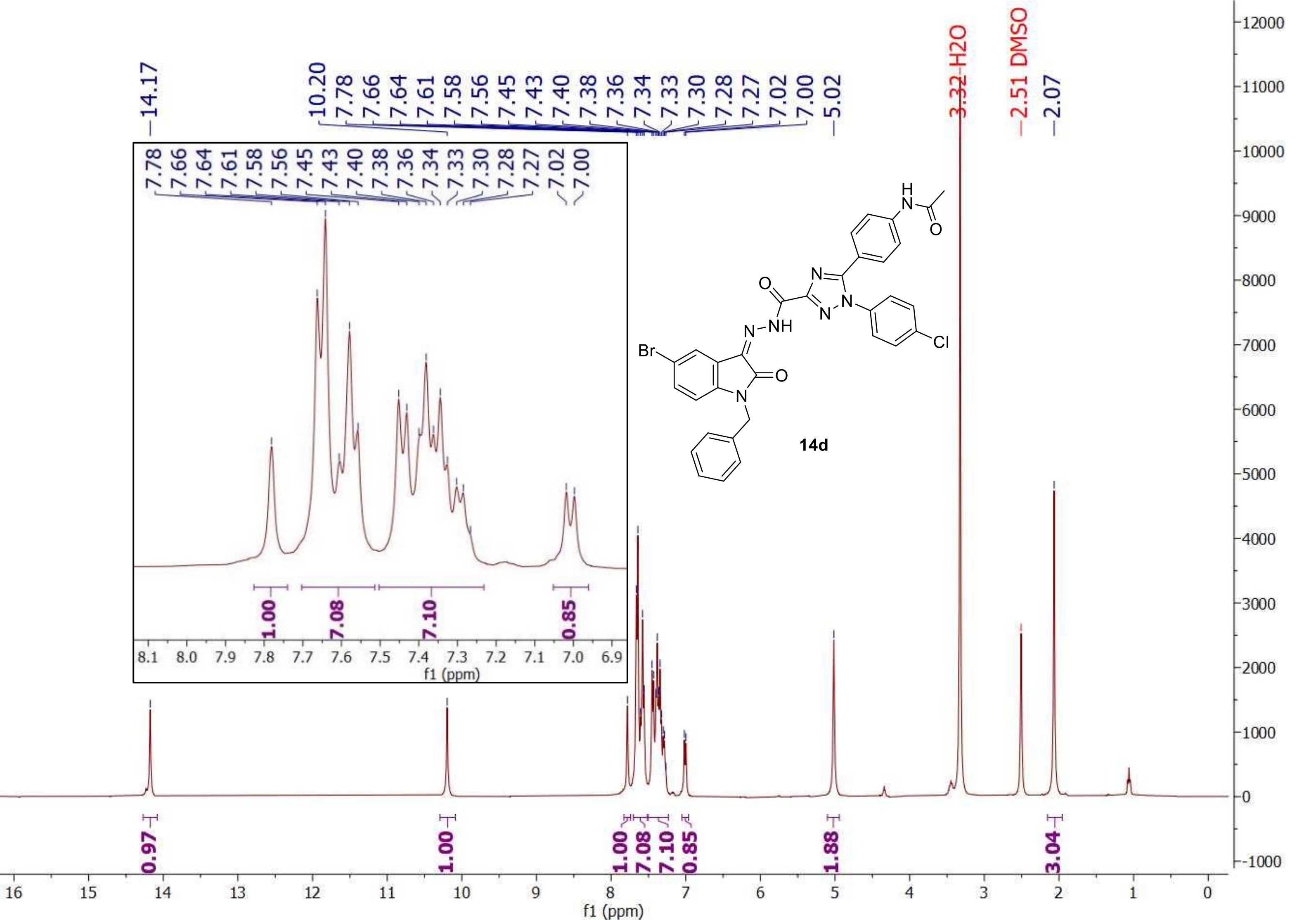
14.17
10.20 7.78 7.66 7.64 7.61 7.58 7.56 7.45 7.43 7.40 7.38 7.36 7.34 7.33 7.30 7.28 7.27 7.02 7.00
5.02
3.32 H2O
2.51 DMSO
2.07
1.00
7.08
7.10
0.85
f1 (ppm)
Br
N
O
N
NH
O
N
N
N
Cl
H
N
O
14d
0.97
1.00
1.00
7.08
7.10
0.85
1.88
3.04
f1 (ppm)

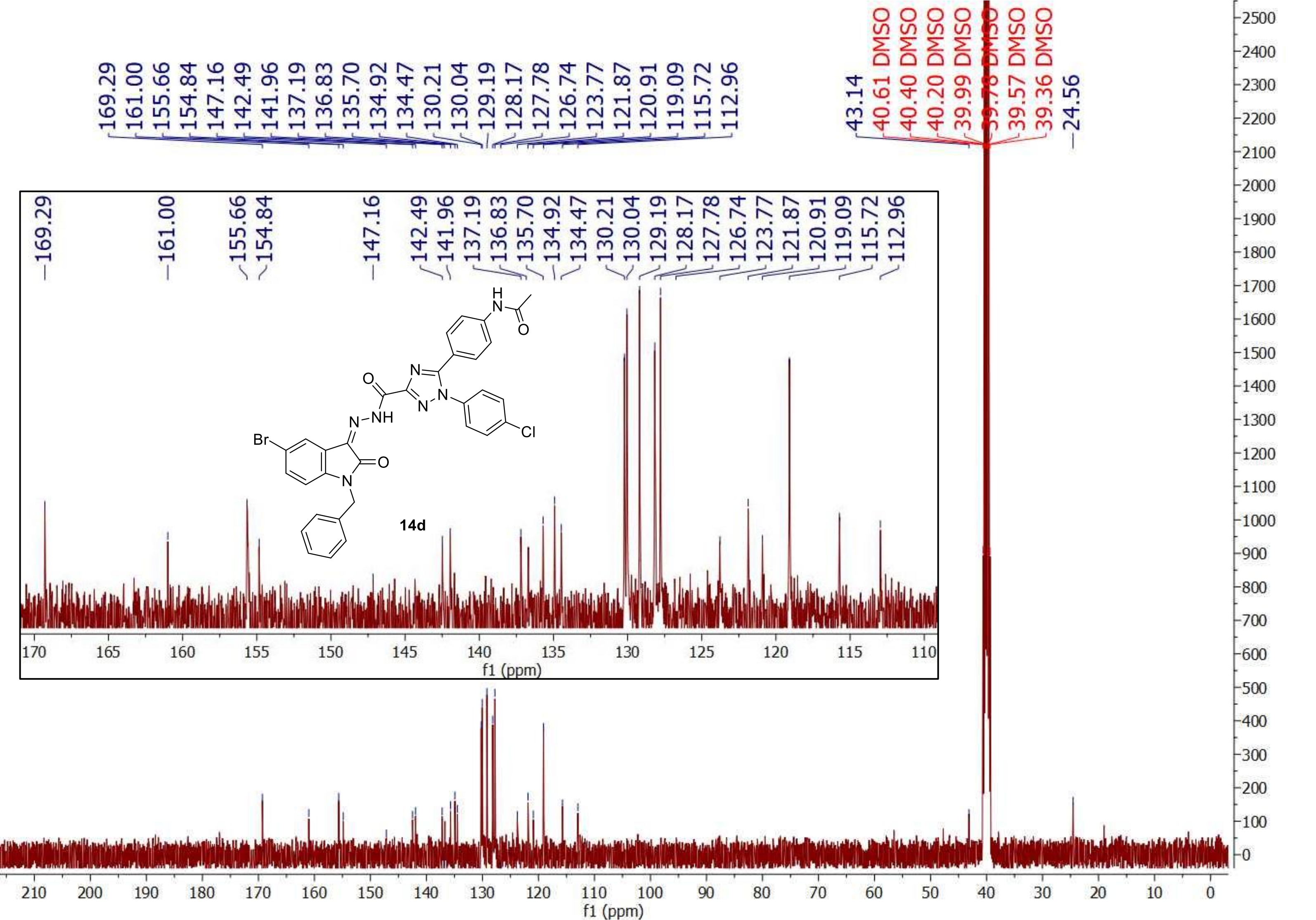

169.29
161.00
155.66
154.84
147.16
142.49
141.96
137.19
136.83
135.70
134.92
134.47
130.21
130.04
129.19
128.17
127.78
126.74
123.77
121.87
120.91
119.09
115.72
112.96
43.14
40.61 DMSO
40.40 DMSO
40.20 DMSO
39.99 DMSO
39.78 DMSO
39.57 DMSO
39.36 DMSO
24.56
Br
N
O
N
NH
O
N
N
N
H
N
O
Cl
14d
f1 (ppm)
f1 (ppm)
